# A Systematic Review and Meta-Analysis of Doxazosin Pharmacokinetics in Healthy and Diseased Populations

**DOI:** 10.3390/ph18121825

**Published:** 2025-11-29

**Authors:** Dania Fatima, Mohammed S. Alasmari, Yousef Alshomrani, Ammara Zamir, Faleh Alqahtani, Iltaf Hussain, Muhammad Fawad Rasool

**Affiliations:** 1 Department of Pharmacy Practice, Faculty of Pharmacy, Bahauddin Zakariya University, Multan 60800, Pakistan; daniafatima14@gmail.com (D.F.); ammarazamir20@gmail.com (A.Z.); 2 Security Forces Hospital Program, General Directorate of Medical Services, Ministry of Interior, Riyadh 11481, Saudi Arabiadr.yousef11111@gmail.com (Y.A.); 3 Department of Pharmacology and Toxicology, College of Pharmacy, King Saud University, Riyadh 11451, Saudi Arabia; 4 Center for Drug Safety and Policy, Xi’an Jiatong University, Xi’an 710061, China

**Keywords:** doxazosin, pharmacokinetics, systematic review, meta-analysis

## Abstract

**Background:** Doxazosin, an α_1_-adrenergic antagonist, is commonly used in the management of hypertension and benign prostatic hyperplasia (BPH). Pharmacokinetic (PK) variability across populations may affect drug exposure and clinical response. This systematic review and meta-analysis aimed to summarize PK differences and generate pooled estimates of key parameters, including area under the curve (AUC) and maximum plasma concentration (Cmax). **Methods:** A systematic search of Google Scholar, PubMed, ScienceDirect, and the Cochrane Library identified 25 eligible studies reporting doxazosin PK data. All extracted AUC and Cmax values were dose-normalized prior to synthesis to ensure comparability across different doses and formulations. A random-effects meta-analysis was performed using the metafor package in R to estimate pooled dose-normalized AUC and Cmax while accounting for between-study variability. Heterogeneity was assessed using the I^2^ statistic. Sensitivity analyses—including leave-one-out diagnostics and Baujat plots—were used to identify influential studies. Publication bias and small-study effects were evaluated through funnel plots, trim-and-fill procedures, and Egger’s regression test. Meta-regression analyses examined the influence of age and body weight on PK parameters. **Results:** The meta-analysis produced pooled dose-normalized estimates for AUC and Cmax, with high heterogeneity across studies (I^2^ ≈ 90%). Leave-one-out analyses demonstrated stable pooled estimates; for dose-normalized AUC, exclusion of three influential studies reduced heterogeneity to 82% with only a modest decrease in the pooled mean. Baujat plots identified a small number of studies as key contributors to heterogeneity, while most exerted minimal influence. Funnel plots showed notable asymmetry for both AUC and Cmax, and trim-and-fill analyses suggested possible small-study effects; however, adjusted pooled estimates remained consistent. Egger’s regression confirmed significant asymmetry for dose-normalized AUC (t = 4.41, *p* = 0.0003) and Cmax (t = 4.35, *p* = 0.0001). Meta-regression revealed that body weight significantly reduced Cmax, whereas age had no significant effect on either AUC or Cmax. **Conclusions:** This systematic review and meta-analysis provide a comprehensive evaluation of doxazosin PK across diverse populations. Despite normalization, substantial variability remained in AUC and Cmax, related in part to ethnicity, hepatic impairment, dosage formulation, and body weight. While pooled estimates offer valuable summary reference points, the high heterogeneity and evidence of small-study effects highlight the need for more standardized PK trials and patient-level analyses to better support individualized dosing strategies.

## 1. Introduction

Doxazosin is a quinazoline-derived alpha-1 adrenoceptor antagonist [1,2] that was initially approved by the U.S. Food and Drug Administration (FDA) in 1990 [3,4]. Later on, the CR dosage form was approved by the U.S. FDA in 2005 [5]. It is indicated for the treatment of hypertension and symptomatic management of benign prostatic hyperplasia (BPH) [3,6,7]. Additionally, doxazosin has demonstrated efficacy in facilitating the expulsion of ureteral stones [8]. Its blockade of alpha-1 receptors induces vasodilation of arterioles and veins, thereby reducing total peripheral resistance and lowering blood pressure [6,9]. In the context of BPH, its inhibition decreases urethral resistance and enhances urinary flow [10]. It is commercially available in both immediate-release (IR) and controlled-release (CR) tablet formulations.

Doxazosin belongs to class II under the Biopharmaceutics Classification System (BCS), with low solubility and high permeability [11,12]. It undergoes rapid absorption from the gastrointestinal tract following oral administration, with a bioavailability (F) of 65% [13]. This drug is 98–99% protein-bound, with preferential binding to alpha-1 acid glycoprotein and, in lesser extent to albumin [14,15]. Doxazosin undergoes extensive hepatic metabolism [13], mainly by the CYP3A4 enzyme, and to a lesser extent is metabolized by CYP2C9 and CYP2D6 [3]. Major pathways of metabolism are via hydroxylation and demethylation [16], with only 5% of the drug being excreted in unchanged form in urine [1,17], with a plasma clearance value of 1–2 mL/min/kg [17].

Doxazosin has a monoisotopic structure with a chemical formula of C_23_H_25_N_5_O_5_ [4]. It has a molecular weight of 451.5 g/mol. The water solubility of this drug is poor, i.e., 8 mg/mL, and the octanol-to-water partition coefficient (log p) is 2.1, with an acid dissociation constant (pKa) of 6.52 [18].

Doxazosin is classified as pregnancy category C [6], indicating that fetal risk cannot be ruled out based on available data. It is excreted into breast milk in low concentrations and demonstrates minimal placental transfer [19,20]. Owing to its physicochemical properties (high lipophilicity and low molecular weight), doxazosin crosses the blood–brain barrier easily [21]. Common adverse effects associated with the use of doxazosin are dizziness, headache, asthenia, vertigo, and tachycardia [22,23], and in rare cases, priapism can occur [4]. The pharmacokinetics and clinical considerations of doxazosin may vary in special populations, necessitating careful assessment in groups such as the elderly, pregnant or lactating women, and patients with hepatic or renal impairment.

This systematic review and meta-analysis aim to provide a comprehensive evaluation of the PK of doxazosin in humans. Although several narrative reviews have discussed the drug’s pharmacological properties [10,14,24,25,26], no prior work has offered a rigorous, systematic synthesis or a quantitative meta-analysis assessing comparative PK across healthy and diseased populations. The only earlier attempt, published in 1987, summarized PK findings from ten studies but lacked detailed evaluation of special populations and did not incorporate dose-adjusted comparisons [1]. The present review addresses this gap by systematically comparing the PK profiles of immediate-release (IR) and controlled-release (CR) formulations, examining inter-population variability—including ethnic differences—and evaluating PK characteristics across diverse dosing regimens. To enable meaningful comparison across studies using different doses, dose-normalized PK parameters were derived and synthesized in a meta-analysis of AUC and Cmax, allowing standardized assessment of exposure. In addition, the review summarizes available evidence on drug–drug interactions and identifies factors contributing to PK variability that may inform clinical dosing decisions. Finally, the consolidated data generated in this review provide a valuable foundation for future model-informed drug development and population PK modeling, particularly in special populations such as individuals with renal and hepatic impairment.

## 2. Results

### 2.1. Literature Search

**Figure 1:**
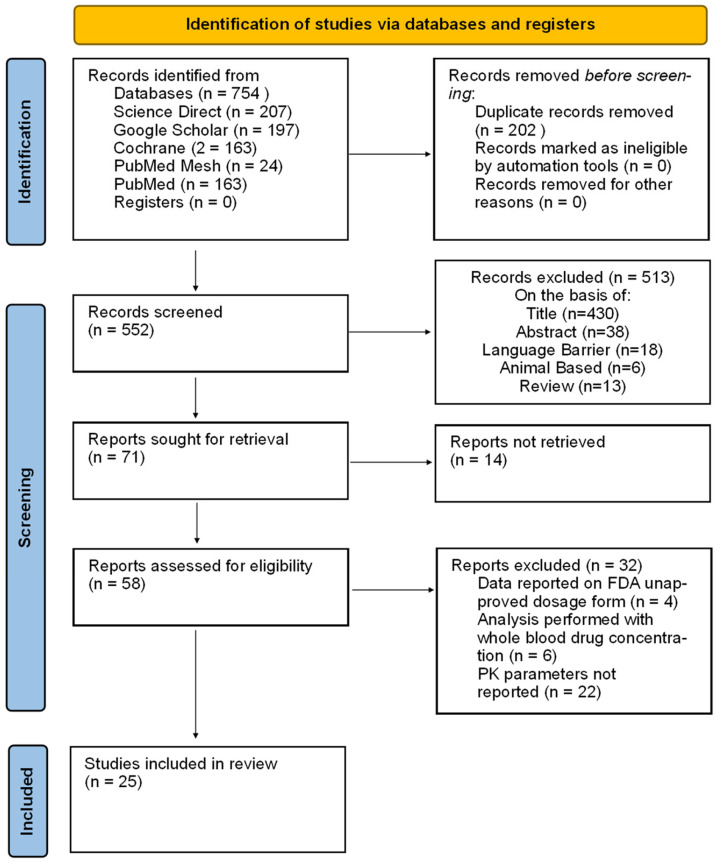
PRISMA flow diagram.

After searching across four databases, a total of 754 records were retrieved, of which 202 were removed in duplicates, and the final 25 studies were included in this review. Screening and inclusion details are illustrated in Figure 1.

### 2.2. Study Characteristics

The characteristics of retrieved studies, which include population, ethnicity, study size, age of the subjects, drug, dosage form, frequency, and method of assay, are presented in Supplementary Table S7.

### 2.3. Quality Assessment Results

All 25 studies were assessed for their quality. Among them, 17 studies were of low quality, 6 were of moderate quality, and only 2 of them were of high quality based on the JADAD scoring. The CASP scoring ranked all 25 articles as high quality. According to the CACPK scoring criteria, 23 studies were of high quality and 2 studies were of low quality. Based on the CCT scoring, seven studies were at high risk, nine were at moderate risk, and nine studies were at low risk.

### 2.4. Healthy Population

#### 2.4.1. Oral Administration

Among the included studies, 13 reported PK data in the healthy population. The area under the curve from 0 to infinity (AUC_0–∞_) was found to be 499.11 ± 129.93 ng·h/mL after administration of a 4 mg dose to healthy Thai volunteers [27]. Another study evaluated the PK of doxazosin at the same dose in healthy Chinese males (Table 1. PK parameters of immediate-release formulation in healthy population), where AUC_0–∞_ was higher, i.e., 781.1 ± 172.7 ng·h/mL [28]. In another study conducted on the Thai population, the reported C_max_ value was 18.39 (5.16) ng/mL, whereas the lower C_max_ was observed in the Caucasian population, i.e., 13.7 (2.9) ng/mL at a 2 mg administered dose [13,29]. A clinical study assessing the bioequivalence of two brands with an 8 mg depicted the C_max_ of the test and reference compound as 57.79 ± 20.14 ng/mL and 56.43 ± 19.66 ng/mL, respectively [30]. One study comparing reference (Cardura) and test (Dozozin-2) formulations at a 2 mg dose reported the total body clearance (CL_B_) as 2.65 ± 0.95 mL/min/kg and 2.70 ± 0.98 mL/min /kg, respectively [31].

In a multiple-dose study, the C_max_ of the standard formulation was displayed as 2.5-fold higher than the CR formulation, i.e., 29.3 ± 8.4 ng/mL vs. 11.3 ± 5.6 ng/mL, following administration of a 4 mg dose. Another study based on PK differences between CR and IR formulations mentioned an approximately 60% higher AUC for the IR dosage form, i.e., 878 (273) ng·h/mL vs. 504 (171) ng·h/mL at an 8 mg dose. In contrast, the T_max_ (time to reach C_max_) was found to be delayed with the administration of CR tablet, i.e., 9.1 (4.7) h vs. 3.9 (1.2) h. Gender and age had no effect on the PK of the CR dosage form [13]. The C_max_ was found to be higher with the IR 8 mg tablet in comparison to CR tablet at the same dose, i.e., 56.43 (19.66) ng/mL vs. 22.49 (5.30) ng/mL [30,32]. A study performed on healthy Korean subjects with 4 mg CR tablet reported a 45% increase in C_max_, i.e., 21.6 ± 4.3 ng/mL vs. 11.3 ± 5.6 ng/mL, with respect to the Caucasian population (Table 2. PK parameters of controlled-release formulation in healthy population) [33]. 

One of the clinical studies depicted the AUC_0–∞_ of doxazosin 2 mg and its fixed-dose combination containing 2 mg doxazosin and 5 mg finasteride as 190.3 ± 44.3 ng·h/mL and 188.8 ± 45.6 ng·h/mL, respectively [34]. Another cross-over study assessing chronopharmacokinetic behavior showed that T_max_ was prolonged, i.e., 5.60 ± 3.27 h vs. 3.46 ± 1.28 h after evening dosing [35].

#### 2.4.2. Pharmacokinetics of Enantiomers

The administration of 4 mg racemic CR doxazosin tablet to healthy males reported greater exposure, as measured by AUC_0–∞_ of S-(+) enantiomer compared to R-(−) enantiomer, with respective values of 195.334 ng·h/mL vs. 85.506 ng·h/mL for Subject 1, and 175.172 ng·h/mL vs. 78.8 ng·h/mL for Subject 2 [36]. The remaining PK parameters are given in Table 2. PK parameters of controlled-release formulation in healthy population.

**Table 1:** PK parameters of immediate-release formulation in healthy population.

Dose (mg)	AUC_0–inf_ (ng·h/mL)	C_max_ (ng/mL)	T_max_ (h)	t_½_ (h)	CL/F (ml/min/kg)	Refs.
1	NR	11.0 (2.2)	1.3 (0.3)	NR	NR	[37]
2	Morning Dose: NR	Morning Dose: 16.98 (4.86)	Morning Dose: 3.46 (1.28)	Morning Dose: 19.52 (3.88)	Morning Dose: 2.21 (0.75)	[35]
	Evening Dose: NR	Evening Dose: 15.76 (4.16)	Evening Dose: 5.60 (3.27)	Evening Dose: 18.77 (3.47)	Evening Dose: 1.97 (0.60)	
2	Fasted: 195 (45)	Fasted: 13.7 (2.9)	Fasted: 2 (0.8)	Fasted: 14 (3.6)	NR	[13]
4	379 (131)	29.3 (8.4)	3.7 (1.5)	NR	NR	
8	878 (273)	66.8 (17.6)	3.9 (1.2)	20.5 (6.1)	NR	
2 (Reference Formulation)	289.76 (295.27)	17.97 (4.44)	1.9 (0.8)	13.8 (11.1)	CL_B_: 2.65(0.95)	[31]
2 (Test Formulation)	264.87 (163.09)	18.39 (5.16)	2.1 (1.2)	12.7 (7.4)	CL_B_: 2.70(0.98)	
2	311.48 (186.95)	18.39 (5.16)	2.1 (1.2)	16.7 (9.1)	NR	[29]
4 (Reference Formulation)	781.1 (172.7)	55.9 (7.3)	2.6 (1.1)	18.8 (5.5)	NR	[28]
4 (Test Formulation)	796.2 (145.7)	56.6 (7.7)	3 (1.0)	20.9 (5.9)	NR	
4 (Reference Formulation)	499.11 (129.93)	41.53 (10.85)	1.71 (0.81)	10.21 (1.83)	NR	[27]
4 (Test Formulation)	475.7 (109.75)	40.62 (10.56)	1.58 (0.56)	10.34 (1.41)	NR	
4	NR	10.3 (6.5)	10.5 (4.1)	NR	NR	[38]
8 (Reference Formulation)	2040 (905.6)	56.43 (19.66)	4.16 (2.301)	23.23 (5.65)	NR	[30]
8 (Test Formulation)	2226.3	57.79 (20.14)	4.39 (2.23)	25.46 (5.22)	NR	
2 (Reference Formulation)	190.3 (44.3)	16.3 (3.6)	1.4 (0.6)	11.5 (2.4)	NR	[34]
Dox 2 mg/Fin 5 mg (Test Formulation)	188.8 (45.6)	14.9 (3.3)	2.4 (1.4)	12.3 (2.6)	NR	

Data presented as mean (standard deviation); AUC_0–inf_: area under the plasma concentration–time curve from 0 to infinity; C_max_: peak plasma concentration; CL/F: oral clearance; CL_B_: total body clearance; Dox: doxazosin; Fin: finasteride; NR: not reported; Refs: references; T_max_: time to reach peak plasma concentration; t_½_: plasma elimination half-life.

**Table 2:** PK parameters of controlled-release formulation in healthy population.

Dose (mg)	AUC_0–inf_ (ng·h/mL)	C_max_ (ng/mL)	T_max_ (h)	t_½_ (h)	Refs.
8	Fasted: 569 (187)	Fasted: 18.3 (5.9)	Fasted: 14 (4)	Fasted: 15 (2.5)	[13]
	Fed: 670 (234)	Fed: 24 (7.1)	Fed: 11 (2)	Fed: 16.1 (2.7)	
4	201 (86)	11.3 (5.6)	8.2 (3.7)	NR	
8	504 (171)	28 (12.1)	9.1 (4.7)	18.6 (3.70)	
4 mg * 2 ^a^	735	22.5	15 (4)	16.5 (2.8)	
8	780	25.4	15 (4)	15.9 (2.2)	
4	Young Male: 308 (102)	Young Male: 15.9 (5.2)	Young Male: 7.8 (3.6)	Young Male: 20.3 (3.5)	
4	Young Female: 368 (230)	Young Female: 19.3 (11.5)	Young Female: 10.4 (3.0)	Young Female: 20.1 (3.2)	
4	Elderly Male: 409 (109)	Elderly Male: 20.4 (4.4)	Elderly Male: 13 (6.5)	Elderly Male: 23.4 (4.9)	
4	Elderly Female: 429 (91)	Elderly Female: 20.7 (3.9)	Elderly Female: 13.8 (4.7)	Elderly Female: 24.7 (4.9)	
8 (Reference Formulation)	NR	22.49 (5.30)	14.00 (2.83)	17.79 (3.69)	[32]
8 (Test Formulation)	NR	23.97 (8.78)	14.00 (4.60)	18.27 (2.99)	
4	Male: 372.9 (61.5)	Male: 21.6 (4.3) ^b^	Male: 5.5 (2.3)	Male: 38.7 (9.7)	[33]
4	Female: 361.2 (69.1)	Female: 20.3 (4.9) ^b^	Female: 5.6 (1.5)	Female: 26.3 (14.2)	
4 (Volunteer 1)	R-enantiomer: 85.506	R-enantiomer: 2.909	R-enantiomer: 14.04	R-enantiomer: 10.54	[36]
	S-enantiomer: 195.334	S-enantiomer: 5.983	S-enantiomer: 14.04	S-enantiomer: 14.683	
4 (Volunteer 2)	R-enantiomer: 78.8	R-enantiomer: 2.374	R-enantiomer: 14.19	R-enantiomer: 28.146	
	S-enantiomer: 175.172	S-enantiomer: 4.468	S-enantiomer: 14.19	S-enantiomer: 31.875	

Data presented as mean (standard deviation); AUC_0–inf_: area under the plasma concentration–time curve from 0 to infinity; C_max_: peak plasma concentration; NR: not reported; Refs: references; T_max:_ time to reach peak plasma concentration; t_½_: plasma elimination half-life. ^a^ dose is administered as two tablets of 4 mg; ^b^ C_max_ value given for steady state. * AUC given as AUC_0–24_.

### 2.5. Diseased Population

#### 2.5.1. Patients with Liver Cirrhosis

An open-label study evaluating the difference in PK of doxazosin with administration of a single 2 mg dose reported a 43% rise in AUC_0–∞_ among cirrhotic patients in comparison to healthy individuals, i.e., 246 ± 84.0 ng·h/mL vs. 172 ± 61.0 ng·h/mL [39]. The remaining PK parameters are presented in Table 3. PK parameters of doxazosin in diseased population.

#### 2.5.2. Hypertensive Patients

In mild to moderate hypertensive patients, AUC_0–∞_ after a single 2 mg dose was recorded as 287.2 ± 104.8 ng·h/mL, whereas at steady state it increased to 372.6 ± 136.3 ng·h/mL (at week 1) [45]. In another clinical study, a dose-proportional increase was observed in C_max_ over a dose range of 1–8 mg [46] and 1–16 mg [41].

#### 2.5.3. Patients with Renal Impairment

One study reported AUC_0–∞_ values in Group 1 (healthy subjects), Group 2 (renally impaired patients, not on hemodialysis), and Group 3 (renally impaired patients on hemodialysis investigated between two hemodialysis sessions) as 132 ± 25 ng·h/mL, 168 ± 21 ng·h/mL, and 165 ± 39 ng·h/mL, respectively [40]. Another clinical study reported AUC/dose as 162 ± 59 ng·h/mL after administering a 1 mg dose to renal-insufficient patients [47].

### 2.6. Drug–Drug Interactions

Among the included studies, only three of them reported the PK of doxazosin when given in combination with other drugs. The steady-state C_max_ of doxazosin alone (2 mg) and in combination therapy with nifedipine (20 mg) was 23.5 ± 6.0 ng/mL vs. 20.2 ± 3.2 ng/mL, respectively, with no significant difference observed [48]. Co-administration with MDMA (3,4-methylenedioxymethamphetamine), doxazosin reduced the AUC (51.9 ± 4.6 ng·h/mL vs. 44.3 ± 3.2 ng·h/mL) of MDA (3,4-methylenedioxyamphetamine), a metabolite of MDMA [49]. A clinical study reported the oral clearance (CL/F) of doxazosin with a 1 mg administered dose at steady state as 161.667 mL/min, and after administration with 10 mg enalapril as 176.667 mL/min, with no major PK interaction being reported between two drugs [50]. (Table 4. PK parameters of drug–drug and drug–food interactions of doxazosin).

### 2.7. Drug–Food Interaction

One study from the literature search investigating food effects demonstrated an 18% increase in AUC (670 ± 234 ng·h/mL vs. 569 ± 187 ng·h/mL) when an 8 mg CR tablet was given after a high-fat meal as compared to the fasted condition. T_max_ decreased from 14 h to 11 h in the fed condition [13].

### 2.8. Meta-Analysis

**Figure 2:**
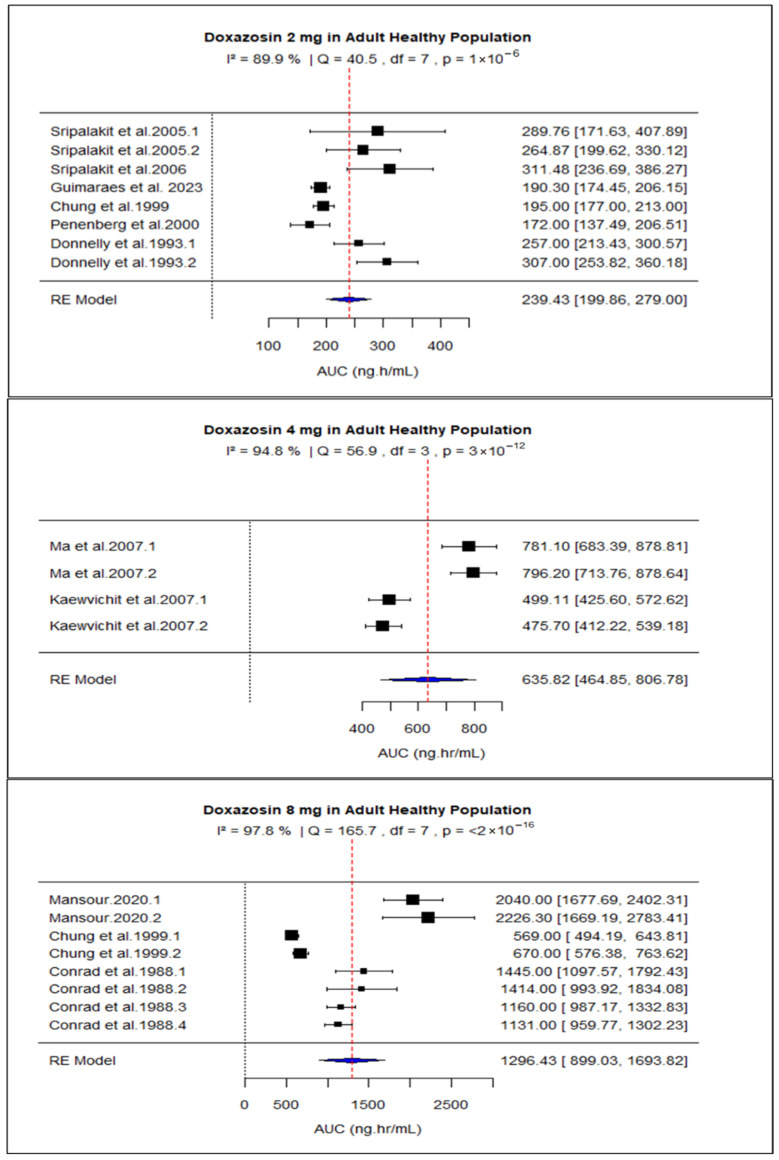
Forest plot of AUC for 2, 4, and 8 mg doxazosin in adult healthy population. The figure display means of AUC and sample size (black square and its size) and 95% CI (the horizontal lines) for adult healthy population across studies, with the blue diamond at the bottom showing the overall pooled AUC estimate and its CI. The vertical red dashed line is a reference for the overall pooled AUC estimate, allowing for a visual comparison across studies [13,27,28,29,30,31,34,39,44,48,51].

**Figure 3:**
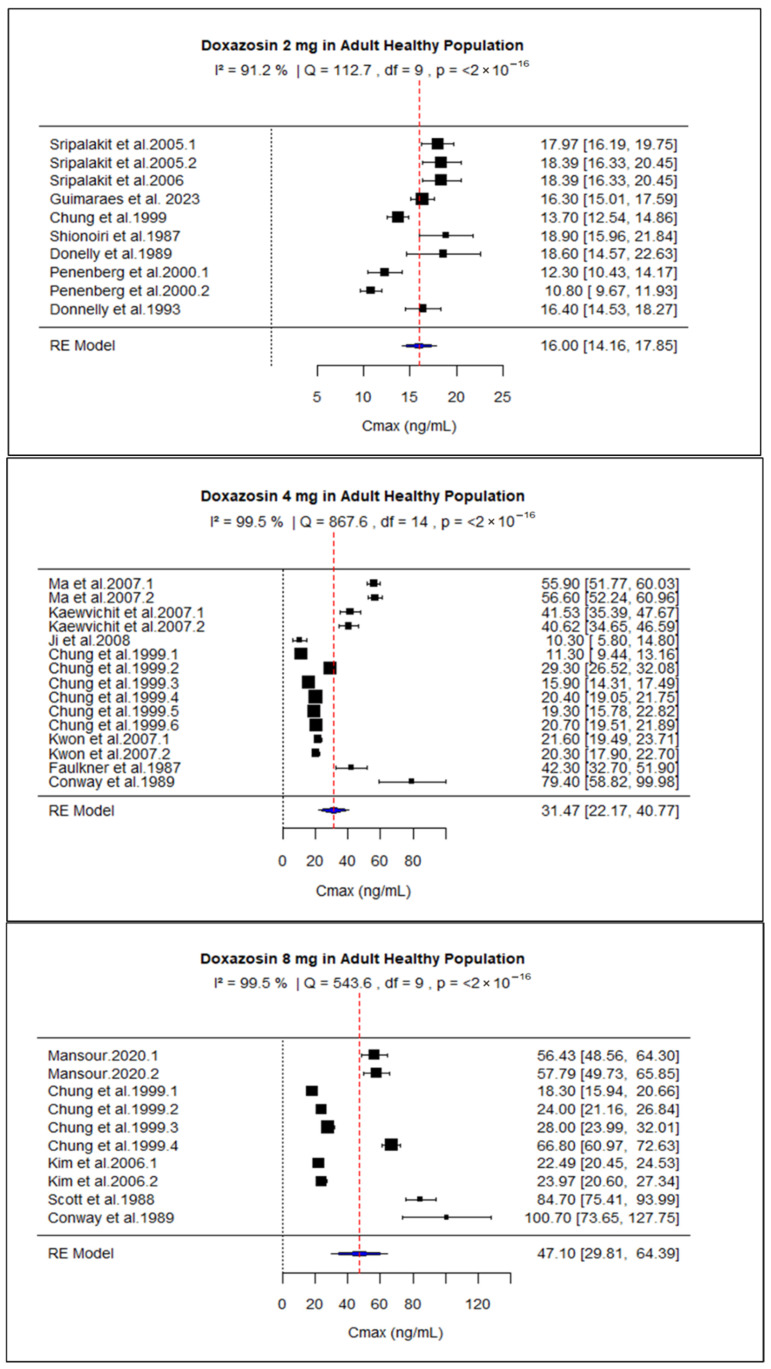
Forest plot of C_max_ for 2, 4, and 8 mg doxazosin in adult healthy population. Each figure displays mean of C_max_ and sample size (black square and its size) and 95% CI (the horizontal lines) for adult healthy population across studies, with the blue diamond at the bottom showing the overall pooled C_max_ estimate and its CI. The vertical red dashed line is a reference for the overall pooled C_max_ estimate, allowing for a visual comparison across studies [13,27,28,29,30,31,32,33,34,38,39,41,42,43,45,46,51].

**Figure 4:**
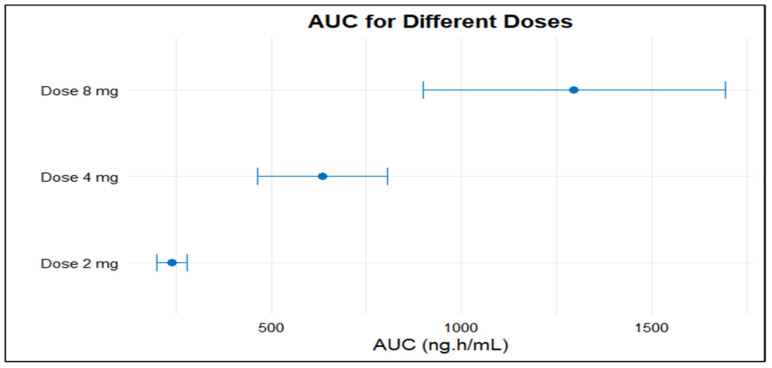
AUC with 95% CI for three different oral doses of doxazosin (2 mg, 4 mg, and 8 mg). Each horizontal line represents the upper and lower values of the 95% CI around the mean AUC, with the point estimate shown as a solid circle. Higher doses are associated with increased systemic exposure as reflected by the rising AUC values.

**Figure 5:**
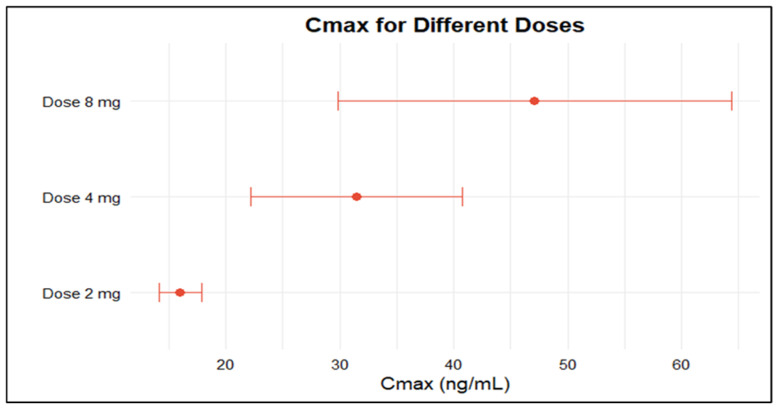
C_max_ with 95% confidence intervals for three different oral doses of doxazosin (2 mg, 4 mg, and 8 mg). Each horizontal line represents the upper and lower values of the 95% CI around the mean C_max_, with the point estimate shown as a solid circle. Higher doses are associated with increased systemic exposure as reflected by the rising C_max_ values.

Due to data availability and consistency in reporting, the meta-analysis was initially limited to studies in healthy participants receiving 2 mg, 4 mg, or 8 mg doses of doxazosin, as these met the predefined criteria for sample size and quantitative PK reporting. The pooled AUC for 2 mg, 4 mg, and 8 mg doses was 239.43 (95% CI: 199.86–279.00), 635.82 (95% CI: 464.85–806.78), and 1296.43 (95% CI: 899.03–1693.82), respectively (Figure 2). The pooled Cmax values were 16.00 (95% CI: 14.16–17.85), 31.47 (95% CI: 22.17–40.77), and 47.10 (95% CI: 29.81–64.39) for the same doses (Figure 3). Overall, the analysis revealed significant heterogeneity among the included studies, as indicated by consistently high I^2^ values. Both AUC and Cmax increased proportionally with dose, confirming the dose-dependent rise in systemic exposure (Figure 4 and Figure 5).

### 2.9. Subgroup Analysis

**Figure 6:**
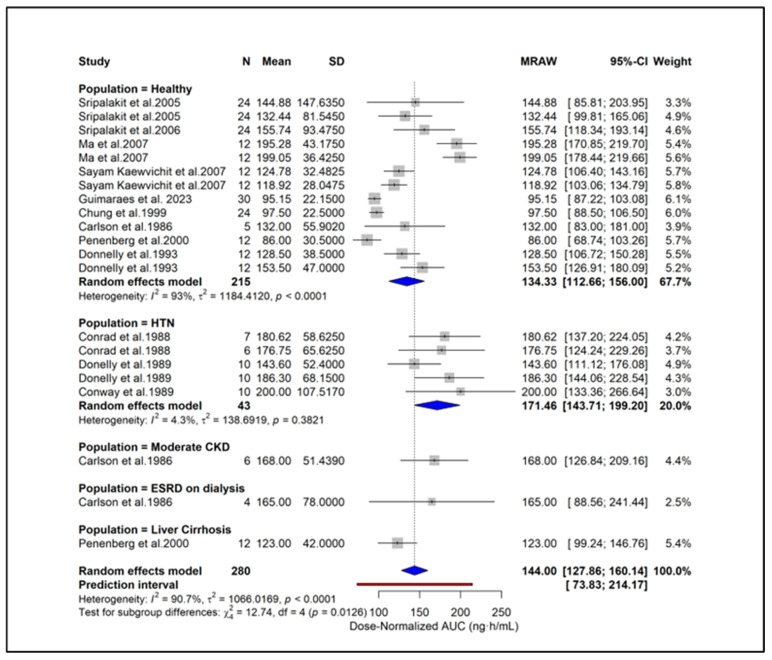
**Subgroup analysis of dose-normalized AUC across clinical populations.** The figure presents pooled mean AUC/dose estimates across studies within each population subgroup. Squares represent study-level effects with their size proportional to study weight. Diamonds indicate subgroup pooled estimates, and the overall diamond shows the total pooled effect. Heterogeneity and tests for subgroup differences are also reported [13,27,28,29,31,34,39,40,44,45,46,51].

**Figure 7:**
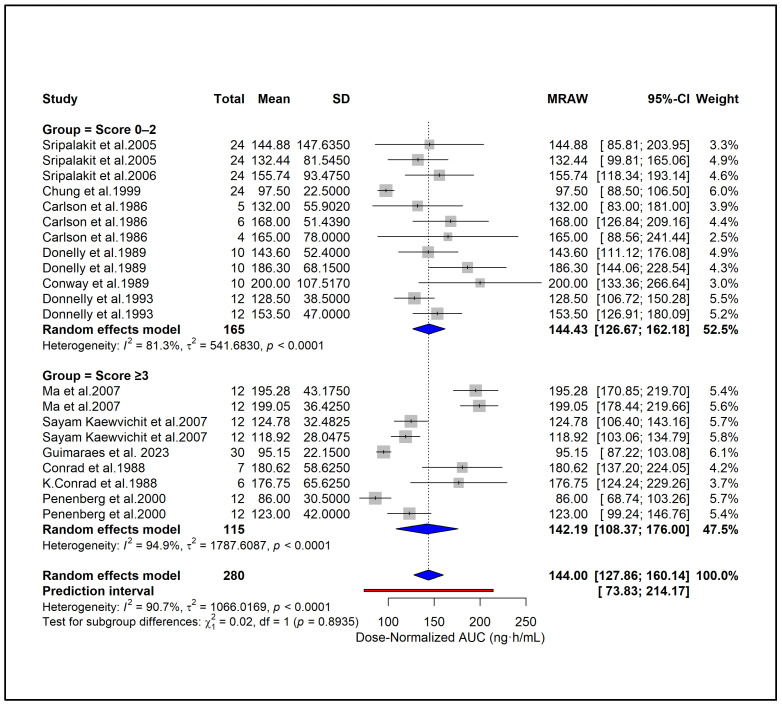
**Subgroup analysis of dose-normalized AUC by study quality score.** Dose-normalized exposure was similar across low- and high-quality studies, with no significant subgroup difference (*p* = 0.8935) [13,27,28,29,31,34,39,40,44,45,46,51].

**Figure 8:**
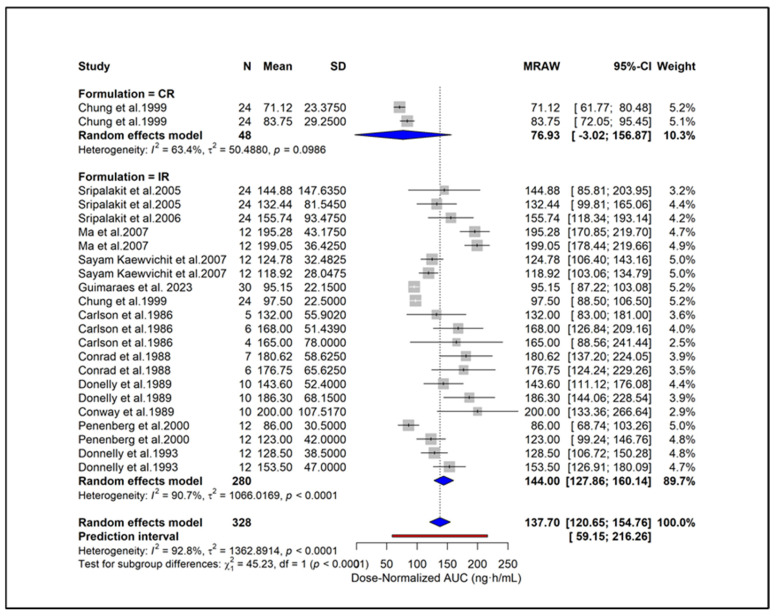
**Subgroup analysis of dose-normalized AUC by formulation.** CR formulations showed lower exposure (76.93 ng·h/mL) compared with IR formulations (144.00 ng·h/mL), with a significant subgroup difference (*p* < 0.0001) [13,27,28,29,31,34,39,40,44,45,46,51].

**Figure 9:**
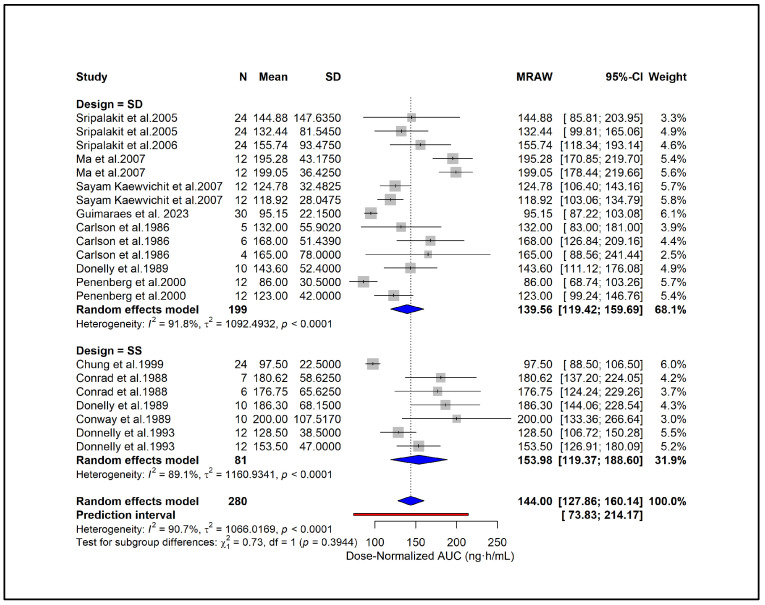
**Subgroup analysis of dose-normalized AUC by study design.** Dose-normalized exposure was comparable between single-dose and steady-state, with no significant subgroup difference (*p* = 0.3944) [13,27,28,29,31,34,39,40,44,45,46,51].

**Figure 10:**
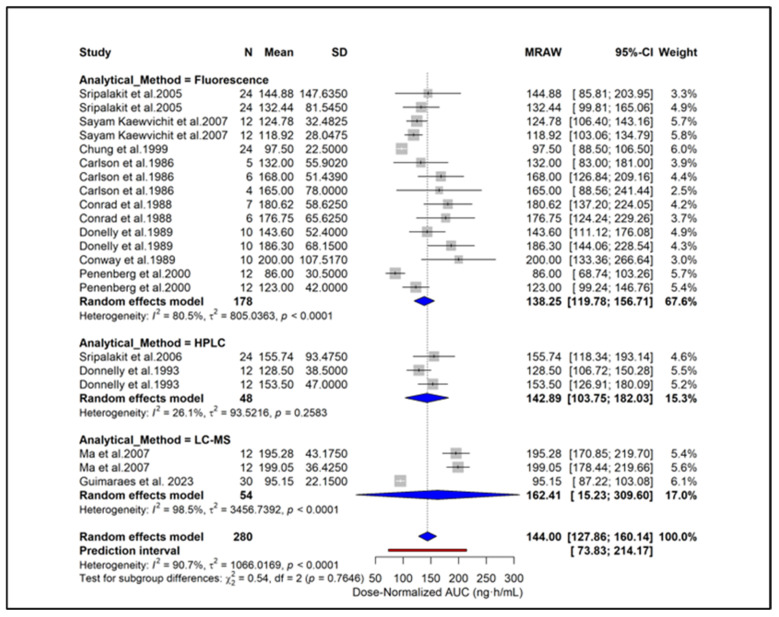
**Subgroup analysis of dose-normalized AUC by analytical method.** Dose-normalized exposure was similar across fluorescence, HPLC, and LC–MS techniques, with no significant subgroup difference (*p* = 0.7646) [13,27,28,29,31,34,39,40,44,45,46,51].

**Figure 11:**
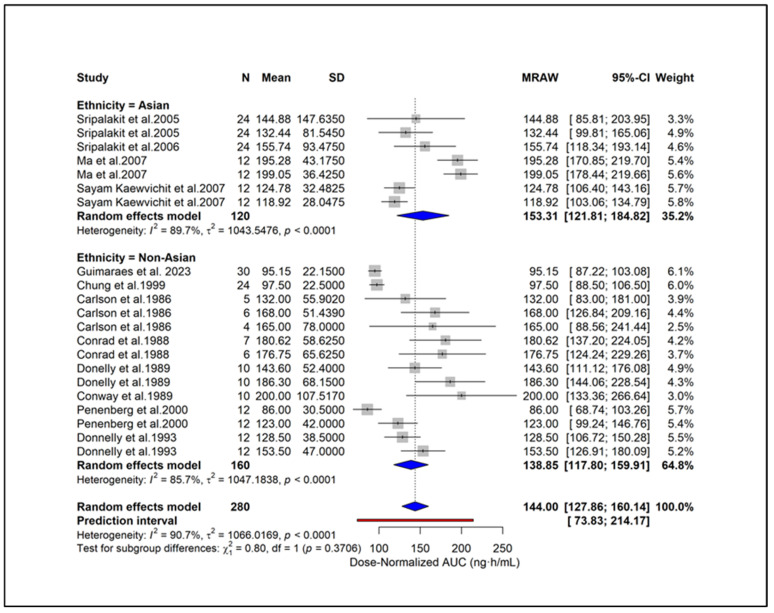
**Subgroup analysis of dose-normalized AUC by ethnicity.** Asian show slightly higher exposure compared to non-Asian, but with no significant subgroup difference (*p* = 0.3706) [13,27,28,29,31,34,39,40,44,45,46,51].

To enable standardized comparison across studies using different dosing regimens, all PK parameters were subsequently converted to dose-normalized values, and additional analyses were conducted. Subgroup analysis showed higher dose-normalized AUC in hypertension (171.46 ng·h/mL), moderate CKD (168.0 ng·h/mL), and ESRD (165.0 ng·h/mL) compared with healthy subjects (134.33 ng·h/mL) (Figure 6). Liver cirrhosis also showed elevated exposure (123.0 ng·h/mL) despite dose adjustment. A significant subgroup difference (*p* = 0.0126) indicates that underlying clinical conditions contribute to PK variability independent of dose. Subgroup analysis based on study quality scores showed similar dose-normalized AUC estimates between studies with scores 0–2 (144.43 ng·h/mL) and those with scores ≥ 3 (142.19 ng·h/mL) (Figure 7). No significant subgroup difference was observed (*p* = 0.8935), indicating that study quality did not materially influence dose-normalized exposure estimates. Based on formulations, subgroup analysis showed lower dose-normalized AUC for CR (76.93 ng·h/mL) compared with IR formulations (144.00 ng·h/mL) (Figure 8). The subgroup difference was statistically significant (*p* < 0.0001), indicating that even after dose normalization, IR formulations produce higher systemic exposure than CR formulations. Subgroup analysis by study design showed similar dose-normalized AUC values between single-dose (139.56 ng·h/mL) and steady-state studies (153.98 ng·h/mL) (Figure 9). No significant subgroup difference was detected (*p* = 0.3944), indicating that study design did not meaningfully impact dose-normalized exposure. Subgroup analysis by analytical method showed comparable dose-normalized AUC estimates across fluorescence (138.25 ng·h/mL), HPLC (142.89 ng·h/mL), and LC–MS (162.41 ng·h/mL) (Figure 10). No significant subgroup difference was observed (*p* = 0.7646), indicating that the method of quantification did not materially influence dose-normalized exposure. Subgroup analysis by ethnicity showed slightly higher dose-normalized AUC in Asian studies (153.31 ng·h/mL) compared with non-Asian studies (138.85 ng·h/mL) (Figure 11). However, the difference was not statistically significant (*p* = 0.3706), indicating that ethnicity did not meaningfully affect dose-normalized exposure. Corresponding subgroup analysis and figures for dose-normalized Cmax appear in the Supplementary Materials, Figures S1–S5.

**Figure 12:**
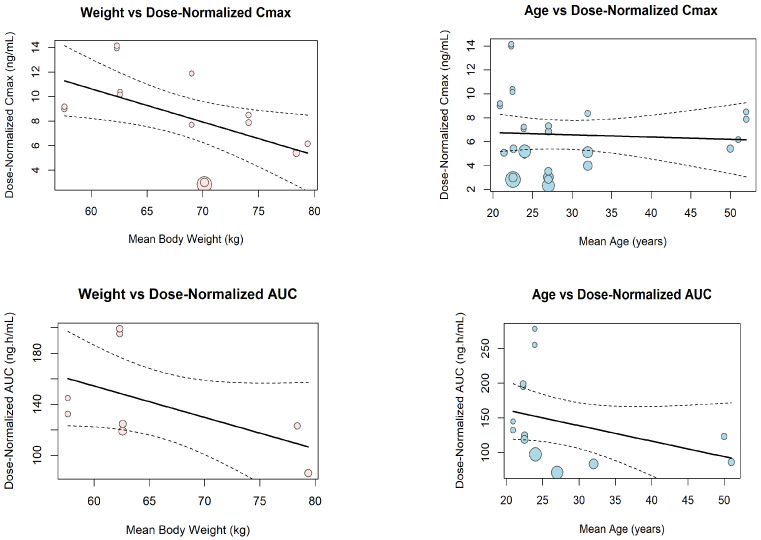
**Meta-regression analyses evaluating the effects of body weight and age on dose-normalized Cmax and AUC.** Body weight showed a significant negative association with Cmax but no effect on AUC, while age demonstrated no significant influence on either pharmacokinetic parameter across the included studies.

A meta-regression analysis was conducted to explore the influence of key patient covariates on dose-normalized pharmacokinetic parameters (Figure 12). Body weight showed a significant negative association with dose-normalized Cmax (β = −0.2768, *p* = 0.017), indicating that higher weight was associated with lower exposure. In contrast, age demonstrated no significant relationship with dose-normalized Cmax (β = −0.0185, *p* = 0.77), with a nearly flat regression line indicating no meaningful effect. For dose-normalized AUC, neither age (β = −2.24, *p* = 0.17) nor body weight (β = −2.45, *p* = 0.13) were significant predictors, though both showed a weak negative trend.

**Figure 13:**
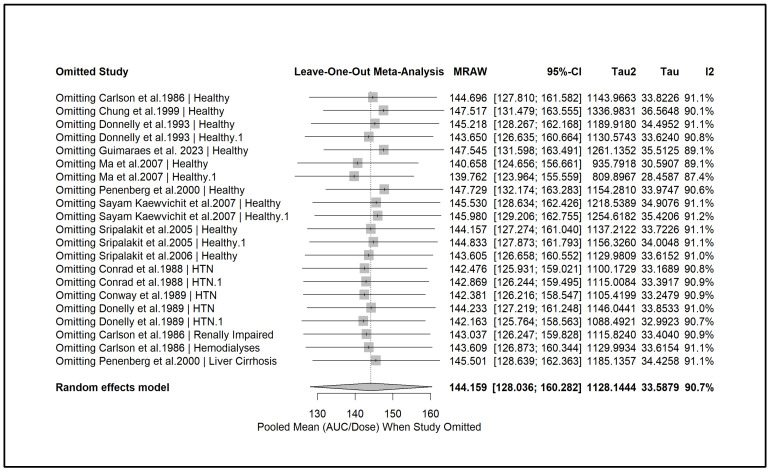
**Leave-one-out sensitivity analysis for exposure.** Sequential exclusion of individual studies showed that removal of most studies did not materially change the pooled mean AUC or its confidence interval. Excluding three influential studies reduced heterogeneity (I^2^) to below 90%, but the pooled mean AUC remained stable, supporting the robustness of the overall findings [13,27,28,29,31,34,39,40,44,45,46,51].

A leave-one-out sensitivity analysis was conducted (Figure 13) to evaluate the influence of individual studies on the pooled estimates. For AUC, the pooled mean remained stable across iterations, with heterogeneity (I^2^) decreasing below 90% only upon the exclusion of three influential studies. In contrast, for Cmax, sequential removal of individual studies did not meaningfully reduce heterogeneity, which consistently remained above 90%, and the pooled estimates showed minimal change (Supplementary Materials, Figure S6). These findings indicate that no single study exerted a disproportionate influence on the overall results.

**Figure 14:**
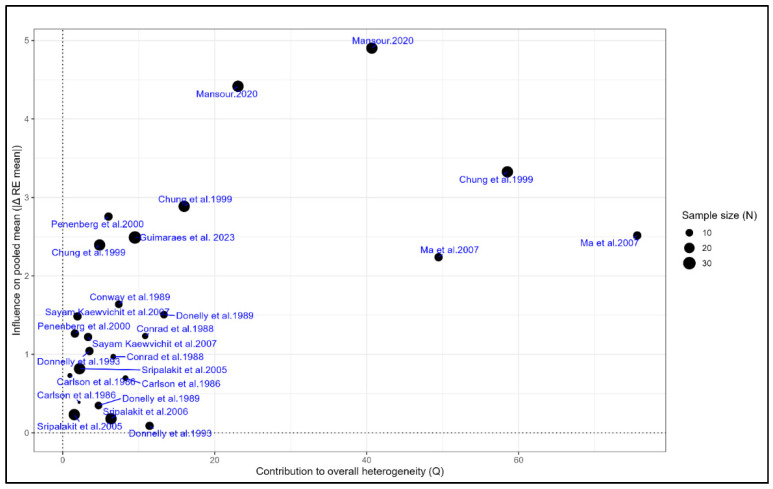
**Baujat plot for AUC illustrating each study’s contribution to heterogeneity and influence on the pooled mean.** The x-axis represents the contribution to overall heterogeneity (Q), while the y-axis shows the influence on the pooled mean AUC. Point size reflects study sample size. Mansour et al. (2020) [30], Ma et al. (2007) [28], Peneberg et al. (2000) [39], and Chung et al. (1999) [13] showed the greatest influence, whereas the remaining studies contributed minimally [27,29,31,34,40,44,45,46,51].

Baujat plots were used to assess the contribution of individual studies to overall heterogeneity and their influence on the pooled estimates. For AUC, Mansour et al. (2020) [30], Ma et al. (2007) [28], Peneberg et al. (2000) [39], and Chung et al. (1999) [13] were identified as the primary contributors to heterogeneity and impact on the pooled mean, with the remaining studies demonstrating limited influence (Figure 14). For Cmax, studies by Ma et al. (2007) [28], Peneberg et al. (2000) [39], Conard et al. (1988) [44], and Chung et al. (1999) [13] exerted the greatest influence, whereas all other studies contributed minimally, supporting the robustness of the pooled Cmax results (Supplementary Materials, Figure S7).

**Figure 15:**
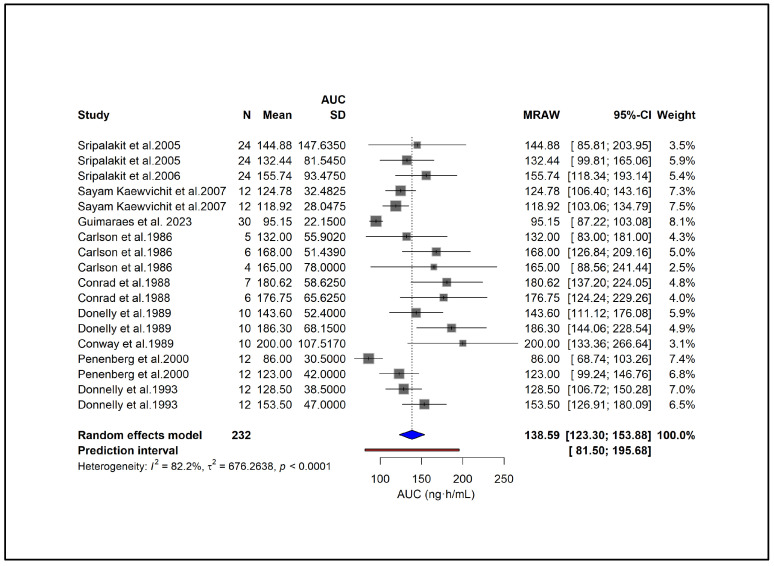
**Forest plot of the meta-analysis for AUC after excluding studies with the greatest contribution to heterogeneity and overall influence on pooled estimates.** Removal of these influential studies reduced heterogeneity to I^2^ = 82%, with a small decrease in the pooled mean AUC, supporting the robustness of the findings [27,29,31,34,39,40,44,45,46,51].

After excluding the most influential studies (Figure 15), the pooled mean AUC decreased slightly from 144 to 138.6, while heterogeneity improved to I^2^ = 82%. Despite this modest reduction, the overall interpretation of the results remained unchanged, indicating that the pooled AUC estimate is robust and not unduly driven by these studies.

**Figure 16:**
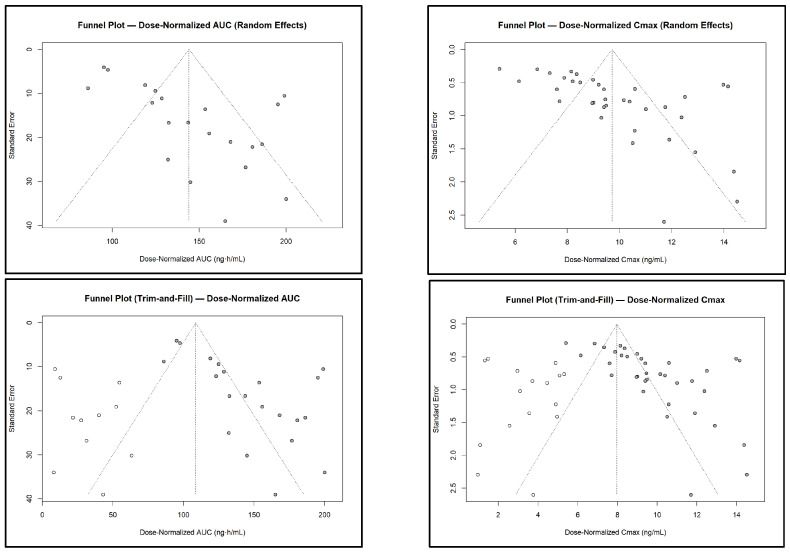
**Funnel plots for dose-normalized AUC and Cmax before and after trim-and-fill adjustment.** The random-effects funnel plots show visible asymmetry for both parameters, suggesting potential small-study effects. Trim-and-fill analyses imputed missing studies (open circles) to improve symmetry, but the adjusted distributions did not materially alter the overall conclusions. Egger’s test confirmed significant asymmetry for both AUC (t = 4.41, *p* = 0.0003; z = 2.93, *p* = 0.0034) and Cmax (t = 4.35, *p* = 0.0001; z = 4.12, *p* < 0.001).

Funnel plot analyses (Figure 16), Egger’s regression tests, and trim-and-fill procedures were performed for both AUC and Cmax. Visual inspection of the funnel plots revealed noticeable asymmetry for dose-normalized AUC and Cmax, suggesting the presence of small-study effects or potential publication bias. Egger’s regression confirmed significant asymmetry for AUC (t = 4.41, *p* = 0.0003; z = 2.93, *p* = 0.0034) and Cmax (t = 4.35, *p* = 0.0001; z = 4.12, *p* < 0.001). Although trim-and-fill analyses imputed several potentially missing studies to improve symmetry, the adjusted pooled estimates remained largely unchanged, indicating that these small-study effects did not materially influence the overall conclusions.

## 3. Discussion

This systematic review and meta-analysis provide a comprehensive quantitative synthesis of the pharmacokinetics of doxazosin across a broad range of populations, study designs, formulations, and analytical methods. The findings highlight substantial between-study variability in systemic exposure, yet also demonstrate the robustness of pooled estimates after accounting for dose differences, influential studies, and potential small-study effects.

The ultimate objective of this review was to extract, summarize, and analyze published data on the PK of doxazosin in both healthy and diseased populations. Out of the 25 included articles, 13 were based on healthy populations following the oral route, while 9 studies reported the PK in different diseased states, including hypertension, renal failure, and liver cirrhosis. The predominance of low-quality ratings based on the JADAD scale indicates that many included PK studies lacked features such as randomization and blinding, which are rarely applicable to this research design. Therefore, the pooled results should be interpreted with caution, as traditional trial-based quality metrics may underestimate the methodological soundness of PK investigations. In contrast, the CASP tool, which places greater emphasis on study validity, data reliability, and methodological transparency, was considered more suitable for evaluating the included studies. Consequently, our confidence in the synthesized findings was primarily based on the CASP assessments, while recognizing the inherent limitations of applying conventional clinical trial appraisal tools to PK data. Two distinct brands were found to be bioequivalent to each other, indicating that both brands can be used as a treatment option [27,31]. The combination product containing doxazosin and finasteride showed similar PK parameters (AUC_0–∞_, C_max_, t_½_) compared twith doxazosin administered alone, indicating that these two drugs can be administered concomitantly for better management of BPH in males. [31]. Another study has investigated the effect of circadian rhythm on PK parameters of doxazosin, in which mean T_max_ was prolonged with greater variability following evening administration. This may be attributable to changes in blood flow, decreased intestinal mobility, and slower gastric emptying [33].

Doxazosin treatment begins with a standard dosage of 1 mg per day, which is increased by 2 mg every 7 to 14 days, reaching a maximum of 16 mg per day for hypertension or 8 mg per day for BPH, until blood pressure, urinary flow rate, or BPH symptoms are managed effectively [14]. This regimen requires four titration steps to reach therapeutically effective doses to avoid first-dose side effects. In comparison with other alpha blockers (terazosin, prazosin, alfuzosin, tamsulosin), the pharmacokinetics of doxazosin have been reported to be advantageous by having a long elimination half-life, better bioavailability, minimal food interactions, and higher plasma protein binding, whose details are portrayed in Supplementary Table S8. The CR dosage form was found to have a more gradual absorption profile compared to the IR dosage form, as depicted by prolonged T_max_ and lower C_max_ (about one-third of the IR dosage form). Therefore, the CR tablet displayed advantages over the standard formulation, and initial drug therapy can be started at therapeutically effective doses, minimizing the need for dose titration. Moreover, the CR formulation maintains uniform plasma concentrations along with decreased fluctuation index at steady state, which potentially leads to more consistent disease control [13]. Another study conducted with a CR dosage form in a healthy Korean population reported higher C_max_ compared with Caucasians. This disparity was also observed in the case of 2 mg doxazosin standard tablets [13,29], where the C_max_ and AUC of Caucasian populations were determined to be 74% and 63% of those of Asian populations, such as the Thai population, respectively. This may be due to polymorphism; so, ethnic considerations might be taken into account with doxazosin administration [34].

In patients with alcoholic liver cirrhosis, the mean AUC_0–∞_ was increased by 43% when compared to healthy subjects, which may be due to the fact that the major elimination pathway of this drug is via oxidation in the liver. Moreover, the increased value of MRT (mean residence time) indicates that patients with hepatic impairment required a longer time (almost 50% longer) to remove the same proportion of drug [39]. Similar findings were reported with other drugs (nefazodone, teniloxazine) undergoing extensive liver metabolism, where AUC_0–∞_ was elevated in patients with hepatic impairment [52,53]. As supported by PK data from the doxazosin prescribing information and clinical pharmacology literature, it is extensively metabolized by the liver, primarily via the CYP3A4 enzyme system. In patients with hepatic impairment, reduced metabolic clearance can lead to increased exposure and elevated plasma concentrations, increasing the risk of adverse events such as orthostatic hypotension and syncope. Therefore, in cases of mild liver damage (Child–Pugh A), dose initiation should be conservative, typically starting at the lowest available dose with slow titration and close monitoring [3]. Moreover, owing to its metabolism by the CYP3A4 enzyme, its systemic exposure may increase or decrease with enzyme inhibitors and enzyme inducer drugs, respectively [54]. Allelic variants of CYP3A4, such as CYP3A4*22, were presented in a study showing decreased expression of liver enzymes, and CYP3A4*1B was found to be prevalent among the African population [55]. This may play an important role in inter-ethnic variations in exposure to doxazosin.

A study performed with single and chronic dosing in hypertensive patients reported higher AUC_0–∞_ and a prolonged t_½_ at steady state, which reflects significant accumulation of the drug and saturation of metabolic activity of the liver with long-term therapy [45]. Another study focusing on elderly hypertensive patients found a linear relationship between C_max_ and the administered dose, which suggests that doxazosin absorption in this specified age group is dose-independent [43]. In the case of renal impairment, AUC and t_½_ were not altered compared to healthy individuals, depicting that doxazosin provides a safe dosage regimen for renal patients with concomitant hypertension [40]. A previous study presented polymorphism in orsomucoids, and variants such as ORM1*1, ORM1*3, and ORM1*2 showed marked changes in doxazosin clearance among different populations [56]. This may be due to high plasma protein binding with alpha-1 acid glycoprotein. Two studies reported an increase in the volume of distribution for doxazosin with age [57] and in those who were suffering from obesity. Furthermore, P-glycoprotein (P-gp) restricts the absorption of its substrate drugs by actively pumping them from the intestine back into the bloodstream. However, the higher bioavailability of doxazosin, i.e., 65%, indicates that P-gp-mediated efflux has minimal impact on its absorption. A study suggested that doxazosin may act as an inhibitor of P-gp, preventing its efflux action on other drugs and potentially increasing the intracellular accumulation of P-gp substrates [1,58].

Doxazosin shows about 40% higher systemic drug exposure at a 2 mg oral dose in patients with Child–Pugh A. For dose optimization, start taking 1 mg of immediate-release (IR) doxazosin once daily and titrate it carefully by doubling the dosage every 2–4 weeks as tolerated. At baseline and after each titration step, monitor patients’ heart rate, blood pressure, orthostatic symptoms, and liver function tests in supine and standing positions. Avoid the use of doxazosin in Child–Pugh C hepatic impairment due to lack of clinical data and its predominant hepatic metabolism [59,60,61]. The efficacy and tolerability of doxazosin in Asian populations are similar to those found in Western cohorts; thus, no dose titration is required. Dosage optimization is only recommended for frail or hepatically impaired patients or when the drug is co-administered with CYP inhibitors [61,62,63]. The safety and efficacy profile of doxazosin in elderly population aged ≥ 65 years is comparable to that of younger adults. However, due to the increased risk of comorbidities and frequent declines in hepatic, renal, or cardiac function, dose selection for older patients should be careful, typically beginning at the lower end of the dosing range [61]. To reduce the risk of falls, start IR doxazosin at 0.5–1 mg once a day and titrate gradually every 2–4 weeks to the lowest effective maintenance dose [60]. During dose titration, regularly check orthostatic vital signs and review concomitant anti-hypertensives and other drugs that increase the risk of falls [64]. The CR tablets of doxazosin are mostly recommended for BPH, with a once-daily dose of 4 or 8 mg. A study depicted comparable efficacy and tolerability profile of the IR and CR formulation of doxazosin in hypertensive populations, but because of flexible titration up to 16 mg/day, the IR tablets are preferred in such patients [22,62]. For doxazosin, about a 25–30% increase in AUC may warrant dose modification and close monitoring of side effects. This is based on established clinical practice and regulatory bioequivalence limits, i.e., 80–125%, where changes >25% are typically significant for medications with safety issues [65].

The drug–drug interaction of doxazosin with nifedipine reported no discernible change in PK parameters (AUC, C_max_, t_½_) despite the fact that nifedipine was shown to enhance apparent liver blood flow. This is why these two drugs can be used in combination for effective control of hypertension [48]. The co-administration of MDMA and doxazosin caused a decrease in the C_max_ of MDMA’s metabolite (MDA), which may be due to competitive inhibition of CYP3A4 by doxazosin [49]. Doxazosin undergoes extensive hepatic metabolism [1], mainly via the CYP3A4 enzyme and, to a lesser extent, via CYP2C9 and CYP2D6 [2]. Given its widespread use in patients with multiple comorbidities, the potential for CYP3A4-mediated drug–drug interactions are clinically relevant. Co-administration with potent CYP3A4 inhibitors such as azole antifungals [3], macrolide antibiotics [4], or certain calcium channel blockers [5] may increase doxazosin exposure, while CYP3A4 inducers such as rifampicin or carbamazepine may reduce its plasma concentrations [6]. Additionally, although doxazosin exhibits high plasma protein binding (98–99%), in vitro data in human plasma indicate that it does not affect the protein binding of drugs (digoxin, warfarin, phenytoin, or indomethacin). This suggests a low potential for clinically significant displacement interactions. Despite these theoretical risks, no formal in vivo interaction studies have been reported, highlighting a critical research gap that necessitates further pharmacokinetic evaluation to ensure safe use in polypharmacy and elderly populations [2]. The details are presented in Table 1. Drug–food interaction should be considered equally important from patients’ perspective. Doxazosin can be taken with or without food as it has no clinically significant effect on its PK profile [66]. A study showed an 18% higher C_max_ of doxazosin CR tablet in the fed condition compared with the fasted state. Therefore, it was not considered a clinically significant difference, depicting that patients on doxazosin therapy do not need dose adjustment with regard to food intake [13]. No significant chronopharmacokinetic effect of the time of administration of doxazosin has been observed on its efficacy, safety, or PK profile [35].

Enantiomer-specific PK analysis of doxazosin revealed notable differences between the S-(+)- and R-(−)-enantiomers. Across two subjects, the S-(+)-enantiomer exhibited approximately two-fold higher exposure (AUC 175–195 ng·h/mL) compared with the R-(−)-enantiomer (AUC 78–86 ng·h/mL). Similarly, C_max_ was higher for the S-(+)-enantiomer (4.47–5.98 ng/mL) versus the R-(−)-enantiomer (2.37–2.91 ng/mL) [36]. Although both doxazosin enantiomers exhibit similar α_1_-adrenoceptor binding affinity, the S-(+)-enantiomer demonstrates higher plasma protein binding and slower metabolic clearance compared with the R-(−)-enantiomer. This stereoselectivity is consistent with our findings of approximately two-fold higher AUC for the S-(+)-enantiomer. These pharmacokinetic differences may influence the magnitude and duration of pharmacodynamic effects and could contribute to interindividual variability in therapeutic response. Despite the observed enantiomeric differences, doxazosin is marketed as a racemic mixture because both enantiomers contribute to its overall therapeutic effect, and clinical efficacy and safety have been established for the racemate.

The meta-analysis results, together with the dose-normalized subgroup and sensitivity analyses, provide important insights into the sources and magnitude of pharmacokinetic variability observed across studies. Dose normalization was a critical step in standardizing PK parameters across studies that used different doses. Even after normalization, considerable heterogeneity (I^2^ ≈ 90%) persisted, indicating that true pharmacokinetic variability—rather than dose differences alone—drives much of the observed dispersion.

Subgroup analyses supported this interpretation, revealing that clinical characteristics such as hypertension, chronic kidney disease, ESRD, and liver cirrhosis were associated with elevated dose-normalized AUCs compared with healthy subjects. These findings suggest reduced clearance or altered distribution in these populations, consistent with known physiological impairments in hepatic or renal function. In contrast, subgroup analyses by analytical method, study quality, and single-dose versus steady-state design showed no significant differences, indicating that methodological factors were not major contributors to heterogeneity. Formulation-based comparisons demonstrated significantly lower dose-normalized AUC with controlled-release (CR) compared to immediate-release (IR) products. This supports the expected modified absorption characteristics of CR formulations and reinforces the need to consider formulation-specific kinetics when interpreting exposure metrics or making clinical decisions. Sensitivity analyses strengthened the credibility of the pooled estimates. Leave-one-out diagnostics showed that the overall results were stable, with the pooled AUC changing minimally and heterogeneity improving only when three influential studies were removed. This pattern indicates that while certain studies contribute disproportionately to heterogeneity, they do not fundamentally alter the interpretation of the pooled effect. Baujat plots further confirmed that only a small subset of studies drove the majority of heterogeneity for both AUC and Cmax, supporting the robustness of the findings despite high overall variability. Publication bias assessments revealed notable funnel plot asymmetry, and Egger’s regression identified significant small-study effects for both AUC and Cmax. However, trim-and-fill procedures demonstrated that the imputed studies had little impact on pooled estimates, suggesting that although small-study effects are present, they do not materially bias the conclusions. This reinforces the reliability of the aggregated exposure metrics. Meta-regression provided additional insights. Body weight was found to significantly reduce dose-normalized Cmax, implying that heavier individuals may experience lower peak concentrations per unit dose. Conversely, age did not significantly influence either dose-normalized AUC or Cmax, suggesting that age alone is not a major determinant of exposure when accounting for dose. Taken together, these results underscore the complexity of doxazosin pharmacokinetics and the importance of considering population-specific factors—particularly renal and hepatic function—when interpreting PK data or optimizing dosing strategies. While dose-normalized pooled estimates offer valuable reference points, the substantial heterogeneity and identified contributors to PK variability indicate that individualized dosing decisions may require more detailed patient-level data or physiologically based modeling approaches. Future studies with standardized designs, harmonized reporting of PK parameters, and inclusion of special populations are needed to further refine exposure predictions and support model-informed precision dosing.

This review has incorporated all the articles being published from 1986 to 2024. To date, this is the first systematic review that consolidates and quantifies PK parameters (C_max_, T_max_, AUC, CL/F, and t_½_) of doxazosin across different studies, populations, and dosage forms (IR vs. CR). Furthermore, this study represents the first quantitative meta-analysis of PK parameters (AUC and C_max_) for doxazosin conducted exclusively in adult healthy volunteers. By restricting the analysis to a homogeneous population, we minimized clinical confounders and provided a robust benchmark for interpreting drug exposure under controlled physiological conditions. Moreover, this review addresses a critical gap in the literature by systematically assessing influencing factors on the absorption, distribution, metabolism, and elimination (ADME) profile of doxazosin such as age, chronobiology, stereoisomerism, different brands employed, comorbidities (renal or hepatic impairment, hypertension), and ethnicity. Furthermore, this review provides a comparative analysis of the PK profiles between individuals with hepatic impairment (Child–Pugh A) and healthy subjects, with a focus on alterations in drug exposure, metabolism, and oral clearance. Additionally, this systematic review elaborates on the safety profile of doxazosin in patients with renal impairment. Also, it highlights anti-hypertensive drugs that can be safely co-administered with doxazosin to minimize adverse interactions and enhance treatment efficacy. These subgroup analyses offer clinically relevant insights that are not accessible from individual studies alone. In addition, our study provides a detailed comparison between IR and CR formulations, analyzing their absorption kinetics and steady-state concentrations, which is especially valuable for optimizing therapeutic regimens. Moreover, this systematic review and meta-analysis identify significant gaps in the existing literature, such as the lack of PK data on patients with mild (Child–Pugh B) liver damage and drug interactions. Based on these findings, our work highlights the need for future studies specifically designed to evaluate potential drug–drug interactions. By summarizing the variability and influencing factors, our study supports more personalized and evidence-based dosing recommendations, particularly for patients at risk of altered metabolism or drug accumulation.

There are some limitations of this systematic review and meta-analysis that should be considered. The data availability restricted the meta-analysis primarily to AUC and C_max_. Another consideration is that we did not perform a sensitivity analysis to assess the influence of individual studies. Given the limited number of eligible studies, such an analysis would have reduced statistical robustness and potentially biased the overall interpretation. To address between-study variability, we relied on random-effects modeling, which provides more conservative estimates and mitigates the impact of study-level differences. In this study, the pooled estimates for AUC and C_max_ revealed moderate heterogeneity across studies, which compromised the precision of the pooled estimates and suggests that results should be viewed cautiously rather than as generally applicable values. The majority of studies had low methodological quality, which reduced the overall strength of the evidence. Additionally, the restriction to English-language publications may have introduced language and publication bias and excluded crucial data published in other languages. The articles on drug interactions were limited to anti-hypertensive drugs, so it was not possible to draw robust conclusions regarding other major drug classes. Most of the included studies focus on the healthy population, and the literature we identified has limited data available for special populations, including geriatric, pediatric, and patients with moderate to severe hepatic impairment. Moreover, despite doxazosin’s established use in the treatment of BPH, there is a notable lack of PK data specific to this population, limiting the ability to optimize dosing and assess potential drug interactions in routine urological practice. The clinical studies included in this review employed a variety of analytical techniques. Older studies employed outdated methods with lower sensitivity and specificity than newer LC-MS/MS approaches. These assay variations and varied lower limits of quantification contribute to inter-studies heterogeneity in reported AUC and C_max_. Most of the included studies were controlled single-dose trials including healthy individuals, which differs from real-world chronic use of doxazosin in patients with comorbidities. Single-dose data cannot account for steady-state accumulation, adherence variability, or drug–drug interactions that occur in real-world settings. Limited data from two subjects suggest that stereoselective metabolism, potentially mediated by CYP3A4 and other hepatic enzymes, could contribute to interindividual or ethnic differences in exposure and response. These findings highlight a critical research gap, underscoring the need for larger-scale, enantiomer-specific pharmacokinetic and pharmacodynamic studies to better understand their impact on therapeutic outcomes.

## 4. Materials and Methods

### 4.1. Study Design

This systematic search was conducted following the Cochrane Handbook guidelines [67] and is reported in accordance with the Preferred Reporting Items for Systematic Reviews and Meta-Analyses (PRISMAs) [68]. This systematic review is registered in PROSPERO with ID CRD42024610494, and the checklist is shown in Supplementary Table S6. In addition, a meta-analysis of key PK parameters was performed to synthesize the data across eligible studies quantitatively.

### 4.2. Literature Search Strategy

**Figure 17:**
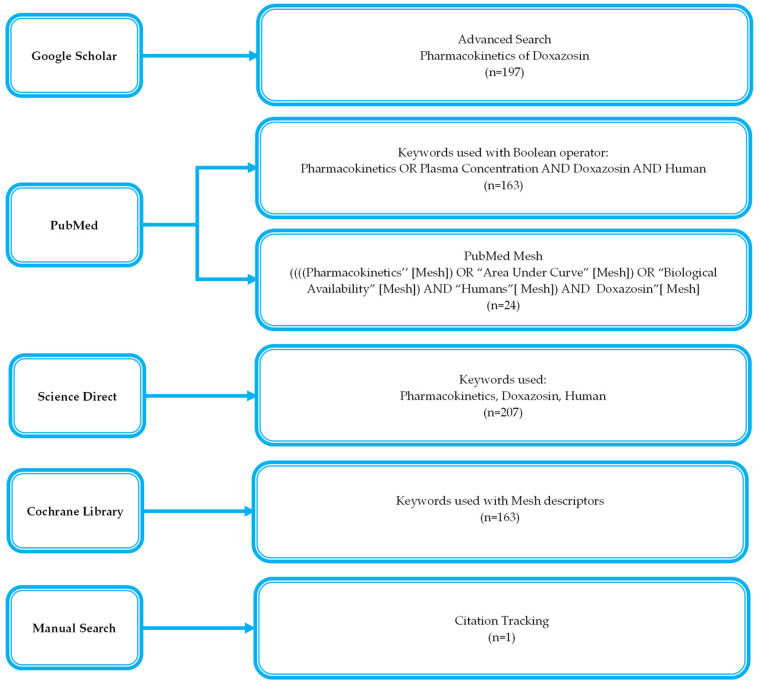
Flow diagram of search strategy.

The literature for this review was searched thoroughly, encompassing four databases (Google Scholar, PubMed, ScienceDirect, and Cochrane Library) till December 2024. The appropriate keywords, such as “doxazosin” and “pharmacokinetics”, were used to extract relevant articles. The adopted search strategies are shown in Figure 17.

### 4.3. Eligibility Criteria

The original research articles having plasma concentration–time profiles being reported after administration of IR and CR dosage forms to healthy populations or those with liver, kidney, and cardiac diseases were considered eligible to be included in this review. Additionally, studies on drug–drug and drug–food interaction were also included. These inclusions were carried out regardless of study protocol, dosing schedules, and dose formulation. The year of publication was not restricted, and only English-language papers were retrieved.

### 4.4. Study Selection and Data Extraction Process

Following a comprehensive search with the relevant keywords being used across pre-specified databases, all the articles were imported into EndNote version 20 to identify duplicates. The articles were then screened by their title and abstract; animal-based and limited-access articles were excluded. Editorial letters, conference papers, commentary, and brief reviews were not included. Screening details are presented in Supplementary Table S1. Finally, after full-text screening of articles that met eligibility requirements, all relevant studies were selected. Data pertaining to demographic characteristics of selected studies, including age, number of participants, and ethnicity, were extracted. Moreover, frequency of drug administration, dosing range, dosage form, and analytical method applied to obtain lasma concentrations were also included. The units of PK parameters, i.e., AUC (ng·h/mL), C_max_ (ng/mL), and CL/F (mL/min/kg), were converted to the most commonly employed units to ensure uniformity.

### 4.5. Quality Assessment

The included studies were assessed for quality by applying the JADAD scoring tool, which uses five questions to rank the articles based on withdrawal of participants, blinding, and randomization, as represented in Supplementary Table S2. Studies with scores greater than 4, between 3 and 4, and less than 3 were considered as high, moderate, and low quality, respectively [69]. The Critical Appraisal Skills Program (CASP) [70] and the Clinical Pharmacokinetic Critical Appraisal (CACPK) [71] quality assessment tools were also used to check the validity of studies and are presented in Supplementary Table S3 and Supplementary Table S4, respectively. CACPK scoring criteria include 21 questions, and studies were indicated as high, moderate, and low quality with scores > 13, 12 to 13, and less than 12, respectively. The CASP tool ranked articles as low, moderate, and high quality with scores less than 4, 4 to 6, and >6, respectively. Articles were also evaluated with the Cochrane Collaboration Tool (CCT) to check the risk of bias [72], as presented in Supplementary Table S5. According to CCT scoring criteria, studies scoring more than 4 were at low risk of bias, whereas studies having a score of 3–4 and less than 3 were at moderate and high risk of bias, respectively. The JADAD scale emphasizes randomization, blinding, and withdrawal reporting, criteria generally unsuitable for pharmacokinetic (PK) studies, which are often open-label and non-randomized by design. The CASP checklist, however, focuses on study validity, data collection, clarity of objectives, and relevance to research aims, factors which are more aligned with PK study methodology.

### 4.6. Statistical Analysis

The meta-analysis was conducted using R version 4.4.1 employing the metafor package version 4.8-0 [73]. All reported AUC and Cmax values were converted to dose-normalized pharmacokinetic parameters to enable standardized comparison across studies using different doses and formulations. A random-effects model (REML) was applied to estimate pooled dose-normalized AUC and Cmax while accounting for between-study variability. Statistical heterogeneity was quantified using the I^2^ statistic, and forest plots were generated to summarize individual study effects and pooled estimates. Studies were included in the quantitative analysis only if they provided sufficient data—namely sample size, mean, and standard deviation (SD). For studies reporting standard error (SE) instead of SD, conversion was performed using the following formula:

**SD = SE × √n**,

and calculations were verified using the Meta-Analysis Accelerator tool [74]. Studies lacking essential quantitative data were excluded from the meta-analysis and instead described narratively.

A comprehensive suite of sensitivity analyses was performed. Leave-one-out diagnostics assessed the influence of individual studies, and Baujat plots identified studies contributing most to heterogeneity and overall effect-size influence. Assumptions of the random-effects model—between-study heterogeneity and approximate normal distribution of true effects—were evaluated through visual inspection of residuals, influence diagnostics, and comparison of τ^2^ estimators. Extreme values and potential outliers were examined using influence analysis, and identified outlier studies were addressed as described in the Results section.

To assess the presence of small-study effects and publication bias, funnel plots, trim-and-fill analyses, and Egger’s regression test were applied. Additionally, meta-regression was used to examine the influence of demographic covariates (age and body weight) on AUC and Cmax. Statistical significance was defined as *p* < 0.05.

## 5. Conclusions

This systematic review and meta-analysis provide an updated understanding of doxazosin pharmacokinetics across healthy and diseased populations. Higher exposure was observed in patients with renal dysfunction and liver cirrhosis, indicating reduced clearance and the need for closer monitoring in these groups. Immediate-release formulations produced greater exposure than controlled-release products, and a trend toward higher exposure in Asian compared with non-Asian populations was observed, although this difference was not statistically significant after dose normalization. Despite evidence of small-study effects, pooled estimates remained robust across sensitivity analyses. These findings highlight meaningful population and formulation differences that should be considered when individualizing doxazosin therapy and underscore the need for more harmonized PK study designs to strengthen future evidence and support model-informed dosing strategies.

## Figures and Tables

**Table 3:** PK parameters of doxazosin in diseased population.

Patient Characteristics	Dose (mg)	AUC_0–inf_ (ng·h/mL)	C_max_ (ng/mL)	T_max_ (h)	t_½_ (h)	Others	Refs.
Group 1	1	132 (25) ^c^	7.6 (0.6)	3.6 (0.6)	12.6 (3.3)	NR	[40]
Group 2		168 (21)	9.4 (0.6)	2.6 (0.3)	15.5 (1.7)	NR	
Group 3		165 (39)	11.7 (2.6)	2.6 (0.4)	10.5 (1.5)	NR	
Group 4		NR	NR	NR	NR	NR	
Hypertensive	1	NR	14.3	2	11.9	C p (ng/mL) after 24 h: 2.8	[41]
	4		42.3 (4.9)	2.3 (0.7)	12.8 (1.2)	C p (ng/mL) after 24 h: 12.5 (2.2)	
	8		65.5	2	8.3	C p (ng/mL) after 24 h: 10.3	
	16		151.7 (13.5)	2.3 (0.3)	11.6 (0.9)	C p (ng/mL) after 24 h: 38.8 (7.3)	
Hypertensive	SD: 2	182.0 (13.6) *	18.9 (1.5)	1.4 (0.2)	11.1 (1.1)	NR	[42]
	MD: 2	273.0 (30.2) *	25.8 (3.1)	1.5 (0.2)	12.9 (0.8)	NR	
Hypertensive	1	NR	9.4 (1.5)	Median (Range) 2.0 (2–4)	14.4 (1.7)	C p (ng/mL) after 24 h: 3.2 (1.0)	[43]
	2		18.0 (3.2)	2.5 (1–3)	15.7 (3.0)	C p (ng/mL) after 24 h: 6.7 (1.3)	
	4		28.7	1.5	18.1	C p (ng/mL) after 24 h: 11	
	8		84.7 (10.6)	3 (2–3)	16.2 (7.5)	C p (ng/mL) after 24 h: 26.9 (3.5)	
	16		168 (45.2)	3.5 (2–4)	16.9 (6.7)	C p (ng/mL) after 24 h: 57.8 (26.9)	
Hypertensive	Group 1: 1*8 mg	1445 (469)	115 (39)	2.1 (0.9)	21.7	NR	[44]
	Group 1: 2*4 mg	1414 (525)	116 (45)	1.8 (0.9)	21.7	NR	
	Group 2: 4*2 mg	1160 (216)	100 (14)	1.5 (0.7)	22.5	NR	
	Group 2: 8*1 mg	1131 (214)	99 (20)	1.9 (0.8)	22.5	NR	
Hypertensive	SD: 2	287.2 (104.8)	18.6 (6.5)	3.2 (1.8)	8.8 (2.3)	NR	[45]
	At 1 Week: 2	372.6 (136.3)	17.9 (5.1)	3.2 (1.9)	12.5 (3.3)	NR	
	At 6 Weeks: 2	369.4 (133.2)	18.9 (6.4)	2.4 (1.5)	12.3 (2.5)	NR	
Hypertensive	SD: 1	200 (34)	15.7 (2.7)	2.1 (0.4)	10.7 (1.2)	NR	[46]
	MD: 1	187 (18) *	19.3 (2.3)	1.9 (0.2)	11.5 (1.1)	NR	
	MD: 2	464 (44) *	41.6 (3.1)	1.7 (0.2)	12.6 (1.1)	NR	
	MD: 4	833 (131) *	79.4 (10.5)	2.0 (0.3)	11.4 (0.9)	NR	
	MD: 8	1000 (40) *	100.7 (13.8)	2.7 (0.4)	10.8 (0.5)	NR	
Hypertensive	SD: 1	162 (59)	9.7 (3.4)	2.5 (1.4)	12.9 (5.1)	MRT (h): 19.3 (7.6)	[47]
	MD: 16	268 (129) ^a^	13.6 (4.7) ^b^	2.9 (1.3)	15.0 (3.4)	MRT (h): 20.9 (5.1)	
Healthy	2	172 (61.0)	12.3 (3.3)	3.0 (1.0)	22 (7)	CL/F (ml/min): 214 (67.6), MRT (h): 4 (4)	[39]
Liver Cirrhosis	2	246 (84.0)	10.8 (2.0)	4.0 (1.0)	24 (9)	CL/F (mL/min): 149 (45.0), MRT (h): 21 (5)	

Data presented as mean (standard deviation); AUC_0–inf_: area under plasma concentration curve from 0 to infinity; C_max_: peak plasma concentration; C p: plasma concentration; CL/F: apparent oral clearance; MD: maintenance dose; MRT: mean residence time; NR: not reported; Refs: references; SD: single dose; T_max_: time to reach peak plasma concentration; t_½_: elimination half-life; Group 1 (healthy, normotensive); Group 2 (renally impaired but not on hemodialysis); Group 3 (with end-stage renal failure studied between hemodialysis; Group 4 (with end-stage renal failure studied during hemodialysis); ^a^ AUC in the mentioned study is presented as AUC/dose; ^b^ C_max_ is expressed as C_max_/dose. In the reference study, C_max_ was divided by the final dose for each individual, and the mean C_max_ was reported as the final chronic dose varied among individuals with a median (range) value of 8 mg (1–16 mg); ^c^ AUC values in the presented study are normalized for body weight; * AUC given as AUC_0–24_.

**Table 4:** PK parameters of drug–drug and drug–food interactions of doxazosin.

Drug	Dose (mg)	AUC (ng·h/mL)	C_max_ (ng/mL)	T_max_ (h)	CL/F (mL/min)	Refs.
**Drug–Drug Interaction**						
Dox + ENL	Dox = 1, ENL = 10					[50]
	Dox (Fd)	NR	7.7 (2.7)	2.5 (1.3)	185 (2.52)	
	Dox (ss)	NR	11.9 (4.7)	2.3 (0.3)	161.667 (2.23)	
	Dox (ss) + ENL(Fd)	NR	10.7 (3.3)	2.8 (1.1)	153.33 (1.99)	
	Dox (ss) + ENL(ss)	NR	9.9 (3.0)	2.5 (0.7)	176.667 (2.67)	
Dox + Nif	Dox = 2, Nif = 20					[48]
	Dox (Fd)	257 (77)	16.4 (3.3)	3.2 (2.9)	NR	
	Dox (ss)	307 (94)	23.5 (6.0)	2.8 (0.8)		
	Dox (ss) + Nif (Fd)	301 (81)	24.7 (5.0)	2.0 (1.1)		
	Dox (ss) + Nif (ss)	256 (49)	20.2 (3.2)	3.8 (2.4)		
Dox + MDMA	Dox = 8, MDMA = 125					[49]
	Placebo + MDMA	AUC_0–6_ 1029 (49)	247 (12)	2.5 (0.3)	NR	
	Dox + MDMA	AUC_0–6_ 992 (50)	243 (12)	2.9 (0.2)		
	MDA					
	Placebo + MDMA	AUC_0–6_ 51.9 (4.6)	14.0 (1.4)	5.7 (0.2)		
	Dox + MDMA	AUC_0–6_ 44.3 (3.2)	12.3 (1.1)	5.4 (0.3)		
	HMMA Metabolite					
	Placebo + MDMA	AUC_0–6_ 717 (97)	168 (22)	1.9 (0.2)	NR	
	Dox + MDMA	AUC_0–6_ 730 (104)	169 (25)	2.1 (0.2)		
**Drug–Food Interaction**						
Dox	CR 8 mg (fasted)	569 (187)	18.3 (5.9)	14 (4)	NR	[13]
	CR 8 mg (fed)	670 (234)	24 (7.1)	11 (2)		
	STD 2 mg (fasted)	195 (45)	13.7 (2.9)	2 (0.8)		

Data presented as mean (standard deviation); AUC: area under plasma concentration–time curve; C_max_: peak plasma concentration; CR: controlled-release formulation; CL/F: oral clearance; Dox: doxazosin; ENL: enalapril; Fd: first dose; HMMA: 4-hydroxy-3-methoxymethamphetamine; MDMA: 3,4-methylenedioxymethamphetamine; MDA: 3,4-methylenedioxyamphetamine; Nif: nifedipine; NR: not reported; Refs: references; ss: steady-state; STD: standard formulation; T_max_: time to reach C_max_.

## Data Availability

All the data used for this publication are either presented in the main article or are available as Supplementary Materials.

## Supplementary Materials

The following supporting information can be downloaded at: https://www.mdpi.com/article/10.3390/ph18121825/s1, Table S1: Screening of articles based on title, abstract, animal-based, no access, language barrier, and full text; Table S2: Quality assessment of included articles based on JADAD scoring; Table S3: Quality assessment of included articles based on Critical Appraisal Skills Program (CASP) scoring; Table S4: Quality assessment of included articles based on Critical Appraisal Clinical Pharmacokinetics Tool (CACPK) scoring; Table S5: Assessment of risk of bias based on Cochrane Collaboration Tool (CCT); Table S6: Prisma checklist 2020; Table S7: Demographic characteristics of doxazosin studies; Table S8: Comparison of pharmacokinetic parameters of doxazosin with other alpha blockers; Table S9: Clinical dosing and monitoring recommendation of doxazosin; Table S10: Drug-drug interaction of doxazosin with CYP3A4 inhibitor and inducer drugs; Figure S1: Forest plot of dose-normalized Cmax stratified by population; Figure S2: Forest plot of dose-normalized Cmax stratified by formulation type; Figure S3: Forest plot of dose-normalized Cmax stratified by study design; Figure S4: Forest plot of dose-normalized Cmax stratified by analytical method; Figure S5: Forest plot of dose-normalized Cmax stratified by ethnicity; Figure S6: Leave-one-out sensitivity analysis for Cmax; Figure S7: Baujat plot for Cmax illustrating each study’s contribution to heterogeneity and influence on the pooled mean [13,27,28,29,30,31,33,34,35,36,37,38,39,40,41,42,43,44,45,46,47,49,50,51,59,60,61,63,64,75,76,77,78,79,80,81,82].
