# Supplementary material for: A Systematic Review and Meta-Analysis of Doxazosin Pharmacokinetics in Healthy and Diseased Populations
Source: Pharmaceuticals (Basel). 2025 Nov 29;18(12):1825. doi: 10.3390/ph18121825 (PMC12735885; doi:10.3390/ph18121825)
Supplement: Supplementary file 1 [file pharmaceuticals-18-01825-s001.zip › pharmaceuticals-3832843-supplementary.pdf]

A Systematic Review and Meta-Analysis of Doxazosin Pharmacokinetics in Healthy and Diseased Populations

Dania Fatima [1†], Mohammed S. Alasmari [2†], Yousef Alshomrani [2], Ammara Zamir [1], Faleh Alqahtani [3*], Iltaf Hussain [4], Muhammad Fawad Rasool [1*]

[1] Department of Pharmacy Practice, Faculty of Pharmacy, Bahauddin Zakariya University, 60800, Multan, Pakistan; daniafatima14@gmail.com (D.F); ammarazamir20@gmail.com (A.Z); fawadrasool@bzu.edu.pk (M.F.R)

[2] Security Forces Hospital Program, General Directorate of Medical Services, Ministry of Interior, Riyadh, SaudiArabia; aljammaz7010@gmail.com (M.S.A); Dr.yousef11111@gmail.com (Y.A)

[3] Department of Pharmacology and Toxicology, College of Pharmacy, King Saud University, Riyadh 11451, Saudi Arabia; afaleh@ksu.edu.sa (F.A)

[4] Center for Drug Safety and Policy, Xi'an Jiatong University, Xi'an PR China; iltafhussain@stu.xjtu.edu.cn (I.H)

*Corresponding authors: fawadrasool@bzu.edu.pk (M.F.R.); afaleh@ksu.edu.sa (F.A.)

Telephone: +92 3008639046

† These authors contributed equally to this work.

Supplementary Table S1. Screening of articles based on title, abstract, animal-based, no access, language barrier, and full text.

Sr no.	Title	Reason
1	(1991). "The treatment of mild hypertension study. A randomized, placebo-controlled trial of a nutritional-hygienic regimen along with various drug monotherapies. The Treatment of Mild Hypertension Research Group." Archives of internal medicine 151(7): 1413-1423.	Title
2	(1994). "Combined effect of a low fat diet and doxazosin on blood pressure control and blood lipids. Hunter Hypertension Research Group." J Hum Hypertens 8(12): 907-910.	Title
3	(2004). "Guideline 1: goals of antihypertensive therapy in CKD." American Journal of Kidney Diseases 43: 65-230.	Title
4	Abellán, J., F. H. Menárquez, A. Molina and J. M. Ferrer (1993). "Assessment of the antihypertensive effectiveness and variation of the metabolic profile with doxazosine versus verapamil in the HBP." Hipertensión 10(Extra): 66-.	Title
5	Agabiti-Rosei, E., M. L. Muiesan, D. Rizzoni, R. Zulli, S. Calebich, M. Beschi, M. Castellano and G. Muiesan (1992). "Reduction of left ventricular hypertrophy after longterm antihypertensive treatment with doxazosin." J Hum Hypertens 6(1): 9-15.	Title
6	Aguilar-Hernández, G., B. A. López-Romero, M. Nicolás-García, Y. Nolasco-González, H. S. García-Galindo and E. Montalvo-González (2023). "Nanosuspensions as carriers of active ingredients: Chemical composition, development methods, and their biological activities." Food Research International 174: 113583.	Title
7	Ahaneku, J. E., G. O. Taylor, E. O. Agbedana, O. Walker and L. A. Salako (1994). "Changes in lipid and lipoprotein values during a cross-over treatment of doxazosin, moduretic and amlodipine in hypertensive patients." JPMA. The Journal of the Pakistan Medical Association 44(7): 166-169.	Title
8	Ahaneku, J. E., O. G. Taylor, D. Walker, O. E. Agbedana and L. A. Salako (1994). "Blood pressure and biochemical changes during doxazosin monotherapy in Nigerian hypertensive patients." Current therapeutic research 55(9): 1067-1074.	Title
9	Akduman, B. and E. D. Crawford (2001). "Terazosin, doxazosin, and prazosin: current clinical experience." Urology 58(6, Supplement 1): 49-54.	Abstract
10	Al Zahraa, G., N. G. Eissa, H. M. El Nahas and G. F. Balata (2021). "Fast disintegrating tablet of Doxazosin Mesylate nanosuspension: Preparation and characterization." Journal of Drug Delivery Science and Technology 61: 102210.	Full text
11	Alarayyed, N. A., B. R. Graham, B. N. Prichard and C. C. Smith (1995). "The potentiation of adrenaline-induced in vitro platelet aggregation by ADP, collagen and	Title

Sr no.	Title	Reason
	serotonin and its inhibition by naftopidil and doxazosin in normal human subjects." Br J Clin Pharmacol 39(4): 369-374.	
12	Al-Kubati, S. S. F. S., M. Al-Ghazawi and M. F. Tutunji (2006). Bioequivalence Evaluation of a Generic Product of Doxazosin Compared to the Reference Product Cardura®, University of Jordan.	Language
13	Al-Tamimi, D., M. Alani, A. Ammoo and J. Ibraheem (2020). "Linear pharmacokinetics of doxazosin in healthy subjects." Int J Res Pharm. Sci 11(1): 1031-1039.	Full text
14	Al-Tamimi, D., K. Al-Kinani, S. Taher and A. Hussein (2022). "Effect of Food on the Pharmacokinetics of Fluoxetine in Healthy Male Adult Volunteers (Conference Paper)." Iraqi Journal of Pharmaceutical Sciences (P-ISSN 1683-3597 E-ISSN 2521-3512) 31(Suppl.): 153-161.	Title
15	Al-Tamimi, D. J. J., M. I. A. Al-Mahroos, M. J. J. Al-Tamimi and J. J. Ibraheem (2022). "Pharmacokinetic comparison and bioequivalence evaluation between a newly formulated generic and the brand cefuroxime axetil tablets in healthy male adult fasting subjects." Research Journal of Pharmacy and Technology 15(5): 2184-2192.	Title
16	Altıokka, G. (2014). Doksazosinin Elektroanalitik Ve Bazı Yöntemlerle Miktar Tayini, Anadolu University (Turkey).	Language
17	Ames, R. P., S. G. Chrysant, F. Gonzalez, H. W. Schnaper, S. Spann and M. T. Velasquez (1989). "Effectiveness of doxazosin in systemic hypertension." American journal of cardiology 64(3): 203-208.	Abstract
18	Ammoo, A. M., D. J. J. Al-Tamimi, M. I. A. Al-Mahroos, M. J. J. Al-Tamimi and J. J. Ibraheem (2021). "Pharmacokinetics of Fluconazole tablets administered to healthy subjects." Journal of Advanced Pharmacy Education & Research| Apr-Jun 11(2): 93.	Title
19	Andersen, M., C. Dahlstrand and K. Høye (2000). "Double-blind trial of the efficacy and tolerability of doxazosin in the gastrointestinal therapeutic system, doxazosin standard, and placebo in patients with benign prostatic hyperplasia." Eur Urol 38(4): 400-409.	Abstract
20	Andersen, P., I. Seljeflot, A. Herzog, H. Arnesen, I. Hjermann and I. Holme (1998). "Effects of doxazosin and atenolol on atherothrombogenic risk profile in hypertensive middle-aged men." Journal of cardiovascular pharmacology 31(5): 677-683.	Abstract
21	Anderson, J. R. and J. J. Nawarskas (2001). "CARDIOVASCULAR DRUG-DRUG INTERACTIONS." Cardiology Clinics 19(2): 215-234.	Title
22	Andersson, P. E., J. Johansson, C. Berne and H. Lithell (1994). "Effects of selective alfa 1 and beta 1-adrenoreceptor blockade on lipoprotein and carbohydrate metabolism in hypertensive subjects, with special emphasis on insulin sensitivity." Journal of human hypertension 8(3): 219-226.	Title

Sr no.	Title	Reason
23	Andersson, P. E. and H. Lithell (1996). "Metabolic effects of doxazosin and enalapril in hypertriglyceridemic, hypertensive men. Relationship to changes in skeletal muscle blood flow." American journal of hypertension 9(4 Pt 1): 323-333.	Abstract
24	Andriole, G. L. and R. Kirby (2003). "Safety and Tolerability of the Dual 5α-Reductase Inhibitor Dutasteride in the Treatment of Benign Prostatic Hyperplasia." European Urology 44(1): 82-88.	Title
25	Anegón, M., J. Esteban, R. Jiménez-García, V. S. de Burgoa, J. Martínez and A. G. de Miguel (2002). "A postmarketing, open-label study to evaluate the tolerability and effectiveness of replacing standard-formulation doxazosin with doxazosin in the gastrointestinal therapeutic system formulation in adult patients with hypertension." Clinical therapeutics 24(5): 786-797.	Title
26	Angus, J. A. and C. E. Wright (2016). "Novel α1-adrenoceptor antagonism by the fluroquinolone antibiotic trovafloxacin." European Journal of Pharmacology 791: 179-184.	Title
27	Arakawa, K. (1994). "Effects of BRL38227 on Hypertension, Serum Lipid and Sugar Metabolism in Patients with Essential Hypertension: double-blind, Group-comparison Study with Doxazosin Mesilate." Yakuri to chiryo (japanese pharmacology and therapeutics) 22(6): 2841-2870.	Title
28	Aranda, P., F. J. Aranda, M. Frutos, J. L. Pizarro and E. Lopez de Novales (1996). "Antihypertensive and metabolic effects of doxazosin in the hypercholesterolemic hypertensive postmenopausal women: a double blind study." American journal of hypertension 9(4 Suppl 1): 171A.	Abstract
29	Araujo, F., J. Mateo de Castro and C. Encinas Barrios (2010). "[Ezetimibe: caution in the face of doubt]." Farm Hosp 34(4): 212-214.	Language
30	Aronow, W. S., J. L. Fleg, C. J. Pepine, N. T. Artinian, G. Bakris, A. S. Brown, K. C. Ferdinand, M. Ann Forciea, W. H. Frishman, C. Jaigobin, J. B. Kostis, G. Mancia, S. Oparil, E. Ortiz, E. Reisin, M. W. Rich, D. D. Schocken, M. A. Weber, D. J. Wesley, R. A. Harrington, E. R. Bates, D. L. Bhatt, C. R. Bridges, M. J. Eisenberg, V. A. Ferrari, J. D. Fisher, T. J. Gardner, F. Gentile, M. F. Gilson, M. A. Hlatky, A. K. Jacobs, S. Kaul, D. J. Moliterno, D. Mukherjee, R. S. Rosenson, J. H. Stein, H. H. Weitz and D. J. Wesley (2011). "ACCF/AHA 2011 Expert Consensus Document on Hypertension in the Elderly: A Report of the American College of Cardiology Foundation Task Force on Clinical Expert Consensus Documents Developed in Collaboration With the American Academy of Neurology, American Geriatrics Society, American Society for Preventive Cardiology, American Society of Hypertension, American Society of Nephrology,	Title

Sr no.	Title	Reason
	Association of Black Cardiologists, and European Society of Hypertension." Journal of the American Society of Hypertension 5(4): 259-352.	
31	as contraindicações do Doxuran, Q. "Bula do Doxuran Doxuran, para o que é indicado e para o que serve?".	Language
32	Assis, A. M. d. (2021). Efeito da embolização das artérias prostáticas no componente dinâmico da hiperplasia prostática benigna: achados de elastografia por ultrassonografia, Universidade de São Paulo.	Language
33	Babamoto, K. S. and W. T. Hirokawa (1992). "Doxazosin: a new alpha 1-adrenergic antagonist." Clin Pharm 11(5): 415-427.	Review
34	Bachmann, K., L. Jauregui, R. Chandra and K. Thakker (2003). "Influence of a 3-day regimen of azithromycin on the disposition kinetics of cyclosporine A in stable renal transplant patients." Pharmacological Research 47(6): 549-554.	Title
35	Back, S. (2021). "Doxazosin in the treatment of co-occurring alcohol use disorder and posttraumatic stress disorder among veterans: a randomized clinical trial." Neuropsychopharmacology 46: 20.	Title
36	Bączek, T. and R. Kaliszan (2001). "Quantitative structure/retention relationships in affinity chromatography." Journal of Biochemical and Biophysical Methods 49(1): 83-98.	Title
37	Baek, M.-J., H.-J. Shin, J.-H. Park, J. Kim, I.-M. Kang, J. I. Lee and D.-D. Kim (2022). "Preparation and evaluation of the doxazosin-bentonite composite as a pH-dependent controlled-release oral formulation." Applied Clay Science 229: 106677.	Full text
38	Bailey, R. R., P. L. Nairn and R. J. Walker (1986). "Effect of doxazosin on blood pressure and renal haemodynamics of hypertensive patients with renal failure." N Z Med J 99(815): 942-945.	Abstract
39	Bartels, A. C. C., P. M. J. M. de Vries, L. P. Oe, H. van Bronswÿk, A. J. M. Donker, R.-J. Réveillaud, J.-P. Fillastre and P. Zech (1988). "Doxazosin in the treatment of patients with mild or moderate hypertension and mild or moderate renal insufficiency." American Heart Journal 116(6, Part 2): 1772-1777.	Abstract
40	Bello, M. L., A. M. Junior, C. A. Freitas, M. L. A. Moreira, J. P. da Costa, M. A. de Souza, B. A. M. C. Santos, V. P. de Sousa, H. C. Castro, C. R. Rodrigues and L. M. Cabral (2022). "Development of novel montmorillonite-based sustained release system for oral bromopride delivery." European Journal of Pharmaceutical Sciences 175: 106222.	Title

Sr no.	Title	Reason
41	Bernieh, D., G. Lawson and S. Tanna (2017). "Quantitative LC–HRMS determination of selected cardiovascular drugs, in dried blood spots, as an indicator of adherence to medication." Journal of Pharmaceutical and Biomedical Analysis 142: 232-243.	Title
42	Biswas, N., A. Guha, R. K. Sahoo and K. Kuotsu (2015). "Pulse release of doxazosin from hydroxyethylcellulose compression coated tablet: mechanistic and in vivo study." Int J Biol Macromol 72: 537-543.	Full text
43	Black, H. R. (2003). "Doxazosin as combination therapy for patients with stage 1 and stage 2 hypertension." Journal of cardiovascular pharmacology 41(6): 866-869.	Review
44	Blanco, M. A., J. E. Hernández, R. Jiménez-García, V. S. de Burgoa, J. M. González, G. H. Herrero and A. G. de Miguel (2001). "Estudio de farmacovigilancia para evaluar la seguridad y efectividad de doxazosina GITS en pacientes hipertensos que recibían tratamiento previo con doxazosina estándar." Hipertensión y Riesgo Vascular 18(9): 411-417.	Language
45	Bock, M. G. and M. A. Patane (2000). Chapter 20. Toward the development of α1a adrenergic receptor antagonists. Annual Reports in Medicinal Chemistry, Academic Press. 35: 221-230.	Title
46	Boero, R., C. Rollino, C. Massara, I. M. Berto, P. Perosa, G. Vagelli, G. Lanfranco and F. Quarello (2003). "The verapamil versus amlodipine in nondiabetic nephropathies treated with trandolapril (VVANNTT) study." American journal of kidney diseases 42(1): 67-75.	Title
47	Bramer, S. L., W. P. Forbes and S. Mallikaarjun (1999). "Cilostazol pharmacokinetics after single and multiple oral doses in healthy males and patients with intermittent claudication resulting from peripheral arterial disease." Clinical pharmacokinetics 37 Suppl 2: 1-11.	Title
48	Brieke, A., D. P. Kao, R. Zolty, E. M. Gilbert, A. D. Robertson, M. R. Bristow and B. D. Lowes (2011). "Transcriptional profiles of ventricular ectopy on b-blocker therapy." Journal of cardiac failure 17(8): S35-S36.	Title
49	Broadbent, A. L., R. J. Fell, S. L. Codd, K. A. Lightley, S. Konagurthu, D. G. Koehler-King and J. D. Seymour (2010). "Magnetic resonance imaging and relaxometry to study water transport mechanisms in a commercially available gastrointestinal therapeutic system (GITS) tablet." International journal of pharmaceutics 397(1-2): 27-35.	Title
50	Brown, M. J. and J. E. Dickerson (1995). "Alpha-blockade and calcium antagonism: an effective and well-tolerated combination for the treatment of resistant hypertension." J Hypertens 13(6): 701-707.	Title
51	Bruynzeel, H., R. A. Feelders, T. H. Groenland, A. H. van den Meiracker, C. H. van Eijck, J. F. Lange, W. W. de Herder and G. Kazemier (2010). "Risk Factors for	Title

Sr no.	Title	Reason
	Hemodynamic Instability during Surgery for Pheochromocytoma." J Clin Endocrinol Metab 95(2): 678-685.	
52	Bryniarski, P., K. Nazimek and J. Marcinkiewicz (2022). "Immunomodulatory properties of antihypertensive drugs and digitalis glycosides." Expert Rev Cardiovasc Ther 20(2): 111-121.	Title
53	Bull, R. J., J. Crook, M. Whittaker and J. A. Cotruvo (2011). "Therapeutic dose as the point of departure in assessing potential health hazards from drugs in drinking water and recycled municipal wastewater." Regulatory Toxicology and Pharmacology 60(1): 1-19.	Title
54	Buttermann, A. E. and M. Maze (1996). "Alpha-2 adrenergic agonists in anesthesiology." Seminars in Anesthesia, Perioperative Medicine and Pain 15(1): 27-40.	Title
55	Caglar Andac, S. (2017). "Determination of drugs by online column-switching liquid chromatography." Journal of chromatographic science 54(9): 1641-1647.	Full text
56	Cai, H., S. Ibayashi, H. Yao, H. Sugimori, S. Sadoshima and M. Fujishima (1995). "The α1-adrenoceptor antagonist, doxazosin, modulates the lower limit of autoregulation of cerebral blood flow during hemorrhagic hypotension in anesthetized hypertensive rats." European Journal of Pharmacology 286(3): 249-253.	Title
57	Calò, L., B. Giacon, P. A. Davis, E. Pagnin, A. Piccin, P. Riegler, W. Huber, A. Antonello and A. Semplicini (2002). "Oxidative stress and TGFbeta in kidney-transplanted patients with cyclosporin-induced hypertension. Effect of carvedilol and nifedipine." Clinical nephrology 58(2): 103-110.	Title
58	Calvo, C., B. Gil-Extremera, P. Gomez-Fernández, X. Masramon, C. Pueyo and B. Armada (2005). "Doxazosin GITS versus standard doxazosin in mild to moderate hypertension." International journal of cardiology 101(1): 97-104.	Abstract
59	Cambio, A. J. and C. P. Evans (2007). "Outcomes and quality of life issues in the pharmacological management of benign prostatic hyperplasia (BPH)." Therapeutics and clinical risk management 3(1): 181-196.	Title
60	Campo, C., J. Segura, C. Roldán, J. M. Alcázar, J. L. Rodicio and L. M. Ruilope (2003). "Doxazosin GITS versus hydrochlorothiazide as add-on therapy in patients with uncontrolled hypertension." Blood pressure. Supplement 2: 16-21.	Title
61	Caprino, L. (2000). "[Drugs for the treatment of benign prostatic hypertrophy]." Minerva Urol Nefrol 52(2): 87-92.	Title
62	Carson, C. C. and M. P. Patel (1999). "The Epidemiology, Anatomy, Physiology, and Treatment of Erectile Dysfunction in Chronic Renal Failure Patients." Advances in Renal Replacement Therapy 6(4): 296-309.	Title
63	Cases, A. (2000). "Doxazosin in a gastrointestinal therapeutic system formulation." Drugs of today (Barcelona, Spain : 1998) 36(10): 679-688.	NO Access

Sr no.	Title	Reason
64	Castelli, R., M. Tognolini, F. Vacondio, M. Incerti, D. Pala, D. Callegari, S. Bertoni, C. Giorgio, I. Hassan-Mohamed, I. Zanotti, A. Bugatti, M. Rusnati, C. Festuccia, S. Rivara, E. Barocelli, M. Mor and A. Lodola (2015). "Δ5-Cholenoyl-amino acids as selective and orally available antagonists of the Eph–ephrin system." European Journal of Medicinal Chemistry 103: 312-324.	Title
65	Cha, K.-H., T.-H. Tran, M.-S. Kim, J.-S. Kim, H. J. Park, J. Park, W. Cho and S.-J. Hwang (2010). "pH-independent sustained release matrix tablet containing doxazosin mesylate: Effect of citric acid." Archives of pharmacal research 33: 2003-2009.	Abstract
66	Chad, T., M. Ulla, V. Garnelo Rey and C. Gómez (2020). "High-Dose Insulin for Toxin Induced Cardiogenic Shock: Experience at a New High and Overview of the Evidence." The Journal of Emergency Medicine 58(2): 317-323.	Title
67	Chapman, N., C. L. Chang, B. r. Dahlöf, P. S. Sever, H. Wedel and N. R. Poulter (2008). "Effect of doxazosin gastrointestinal therapeutic system as third-line antihypertensive therapy on blood pressure and lipids in the Anglo-Scandinavian Cardiac Outcomes Trial." Circulation 118(1): 42-48.	Title
68	Chapman, N., C.-Y. Chen, T. Fujita, F. R. Hobbs, S.-J. Kim, J. A. Staessen, S. Tanomsup, J.-G. Wang and B. Williams (2010). "Time to re-appraise the role of alpha-1 adrenoceptor antagonists in the management of hypertension?" Journal of hypertension 28(9): 1796-1803.	Title
69	Chapple, C. R. (2005). "A comparison of varying α-blockers and other pharmacotherapy options for lower urinary tract symptoms." Reviews in urology 7(Suppl 4): S22.	Abstract
70	Cheung, L., D. M. T. Yu, Z. Neiron, T. W. Failes, G. M. Arndt and J. I. Fletcher (2015). "Identification of new MRP4 inhibitors from a library of FDA approved drugs using a high-throughput bioluminescence screen." Biochemical Pharmacology 93(3): 380-388.	Title
71	Chiou, C., A. H. Groll and T. J. Walsh (2000). Antifungal drugs. Side Effects of Drugs Annual. J. K. Aronson, Elsevier. 23: 289-303.	Title
72	Chrisp, P. and K. L. Goa (1990). "Dilevalol. A review of its pharmacodynamic and pharmacokinetic properties, and therapeutic potential in hypertension." Drugs 39(2): 234-263.	Review
73	Chun, I., S. Yoon, H. Gwak and Y. Kwon (2006). Pharmacokinetics of doxazosin tablet after multiple-dose administration. DRUG METABOLISM REVIEWS, TAYLOR &.	NO Access
74	Chung, M., I. Kourides, W. Canovatchel, T. Sutfin, M. Messig and R. L. Chaiken (2002). "Pharmacokinetics and pharmacodynamics of extended-release glipizide GITS compared with immediate-release glipizide in patients with type II diabetes mellitus." The Journal of Clinical Pharmacology 42(6): 651-657.	Title

Sr no.	Title	Reason
75	Chytil, L., B. Štrauch, J. Cvačka, V. Marešová, J. Widimský, R. Holaj and O. Slanař (2010). "Determination of doxazosin and verapamil in human serum by fast LC–MS/MS: Application to document non-compliance of patients." Journal of Chromatography B 878(30): 3167-3173.	NO Access
76	Conley, R., S. K. Gupta and G. Sathyan (2006). "Clinical spectrum of the osmotic-controlled release oral delivery system (OROS), an advanced oral delivery form." Current medical research and opinion 22(10): 1879-1892.	Title
77	Conway, E., J. McNeil, J. Hurley, G. Jackman, H. Krum, L. Howes and W. Louis (1993). "The effects of food on the oral bioavailability of doxazosin in hypertensive subjects." Drug Investigation 6: 90-95.	NO Access
78	Cordes, J. S., J. R. Heyen, M. L. Volberg, N. Poy, S. Kreuser, A. M. Shoieb and J. Steidl-Nichols (2016). "Validation and utility of the PhysioTel™ Digital M11 telemetry implant for cardiovascular data evaluation in cynomolgus monkeys and Beagle dogs." Journal of Pharmacological and Toxicological Methods 79: 72-79.	Title
79	Cosenzi, A., A. Sacerdote, E. Bocin, R. Molino, M. Mangiarotti and G. Bellini (1995). "Metabolic effects of atenolol and doxazosin in healthy volunteers during prolonged physical exercise." J Cardiovasc Pharmacol 25(1): 142-146.	Abstract
80	Cosenzi, A., F. L. Waltman, P. N. van Es and P. W. de Leeuw (1994). "Doxazosin versus nitrendipine: a double-blind comparative study in patients adhering to a sodium-restricted diet." Cardiovascular drugs and therapy / sponsored by the International Society of Cardiovascular Pharmacotherapy 8(3): 473-477.	Title
81	Cox, D., J. Leader, J. Milson and W. Singleton (1986). "The antihypertensive effects of doxazosin: a clinical overview." British Journal of Clinical Pharmacology 21(S1): 83S-90S.	Full text
82	Cubeddu, L., N. Fuenmayor, D. Ferry and N. Caplan (1986). CLINICAL PHARMACOKINETICS OF DOXAZOSIN IN PATIENTS WITH ESSENTIAL-HYPERTENSION. JOURNAL OF HYPERTENSION, RAPID SCIENCE PUBLISHERS 2-6 BOUNDARY ROW, LONDON, ENGLAND SE1 8NH.	NO Access
83	Cubeddu, L. X. (1988). "New alpha1-adrenergic receptor antagonists for the treatment of hypertension: Role of vascular alpha receptors in the control of peripheral resistance." American Heart Journal 116(1, Part 1): 133-162.	Title
84	Cubeddu, L. X., N. Fuenmayor, N. Caplan and D. Ferry (1987). "Clinical pharmacology of doxazosin in patients with essential hypertension." Clinical Pharmacology & Therapeutics 41(4): 439-449.	Full text
85	Cubeddu, L. X., J. L. Pool, R. Bloomfield, P. E. Klotman, B. I. Pickering, D. G. Wombolt, E. B. Nelson and A. Halperin (1988). "Effect of doxazosin monotherapy on	Title

Sr no.	Title	Reason
	blood pressure and plasma lipids in patients with essential hypertension." American journal of hypertension 1(2): 158-167.	
86	Daae, L. N., P. Kierulf, B. Brusletto, A. Westheim, I. Holme and J. O. Syvertsen (1991). "[The effect of doxazosin on blood pressure, lipids, fibrinogen and plasminogen activator inhibitor. A comparative study among smokers and non-smokers with essential hypertension]." Tidsskr Nor Laegeforen 111(9): 1102-1105.	Abstract
87	Daković-Svajcer, K. (2002). "Food and drugs." Medicinski Pregled 55(1-2): 5-12.	Title
88	Daughton, C. G. (2014). "Eco-directed sustainable prescribing: feasibility for reducing water contamination by drugs." Science of The Total Environment 493: 392-404.	Title
89	Daughton, C. G. (2014). "The Matthew Effect and widely prescribed pharmaceuticals lacking environmental monitoring: Case study of an exposure-assessment vulnerability." Science of The Total Environment 466-467: 315-325.	Title
90	de Álvaro, F. and M. A. Hernández-Presa (2006). "Effect of doxazosin gastrointestinal therapeutic system on patients with uncontrolled hypertension: the ASOCIA Study." Journal of cardiovascular pharmacology 47(2): 271-276.	Title
91	De La Garza, R., M. J. Bubar, C. L. Carbone, F. G. Moeller, T. F. Newton, N. C. Anastasio, T. A. Harper, D. L. Ware, M. A. Fuller, G. J. Holstein, J. B. Jayroe, S. I. Bandak, K. Z. Reiman, A. C. Neale, L. B. Pickford and K. A. Cunningham (2015). "Evaluation of the dopamine β-hydroxylase (DβH) inhibitor nepicastat in participants who meet criteria for cocaine use disorder." Progress in Neuro-Psychopharmacology and Biological Psychiatry 59: 40-48.	Title
92	De La Garza, R., G. P. Galloway, T. F. Newton, J. Mendelson, C. N. Haile, E. Dib, R. Y. Hawkins, C. Y. A. Chen, J. J. Mahoney, J. Mojsiak, G. Lao, A. Anderson and R. Kahn (2014). "Assessment of safety, cardiovascular and subjective effects after intravenous cocaine and lofexidine." Progress in Neuro-Psychopharmacology and Biological Psychiatry 50: 44-52.	Title
93	de Leeuw, P. W. and W. H. Birkenhäger (1988). "Alpha-adrenoceptors and the kidney." Journal of hypertension. Supplement 6(2): S21-24.	Title
94	de Medicamentos, B.-B. "Carduran XL (Bula do profissional de saúde)."	Language
95	De Mey, C. (2000). "α1-Blocker therapy for lower urinary tract symptoms suggestive of benign prostatic obstruction: what are the relevant differences in randomised controlled trials?" European urology 38(Suppl. 1): 25-39.	Title
96	de Miguel, Á. G., R. J. García, J. R. Gutiérrez, I. Á. Álvarez and A. M. García (2003). "Evaluación económica del cambio de tratamiento de doxazosina estándar a doxazosina de liberación modificada en pacientes con hipertensión leve a moderada en condiciones habituales de cuidado médico." Hipertensión y Riesgo Vascular 20(5): 200-211.	Language

Sr no.	Title	Reason
97	De Nicolò, A., V. Avataneo, F. Rabbia, G. Bonifacio, J. Cusato, C. Tomasello, E. Perlo, P. Mulatero, F. Veglio, G. Di Perri and A. D'Avolio (2016). "UHPLC–MS/MS method with protein precipitation extraction for the simultaneous quantification of ten antihypertensive drugs in human plasma from resistant hypertensive patients." Journal of Pharmaceutical and Biomedical Analysis 129: 535-541.	Title
98	Demirtunc, R., D. Duman and M. Basar (2007). "Effects of doxazosin and amlodipine on mean platelet volume and serum serotonin level in patients with metabolic syndrome: a randomised, controlled study." Clinical drug investigation 27(6): 435-441.	Title
99	Derosa, G., A. F. Cicero, A. D'Angelo, P. D. Ragonesi, L. Ciccarelli, E. Fogari, S. A. Salvadeo, I. Ferrari, A. Gravina, R. Fassi and et al. (2006). "Synergistic effect of doxazosin and acarbose in improving metabolic control in patients with impaired glucose tolerance." Clinical drug investigation 26(9): 529-539.	Title
100	Derosa, G., A. F. Cicero, A. Gaddi, A. Mugellini, L. Ciccarelli and R. Fogari (2005). "Effects of doxazosin and irbesartan on blood pressure and metabolic control in patients with type 2 diabetes and hypertension." Journal of cardiovascular pharmacology 45(6): 599-604.	Title
101	Dhaun, N., C. J. Ferro, A. P. Davenport, W. G. Haynes, J. Goddard and D. J. Webb (2007). "Haemodynamic and renal effects of endothelin receptor antagonism in patients with chronic kidney disease." Nephrol Dial Transplant 22(11): 3228-3234.	Title
102	Docherty, J. R. (1989). "The pharmacology of α1- and α2-adrenoceptors: Evidence for and against a further subdivision." Pharmacology & Therapeutics 44(2): 241-284.	Title
103	Doggrell, S. A. (2004). "After ALLHAT: doxazosin for the treatment of benign prostatic hyperplasia." Expert Opinion on Pharmacotherapy 5(9): 1957-1964.	Full text
104	Dominiak, P. (1993). "[Doxazosin. A postsynaptic alpha 1-adrenergic receptor blocker in therapy of hypertension]." Internist (Berl) 34(7): 682-687.	Title
105	Dominiak, P. (1995). "[Evaluation of the antihypertensive drugs carvedilol, doxazosin and moxonidine]." Wien Med Wochenschr 145(15-16): 373-382.	Title
106	Donnelly, R., P. A. Meredith and H. L. Elliott (1989). "Pharmacokinetic-pharmacodynamic relationships of alpha-adrenoceptor antagonists." Clin Pharmacokinet 17(4): 264-274.	Title
107	Du, H., J. Ren, S. Wang and L. He (2011). "Cell membrane chromatography competitive binding analysis for characterization of α1A adrenoreceptor binding interactions." Anal Bioanal Chem 400(10): 3625-3633.	Title
108	Eknoyan, G. (2012). Noninflammatory Vascular Disease of the Kidney. Suki and Massry's THERAPY OF RENAL DISEASES AND RELATED DISORDERS, Springer: 489-511.	Title

Sr no.	Title	Reason
109	Elama, H. S., S. M. Shalan, Y. El-Shabrawy, M. I. Eid and A. M. Zeid (2022). "Utilization of a micellar matrix for simultaneous spectrofluorimetric estimation of alfuzosin hydrochloride and vardenafil hydrochloride." Spectrochimica Acta Part A: Molecular and Biomolecular Spectroscopy 266: 120420.	Title
110	El-Chaar, G. M., M. H. Schwenk, J. Bardini, G. Caliendo, C. Frank, L. M. Profeta, K. A. Talbot and H. Cohen (1992). "NEW DRUGS ON THE HORIZON." Clinics in Podiatric Medicine and Surgery 9(2): 481-501.	Title
111	Elhilali, M. M., E. W. Ramsey, J. Barkin, R. W. Casey, R. C. Boake, G. Beland, Y. Fradet, J. Trachtenberg, W. L. Orovan, E. Schick and L. H. Klotz (1996). "A multicenter, randomized, double-blind, placebocontrolled study to evaluate the safety and efficacy of terazosin in the treatment of benign prostatic hyperplasia." Urology 47(3): 335-342.	Title
112	Elliott, H. (2002). "Selective Qy-antagonists in." Manual of Hypertension: 375.	Title
113	Elliott, H., P. Meredith, J. Vincent and J. Reid (1986). "Clinical pharmacological studies with doxazosin." British journal of clinical pharmacology 21(S1): 27S-31S.	Full text
114	Elliott, H. L., P. A. Meredith and J. L. Reid (1987). "Pharmacokinetic overview of doxazosin." The American Journal of Cardiology 59(14): G78-G81.	Full text
115	Elliott, H. L., P. A. Meredith, D. J. Sumner, K. McLean and J. L. Reid (1982). "A pharmacodynamic and pharmacokinetic assessment of a new alpha-adrenoceptor antagonist, doxazosin (UK33274) in normotensive subjects." Br J Clin Pharmacol 13(5): 699-703.	Full text
116	Erceg, M. (2011). Effects of salt forms on the oral absorption of highly permeable weak base doxazosin, University of Zagreb. Faculty of Pharmacy and Biochemistry.	Abstract
117	Erceg, M., M. Cindric, L. P. Frketic, M. Vertzoni, B. Cetina-Cižmek and C. Reppas (2010). "A LC-MS-MS method for determination of low doxazosin concentrations in plasma after oral administration to dogs." Journal of chromatographic science 48(2): 114-119.	Animal based
118	Erceg, M., M. Vertzoni, H. Cerić, M. Dumić, B. Cetina-Čižmek and C. Reppas (2012). "In vitro vs. canine data for assessing early exposure of doxazosin base and its mesylate salt." Eur J Pharm Biopharm 80(2): 402-409.	Full text
119	Erley, C. M., U. Haefele, N. Heyne, N. Braun and T. Risler (1993). "Microalbuminuria in essential hypertension. Reduction by different antihypertensive drugs." Hypertension (dallas, tex. : 1979) 21(6 Pt 1): 810-815.	Title
120	Esnault, V. L., E. A. Brown, E. Apetrei, J. Bagon, C. Calvo, R. DeChatel, H. Holdaas, S. Krcmery and Z. Kobalava (2008). "The effects of amlodipine and enalapril on renal function in adults with hypertension and nondiabetic nephropathies: a 3-year,	Title

Sr no.	Title	Reason
	randomized, multicenter, double-blind, placebo-controlled study." Clinical therapeutics 30(3): 482-498.	
121	Euctr, D. E. (2021). "Treating Nightmares in Posttraumatic Stress Disorder with the a-adrenergic Agents Clonidine and Doxazosin: a Randomized-Controlled Feasibility Study (ClonDoTrial)”." https://trialsearch.who.int/Trial2.aspx?TrialID=EUCTR2021-000319-21-DE.	Title
122	Euctr, G. B. (2005). "Blood Pressure Optimisation In Patients With Polycystic Kidney Disease And Hypertension By Rotation Through The Main Therapeutic Classes Of Antihypertensive Drugs." https://trialsearch.who.int/Trial2.aspx?TrialID=EUCTR2005-003182-16-GB.	Title
123	Everhart, E. T., P. Shwonek, P. Jacob Iii, M. C. Rowbotham and R. T. Jones (1999). "Quantitation of levorphanol in human plasma at subnanogram per milliliter levels using capillary gas chromatography with electron-capture detection." Journal of Chromatography B: Biomedical Sciences and Applications 729(1): 173-181.	Title
124	Fareed, J., P. Bacher, H. L. Messmore, J. M. Walenga, D. A. Hoppensteadt, A. Strano and R. Pifarre (1992). "Pharmacological modulation of fibrinolysis by antithrombotic and cardiovascular drugs." Progress in Cardiovascular Diseases 34(6): 379-398.	Title
125	Fareed, J., D. Hoppenstedt, R. L. Bick and P. Bacher (1992). "Drug-Induced Alterations of Hemostasis and Fibrinolysis." Hematology/Oncology Clinics of North America 6(6): 1229-1245.	Title
126	Faulx, M. D. and G. S. Francis (2008). "Adverse Drug Reactions in Patients with Cardiovascular Disease." Current Problems in Cardiology 33(12): 703-768.	Title
127	Fawzy, A., K. Braun, G. P. Lewis, M. Gaffney, K. Ice and N. D. f. t. M. S. Group (1995). "Doxazosin in the treatment of benign prostatic hyperplasia in normotensive patients: a multicenter study." The Journal of urology 154(1): 105-109.	Full text
128	Fawzy, A., V. Vashi, M. Chung, N. Dias and M. Gaffney (1999). "Clinical correlation of maximal urinary flow rate and plasma doxazosin concentrations in the treatment of benign prostatic hyperplasia." Urology 53(2): 329-335.	Full text
129	Fears, R. (1987). "10 Mode of action of lipid-lowering drugs." Baillière's Clinical Endocrinology and Metabolism 1(3): 727-754.	Title
130	Feher, M. D., A. D. Henderson, J. Wadsworth, C. Poulter, S. Gelding, W. Richmond, P. S. Sever and R. S. Elkeles (1990). "Alpha-blocker therapy; a possible advance in the treatment of diabetic hypertension--results of a cross-over study of doxazosin and atenolol monotherapy in hypertensive non-insulin dependent diabetic subjects." Journal of human hypertension 4(5): 571-577.	Title

Sr no.	Title	Reason
131	Fick, J., R. H. Lindberg, M. Tysklind and D. G. J. Larsson (2010). "Predicted critical environmental concentrations for 500 pharmaceuticals." Regulatory Toxicology and Pharmacology 58(3): 516-523.	Title
132	Fitzpatrick, J. M. and F. Desgrandchamps (2005). "The clinical efficacy and tolerability of doxazosin standard and gastrointestinal therapeutic system for benign prostatic hyperplasia." BJU Int 95(4): 575-579.	Review
133	Flack, J. M., S. V. Novikov and C. M. Ferrario (1996). "Benefits of adherence to anti-hypertensive drug therapy." European heart journal 17 Suppl A: 16-20.	Title
134	Flouvat, B., F. Fodor, A. Roux and J. R. Bianchine (1983). "Pharmacokinetics of a sustained-release trimazosin tablet formulation." American Heart Journal 106(5, Part 2): 1228-1234.	Title
135	Flynn, J. T. and S. R. Daniels (2006). "Pharmacologic treatment of hypertension in children and adolescents." The Journal of Pediatrics 149(6): 746-754.	Title
136	Foglar, R., K. Shibata, K. Horie, A. Hirasawa and G. Tsujimoto (1995). "Use of recombinant α1-adrenoceptors to characterize subtype selectivity of drugs for the treatment of prostatic hypertrophy." European Journal of Pharmacology: Molecular Pharmacology 288(2): 201-207.	Title
137	Fonseca, A. d. M., C. d. C. B. Araújo, J. H. da Silva, T. d. S. Honório, L. E. Nasciutti, L. M. Cabral, F. A. do Carmo and V. P. de Sousa (2021). "Development of transdermal based hydrogel formulations of vinorelbine with an evaluation of their in vitro profiles and activity against melanoma cells and in silico prediction of drug absorption." Journal of Drug Delivery Science and Technology 63: 102449.	Title
138	Formica, R. N., A. L. Friedman, M. I. Lorber, J. D. Smith, T. Eisen and M. J. Bia (2006). "A randomized trial comparing losartan with amlodipine as initial therapy for hypertension in the early post-transplant period." Nephrology, dialysis, transplantation 21(5): 1389-1394.	Title
139	Foster, H. E., M. Yono, D. Shin, W. Takahashi, M. Pouresmail, P. Afiatpour and J. Latifpour (2004). "Effects of chronic administration of doxazosin on α1-adrenoceptors in the rat prostate." The Journal of urology 172(6 Part 1): 2465-2470.	Animal based
140	Frick, M., P. Halttunen, P. Himanen, M. Huttunen, P. Porsti, T. Pitkajarvi, L. Poyhonen, M. Pyykonen, P. Reinikainen and P. Salmela (1986). "A long-term double-blind comparison of doxazosin and atenolol in patients with mild to moderate essential hypertension." British Journal of Clinical Pharmacology 21(S1): 55S-62S.	Full text
141	Fulton, B., A. J. Wagstaff and E. M. Sorkin (1995). "Doxazosin: an update of its clinical pharmacology and therapeutic applications in hypertension and benign prostatic hyperplasia." Drugs 49: 295-320.	Review

Sr no.	Title	Reason
142	Gaelzer, M. M. (2013). "Investigação do efeito da doxazosina sobre linhagens de glioma humano (U-138MG) e de rato (C6)."	Language
143	Galceran, F., F. A. Digirolamo, M. Rengifo, C. Reigada, M. Saye, B. J. Maciel, I. G. Estecho, A. E. Errasti, C. A. Pereira and M. R. Miranda (2023). "Identifying inhibitors of Trypanosoma cruzi nucleoside diphosphate kinase 1 as potential repurposed drugs for Chagas' disease." Biochemical Pharmacology 216: 115766.	Title
144	Gallaher, C. K. (1999). "Effects of atenolol, doxazosin, and enalapril on measures of insulin metabolic syndrome and cardiovascular function at rest and in response to stress in hypertension."	Title
145	Gallaher, C. K. (2000). "Effects of atenolol, doxazosin, and enalapril on measures of insulin metabolic syndrome and cardiovascular function at rest and in response to stress in hypertension." Dissertation abstracts international: section b: the sciences and engineering 61: 1126.	Title
146	Ganzinger, U. (1989). "[New specialty drugs registered in Austria. Supressin]." Wien Klin Wochenschr 101(20): 714-720.	Title
147	Gao, N., H. Wu, Y. Chang, X. Guo, L. Zhang, L. Du and Y. Fu (2015). "Mixed micelle cloud point-magnetic dispersive μ-solid phase extraction of doxazosin and alfuzosin." Spectrochim Acta A Mol Biomol Spectrosc 134: 10-16.	Title
148	Garber, A. J., M. J. Abrahamson, J. I. Barzilay, L. Blonde, Z. T. Bloomgarden, M. A. Bush, S. Dagogo-Jack, M. B. Davidson, D. Einhorn, W. T. Garvey, G. Grunberger, Y. Handelsman, I. B. Hirsch, P. S. Jellinger, J. B. McGill, J. I. Mechanick, P. D. Rosenblit, G. E. Umpierrez and M. H. Davidson (2013). "American Association of Clinical Endocrinologists' Comprehensive Diabetes Management Algorithm 2013 Consensus Statement." Endocrine Practice 19: 1-48.	Title
149	Genovese, I., A. Ilari, Y. G. Assaraf, F. Fazi and G. Colotti (2017). "Not only P-glycoprotein: Amplification of the ABCB1-containing chromosome region 7q21 confers multidrug resistance upon cancer cells by coordinated overexpression of an assortment of resistance-related proteins." Drug Resistance Updates 32: 23-46.	Title
150	Gerónimo-Pardo, M., A. B. Cuartero-del-Pozo, J. M. Jiménez-Vizuete, M. Cortiñas-Sáez and R. Peyró-García (2005). "Clarithromycin-nifedipine interaction as possible cause of vasodilatory shock." Ann Pharmacother 39(3): 538-542.	Title
151	Ghofrani, H. A., O. Distler, F. Gerhardt, M. Gorenflo, E. Grünig, W. E. Haefeli, M. Held, M. M. Hoeper, C. M. Kähler, H. Kaemmerer, H. Klose, V. Köllner, B. Kopp, S. Mebus, A. Meyer, O. Miera, D. Pittrow, G. Riemekasten, S. Rosenkranz, D. Schranz, R. Voswinckel and H. Olschewski (2011). "Treatment of pulmonary arterial hypertension	Title

Sr no.	Title	Reason
	(PAH): Updated Recommendations of the Cologne Consensus Conference 2011." International Journal of Cardiology 154: S20-S33.	
152	Gimeno-Gracia, M., J. Sánchez-Rubio-Ferrández, M. de las Aguas Robustillo-Cortés and R. Morillo-Verdugo (2020). "Prevalence of polypharmacy and pharmacotherapy complexity in elderly people living with HIV in Spain. POINT study." Farmacia Hospitalaria 44(4): 127-134.	Title
153	Giorda, C., M. Appendino, M. G. Mason, E. Imperiale and G. Pagano (1995). "Alpha 1-blocker doxazosin improves peripheral insulin sensitivity in diabetic hypertensive patients." Metabolism: clinical and experimental 44(5): 673-676.	Title
154	Giordano, M., P. Castellino, A. Solini, M. L. Canessa and R. A. DeFronzo (1997). "Na+/Li+ and Na+/H+ countertransport activity in hypertensive non-insulin-dependent diabetic patients: role of insulin resistance and antihypertensive treatment." Metabolism 46(11): 1316-1323.	Title
155	Giordano, M., M. Matsuda, L. Sanders, M. L. Canessa and R. A. DeFronzo (1995). "Effects of angiotensin-converting enzyme inhibitors, Ca2+ channel antagonists, and alpha-adrenergic blockers on glucose and lipid metabolism in NIDDM patients with hypertension." Diabetes 44(6): 665-671.	Title
156	Giordano, M., L. R. Sanders, P. Castellino, M. L. Canessa and R. A. DeFronzo (1996). "Effect of alpha-adrenergic blockers, ACE inhibitors, and calcium channel antagonists on renal function in hypertensive non-insulin-dependent diabetic patients." Nephron 72(3): 447-453.	Title
157	Glanz, M., A. J. Garber, G. Mancia and M. Levenstein (2001). "Meta-analysis of studies using selective α1-blockers in patients with hypertension and type 2 diabetes." International journal of clinical practice 55(10): 694-701.	Title
158	Goldsmith, D. R. and G. L. Plosker (2005). "Doxazosin gastrointestinal therapeutic system: a review of its use in benign prostatic hyperplasia." Drugs 65: 2037-2047.	Review
159	Gonzaga, E. V., S. S. Vieira, J. s. L. Vilaca, M. Guerra, M. G. Evangelista, I. M. Rosa, M. M. Figueiredo, O. M. Viana and A. C. Doriguetto (2019). "Doxazosin Free-Base Structure Determination and Its Equilibrium Solubility Compared to Polymorphic Doxazosin Mesylate Forms A and H." Crystal Growth & Design 19(2): 737-746.	Full text
160	González Maqueda, I. (1994). "Adrenoreceptors, endothelial function, and lipid profile: effects of atenolol, doxazosin, and carvedilol." Coronary artery disease 5(11): 909-918.	Title
161	Gorzoni, M. L., R. M. Alves Fabbri and S. Luciano Pires (2012). "Potentially inappropriate medications in elderly." Revista da Associação Médica Brasileira (English Edition) 58(4): 442-446.	Title

Sr no.	Title	Reason
162	Graham, R. M. (1984). "Selective alpha1-adrenergic antagonists: Therapeutically relevant antihypertensive agents." The American Journal of Cardiology 53(3): A16-A20.	Title
163	Grossman, E. and F. H. Messerli (2004). "Calcium antagonists." Progress in Cardiovascular Diseases 47(1): 34-57.	Title
164	Grzeszczak, W. (2000). "Cardura XL--a unique drug formulation--doxazosine administered in a slow-release form (doxazosine GITS)." Przeglad Lekarski 57(11): 643-654.	No Access
165	Grzeszczak, W. (2002). "Praktyczne podejście do leczenia nadciśnienia tętniczego u chorych z zaburzeniami metabolicznymi oraz z łagodnym przerostem stercza." Diabetologia Kliniczna 3(1): 1-8.	Language
166	Guay, D. R. (2004). "Extended-release alfuzosin hydrochloride: a new alpha-adrenergic receptor antagonist for symptomatic benign prostatic hyperplasia." Am J Geriatr Pharmacother 2(1): 14-23.	Title
167	Gul, A., S. Coban, A. R. Turkoglu, M. Guzelsoy, M. Ozturk and N. A. Kankilic (2020). "Comparative efficacy and safety profile of 4 vs 8 mg of silodosin once daily usage in patients with benign prostatic hyperplasia–related lower urinary tract symptoms divided into subgroups according to International Prostate Symptom Score severity." Prostate International 8(4): 152-157.	Title
168	Gundersen, P. O. M., A. Helland, O. Spigset and S. Hegstad (2018). "Quantification of 21 antihypertensive drugs in serum using UHPLC-MS/MS." Journal of Chromatography B 1089: 84-93.	Title
169	Guo, J. and R. Tang (2021). "Efficacy and tolerability of doxazosin gastro-intestinal therapeutic system versus tamsulosin in patients with lower urinary tract symptoms associated with benign prostatic hyperplasia: A systematic review and meta-analysis." Medicine 100(33).	Abstract
170	Guo, X., H. Wu, S. Guo, Y. Shi, J. Du, P. Zhu and L. Du (2016). "Highly Sensitive Fluorescence Methods for the Determination of Alfuzosin, Doxazosin, Terazosin and Prazosin in Pharmaceutical Formulations, Plasma and Urine." Anal Sci 32(7): 763-768.	Abstract
171	Gur, S., P. J. Kadowitz and W. J. Hellstrom (2008). "Guide to drug therapy for lower urinary tract symptoms in patients with benign prostatic obstruction: implications for sexual dysfunction." Drugs 68: 209-229.	Title
172	Gurny, R. and D. N. Loggia "Strategies to design push-pull osmotic systems for delivering low and pH-dependent soluble drugs."	Title
173	Gzyl-Malcher, B., J. Handzlik and E. Klekowska (2012). "Interaction of prazosin with model membranes — A Langmuir monolayer study." Bioelectrochemistry 87: 96-103.	Title

Sr no.	Title	Reason
174	Haass-Koffler, C. L., K. Goodyear, W. H. Zywiak, M. Magill, S. E. Eltinge, P. M. Wallace, V. M. Long, N. Jayaram-Lindström, R. M. Swift, G. A. Kenna and et al. (2017). "Higher pretreatment blood pressure is associated with greater alcohol drinking reduction in alcohol-dependent individuals treated with doxazosin." Drug and alcohol dependence 177: 23-28.	Title
175	Haass-Koffler, C. L., L. Leggio and R. M. Swift (2017). "The alpha1 blockade by doxazosin in regulation of alcohol drinking in alcohol dependent patients." Alcoholism: clinical and experimental research. Conference: 40th annual scientific meeting of the research society on alcoholism. United states 41: 324A.	Title
176	Haenni, A. and H. Lithell (1994). "Treatment with a β-blocker with β2-agonism improves glucose and lipid metabolism in essential hypertension." Metabolism 43(4): 455-461.	Title
177	Hajjar, I. (2005). "Postural blood pressure changes and orthostatic hypotension in the elderly patient: impact of antihypertensive medications." Drugs & aging 22: 55-68.	Title
178	Hall, G. C. and A. D. McMahon (2007). "Comparative study of modified release alpha-blocker exposure in elderly patients with fractures." Pharmacoepidemiology and Drug Safety 16(8): 901-907.	Title
179	Hamilton, C. A., J. L. Reid and J. Vincent (1985). "Pharmacokinetic and pharmacodynamic studies with two alpha-adrenoceptor antagonists, doxazosin and prazosin in the rabbit." British journal of pharmacology 86(1): 79.	Animal based
180	Hanada, K., K. Asari, M. Saito, J.-i. Kawana, M. Mita and H. Ogata (2008). "Comparison of pharmacodynamics between carvedilol and metoprolol in rats with isoproterenol-induced cardiac hypertrophy: Effects of carvedilol enantiomers." European Journal of Pharmacology 589(1): 194-200.	Title
181	Hanes, D. S., M. R. Weir and J. R. Sowers (1996). "Gender considerations in hypertension pathophysiology and treatment." The American Journal of Medicine 101(3, Supplement 1): 10S-21S.	Title
182	Hariyadi, D. M. and N. Rosita (2020). "Characterization and antibacterial activity of cocos Nucifera L. Meat extract and powder as a drug and cosmetic agent." International Journal of Research in Pharmaceutical Sciences 11(1): 611-616.	Title
183	Hartung, R., H. Matzkin, A. Alcaraz, M. Emberton, N. Harving, J. van Moorselaar, M. Elhilali and G. Vallancien (2006). "Age, Comorbidity and Hypertensive Co-Medication do Not Affect Cardiovascular Tolerability of 10 Mg Alfuzosin Once Daily." The Journal of Urology 175(2): 624-628.	Title
184	Hayduk, K. and H. T. Schneider (1987). "Antihypertensive effects of doxazosin in systemic hypertension and comparison with terazosin." Am J Cardiol 59(14): 95g-98g.	Abstract

Sr no.	Title	Reason
185	Hermida, R. C., D. E. Ayala and C. Calvo (2005). "Administration-time-dependent effects of antihypertensive treatment on the circadian pattern of blood pressure." Curr Opin Nephrol Hypertens 14(5): 453-459.	Title
186	Hermida, R. C., C. Calvo, D. E. Ayala, M. J. Domínguez, M. Covelo, J. R. Fernández, M. J. Fontao and J. E. López (2004). "Administration-time-dependent effects of doxazosin GITS on ambulatory blood pressure of hypertensive subjects." Chronobiology international 21(2): 277-296.	Abstract
187	Hermida, R. C. and M. H. Smolensky (2004). "Chronotherapy of hypertension." Curr Opin Nephrol Hypertens 13(5): 501-505.	Title
188	Hernandez, C., R. Duran, J. Jara, I. Castano and M. Moralejo (2005). "Controlled-release doxazosin in the treatment of benign prostatic hyperplasia." Prostate Cancer and Prostatic Diseases 8(4): 375-380.	Abstract
189	Hernández Fernández, C., I. Moncada Iribarren, J. Jara Rascón, I. Castaño González and M. Moralejo Gárate (2004). "Tratamiento con doxazosina en 3.347 pacientes con síntomas del tracto urinario inferior: Efecto sobre su función sexual. Estudio impros." Actas Urológicas Españolas 28(4): 290-297.	Language
190	Hernández Hernández, R., M. Angeli-Greaves, A. R. Carvajal, J. Guerrero Pajuelo, M. C. Armas Padilla and M. J. Armas-Hernández (1996). "Terazosin: ex vivo and in vitro platelet aggregation effects in patients with arterial hypertension." Am J Hypertens 9(5): 437-444.	Title
191	Hernandez Hernandez, R., A. R. Carvajal, J. Guerrero Pajuelo, M. J. Armas de Hernandez, M. C. Armas Padilla, O. Barragan, J. J. Boada Boada and E. Roa (1991). "The effect of doxazosin on platelet aggregation in normotensive subjects and patients with hypertension: An in vitro study." American Heart Journal 121(1, Part 2): 389-394.	Title
192	Heydorn, W. E. (1997). "The 98th Annual Meeting of the American Society for Clinical Pharmacology and Therapeutics." Expert Opinion on Investigational Drugs 6(4): 453-457.	Title
193	Hieble, J. P., D. C. Kolpak, G. P. McCafferty, R. R. Ruffolo, R. Testa and A. Leonardi (1999). "Effects of α1-adrenoceptor antagonists on agonist and tilt-induced changes in blood pressure: relationships to uroselectivity." European Journal of Pharmacology 373(1): 51-62.	Title
194	Hobbs, F. R., T. Khan and B. Collins (2005). "Doxazosin versus bendrofluazide: a comparison of the metabolic effects in British South Asians with hypertension." British journal of general practice 55(515): 437-443.	Title

Sr no.	Title	Reason
195	Hobbs, R. (2004). "Greater improvement in glycemic control and lipid profile in British Asians having high blood pressure with doxazosin versus bendrofluazide therapy." Diabetes 53: A128-129.	Title
196	Hodin, R., C. Lubitz, R. Phitayakorn and A. Stephen (2014). "Diagnosis and management of pheochromocytoma." Current Problems in Surgery 51(4): 151-187.	Title
197	Holanda, V. A. D., M. C. Oliveira, E. D. da Silva Junior and E. C. Gavioli (2022). "Tamsulosin facilitates depressive-like behaviors in mice: Involvement of endogenous glucocorticoids." Brain Research Bulletin 178: 29-36.	Title
198	Holme, I., P. Fauchald, H. E. Rugstad and H. P. Stokke (1991). "Preliminary results of the Norwegian doxazosin postmarketing surveillance study: A twelveweek experience." American Heart Journal 121(1, Part 2): 260-267.	Title
199	Hoogerbrugge, N., E. de Groot, L. H. de Heide, M. A. de Ridder, J. C. Birkenhägeri, T. Stijnen and H. Jansen (2002). "Doxazosin and hydrochlorothiazide equally affect arterial wall thickness in hypertensive males with hypercholesterolaemia (the DAPHNE study). Doxazosin Atherosclerosis Progression Study in Hypertensives in the Netherlands." Neth J Med 60(9): 354-361.	Title
200	Hoot, N. R., J. G. Benitez and K. H. Palm (2013). "Hemodynamically Unstable: Accidental Atenolol Toxicity?" The Journal of Emergency Medicine 45(3): 355-357.	Title
201	Howes, L. G., D. Lykos and G. C. Rennie (1996). "Effects of antihypertensive drugs on coronary artery disease risk: a meta-analysis." Clinical and experimental pharmacology & physiology 23(6-7): 555-558.	Title
202	Humphreys, J. and M. Waite (1989). "ALPHA-1 BLOCKERS. A NEW GENERATION OF ANTIHYPERTENSIVE AGENTS." Journal of clinical pharmacy and therapeutics 14(4): 263-283.	Title
203	Hysek, C. M. and M. E. Liechti "Effects of MDMA on the pupillary light reflex on its own and after pretreatment with reboxetine, duloxetine, clonidine, carvedilol, and doxazosin."	Title
204	Hysek, C. M. and M. E. Liechti (2012). "Effects of MDMA alone and after pretreatment with reboxetine, duloxetine, clonidine, carvedilol, and doxazosin on pupillary light reflex." Psychopharmacology 224: 363-376.	Title
205	Ingrid, O. and H. P. STOKKE (1999). "Doxazosin GITS compared with doxazosin standard and placebo in patients with mild hypertension." Blood pressure 8(3): 184-191.	Abstract
206	Ishikawa, J., S. Hoshide, S. Shibasaki, Y. Matsui, T. Kabutoya, K. Eguchi, S. Ishikawa, T. G. Pickering, K. Shimada and K. Kario (2006). "The Japan Morning Surge-1 (JMS-1) study: protocol description." Hypertension research 29(3): 153-159.	Title

Sr no.	Title	Reason
207	Ismail, M. and H. Hashim (2012). "Dutasteride/tamsulosin fixed-dose combination for the treatment of benign prostatic enlargement." Drugs of today (Barcelona, Spain : 1998) 48(1): 17-24.	Title
208	Isrctn (2007). "Candesartan in renal artery stenosis (CARLAS)." https://trialsearch.who.int/Trial2.aspx?TrialID=ISRCTN35143689.	Title
209	Isrctn (2013). "A study to test whether spironolactone and dietary nitrate (as beetroot juice) alter blood vessel stiffness in Type 2 diabetes." https://trialsearch.who.int/Trial2.aspx?TrialID=ISRCTN25003627.	Title
210	Ito, K., H. Ohtani and Y. Sawada (2007). "Assessment of alpha1-adrenoceptor antagonists in benign prostatic hyperplasia based on the receptor occupancy theory." Br J Clin Pharmacol 63(4): 394-403.	Full text
211	JA, L. G. (2004). "Doxazosin in the gastrointestinal therapeutic system (GITS) formulation and trial without catheter after acute urinary retention due to BPH. Dose increase action on recovery effect." Actas Urologicas Espanolas 28(1): 32-37.	Title
212	Jackman, G. P., F. Colagrande and W. J. Louis (1991). "Validation of a solid-phase extraction high-performance liquid chromatographic assay for doxazosin." J Chromatogr 566(1): 234-238.	No Access
213	Jager, L. P., G. J. De Graaf and H. C. A. Widjaja-Greefkes (1996). "Screening for drug-induced alterations in the production and release of steroid hormones by porcine adrenocortical cells in vitro." Toxicology in Vitro 10(5): 595-608.	Title
214	Jagroop, I. A. and D. P. Mikhailidis (2001). "Doxazosin, an alpha1-adrenoceptor antagonist, inhibits serotonin-induced shape change in human platelets." J Hum Hypertens 15(3): 203-207.	Title
215	Jamal, A., L. Shahzadi, S. Ahtzaz, S. Zahid, A. A. Chaudhry, I. U. Rehman and M. Yar (2018). "Identification of anti-cancer potential of doxazocin: Loading into chitosan based biodegradable hydrogels for on-site delivery to treat cervical cancer." Mater Sci Eng C Mater Biol Appl 82: 102-109.	Title
216	Janknegt, R. A. (1995). "Profile of doxazosin in patients with benign prostatic hyperplasia." Scandinavian journal of urology and nephrology. Supplementum 168: 21-27.	Abstract
217	Jáuregui-Garrido, B. and I. Jáuregui-Lobera (2012). "Interacciones entre fármacos antihipertensivos y alimentos." Nutrición Hospitalaria 27(6): 1866-1875.	Language
218	Jazbar, J., I. Locatelli, N. Horvat and M. Kos (2018). "Clinically relevant potential drug–drug interactions among outpatients: A nationwide database study." Research in Social and Administrative Pharmacy 14(6): 572-580.	Title

Sr no.	Title	Reason
219	Jelski, J. and M. Speakman (2012). "Alpha adrenoreceptor antagonists–have we reached the optimum balance of safety/efficacy?" Drug Discovery Today: Therapeutic Strategies 9(1): e27-e33.	Title
220	Jeng, J. R., W. H. Sheu, C. Y. Jeng, S. H. Huang and S. M. Shieh (1996). "Effect of doxazosin on fibrinolysis in hypertensive patients with and without insulin resistance." Am Heart J 132(4): 783-789.	Title
221	Jensen, B. P., J. M. Dalrymple and E. J. Begg (2013). "Transfer of doxazosin into breast milk." J Hum Lact 29(2): 150-153.	Full text
222	Jhang, J. F., Y. H. Jiang and H. C. Kuo (2013). "Adding Cyclooxygenase-2 inhibitor to alpha blocker for patients with benign prostate hyperplasia and elevated serum prostate specific antigen could not improve prostate biopsy detection rate but improve lower urinary tract symptoms." International journal of clinical practice 67(12): 1327-1333.	Title
223	Jie, K., P. Van Brummelen and P. Vermey (1984). "Identification of vascular postsynaptic alpha1- and alpha2-adrenoceptors in man." Circulation research 54(4): 447-452.	Title
224	Jie, K., P. Van Brummelen, P. Vermey, P. B. Timmermans and P. A. Van Zwieten (1987). "Postsynaptic alpha 1- and alpha 2-adrenoceptors in human blood vessels: interactions with exogenous and endogenous catecholamines." European journal of clinical investigation 17(2): 174-181.	Title
225	Johnson, T. N. and A. H. Thomson (2008). "Pharmacokinetics of drugs in liver." Drugs and the Liver: A Guide to Drug Handling in Liver Dysfunction: 103.	Title
226	Jones, D. W. and C. D. Sands (1993). "Effects of doxazosin and hydrochlorothiazide on lipid levels in Korean patients with essential hypertension." Journal of cardiovascular pharmacology 22(3): 431-437.	Title
227	Joubert, P. (2004). Antihypertensive drugs. Side Effects of Drugs Annual. J. K. Aronson, Elsevier. 27: 213-218.	Title
228	Julius, S. (1991). "Clinical implications of pathophysiologic changes in the midlife hypertensive patient." American Heart Journal 122(3, Part 2): 886-891.	Title
229	Kao, D. P., B. D. Lowes, E. M. Gilbert, W. Minobe, L. E. Epperson, L. K. Meyer, D. A. Ferguson, A. K. Volkman, R. Zolty, C. D. Borg and et al. (2015). "Therapeutic Molecular Phenotype of β-Blocker-Associated Reverse-Remodeling in Nonischemic Dilated Cardiomyopathy." Circulation. Cardiovascular genetics 8(2): 270-283.	Title
230	Kao, D. P., W. Minobe, L. E. Epperson, L. Meyer, D. Ferguson, R. Zolty, E. M. Gilbert, B. D. Lowes and M. R. Bristow (2013). "Serial gene expression changes associated with reverse remodeling in dilated cardiomyopathies: results of the effects of beta-blockers on	Title

Sr no.	Title	Reason
	remodeling and gene expression in the failing human heart (BORG) trial (NCT01798992)." Circulation 128(22).	
231	Kaplan, N. M. (1993). "Southwestern Internal Medicine Conference: The Promises and Perils of Treating the Elderly Hypertensive." The American Journal of the Medical Sciences 305(3): 183-197.	Title
232	Kaplan, S. A. and P. M. D'Alisera (1998). "Tolerability of α-blockade with doxazosin as a therapeutic option for symptomatic benign prostatic hyperplasia in the elderly patient: a pooled analysis of seven double-blind, placebo-controlled studies." The Journals of Gerontology Series A: Biological Sciences and Medical Sciences 53(3): M201-M206.	Title
233	Kapri, A., N. Gupta and G. Raj (2019). "A rapid and sensitive reversed phase liquid chromatography-tandem mass spectrometry method for quantification of doxazosin mesylate in human plasma using doxazosin mesylate D8 as internal standard." Analytical Chemistry Letters 9(3): 403-417.	Full text
234	Karaźniewicz-Łada, M., D. Danielak, A. Teżyk, C. Żaba, G. Tuffal and F. Główka (2012). "HPLC–MS/MS method for the simultaneous determination of clopidogrel, its carboxylic acid metabolite and derivatized isomers of thiol metabolite in clinical samples." Journal of Chromatography B 911: 105-112.	Title
235	Kaye, B., N. J. Cussans and J. K. Faulkner (1986). "The metabolism and kinetics of doxazosin in man, mouse, rat and dog." British journal of clinical pharmacology 21(SUPPL. 1): 19S-25S.	Full text
236	Kenna, G. A., C. L. Haass-Koffler, W. H. Zywiak, S. M. Edwards, M. B. Brickley, R. M. Swift and L. Leggio (2016). "Role of the alpha1 blocker doxazosin in alcoholism: a proof-of-concept randomized controlled trial." Addiction biology 21(4): 904-914.	Title
237	Kenny, B. A., A. M. Naylor, A. J. Carter, A. M. Read, P. M. Greengrass and M. G. Wyllie (1994). "Effect of alpha, adrenoceptorantagonists on prostatic pressure and blood pressure in the anesthetized dog." Urology 44(1): 52-57.	Title
238	Kim, J. and H. Nguyen (2020). "Doxazosin."	Title
239	Kim, S., J. M. Park, S. Park, E. Jung, D. Ko, M. Park, J. Seo, K. D. Nam, Y. K. Kang and K. Lee (2023). "Suppression of TNBC metastasis by doxazosin, a novel dual inhibitor of c-MET/EGFR." Journal of Experimental & Clinical Cancer Research 42(1): 292.	Title
240	Kinoshita, M., N. Shimazu, M. Fujita, Y. Fujimaki, K. Kojima, Y. Mikuni, E. Horie and T. Teramoto (2001). "Doxazosin, an alpha1-adrenergic antihypertensive agent, decreases serum oxidized LDL." Am J Hypertens 14(3): 267-270.	Title

Sr no.	Title	Reason
241	Kirby, R. (1998). "Morning versus evening dosing with doxazosin in benign prostatic hyperplasia: Pharmacokinetics, efficacy and safety." International journal of clinical practice 52(2): 75-77.	No Access
242	Kirby, R. and A. Jardin (1997). "Doxazosin in the treatment of benign prostatic hyperplasia. A review of the safety profile in older patients." Prostate Cancer and Prostatic Diseases 1(2): 84-89.	Review
243	Kjeldsen, S. E., R. K. Gupta, L. Krause, A. B. Weder and S. Julius (1992). "Does blood pressure reduction necessarily compromise cardiac function or renal hemodynamics? Effects of the angiotensin-converting enzyme inhibitor quinapril." American Heart Journal 123(5): 1433-1438.	Title
244	Kleiman, N. S. and R. M. Califf (2000). "Results from late-breaking clinical trials sessions at ACCIS 2000 and ACC 2000." Journal of the American College of Cardiology 36(1): 310-325.	Title
245	Kong, D., Q. Li, P. Zhang, W. Zhang, Y. Zhen and L. Ren (2015). "The truth about the lower plasma concentration of the (-)-isomer after racemic doxazosin administration in rats: Stereoselective inhibition of the (-)-isomer by the (+)-isomer at CYP3A." Eur J Pharm Sci 77: 238-245.	Animal based
246	Kong, D., Y. Tian, K. Duan, W. Guo, Q. Zhang, P. Zhang, Z. Yang, X. Qin, L. Ren and W. Zhang (2022). "Elucidating a Complicated Enantioselective Metabolic Profile: A Study From Rats to Humans Using Optically Pure Doxazosin." Frontiers in Pharmacology 13: 834897.	Full text
247	Korstanje, C. and W. Krauwinkel (2011). "Specific pharmacokinetic aspects of the urinary tract." Urinary Tract: 267-282.	Title
248	Kowala, M. C., R. I. Grove and G. Aberg (1994). "Inhibitors of angiotensin converting enzyme decrease early atherosclerosis in hyperlipidemic hamsters. Fosinopril reduces plasma cholesterol and captopril inhibits macrophage—foam cell accumulation independently of blood pressure and plasma lipids." Atherosclerosis 108(1): 61-72.	Title
249	Kowala, M. C., J. J. Nunnari, S. K. Durham and R. J. Nicolosi (1991). "Doxazosin and cholestyramine similarly decrease fatty streak formation in the aortic arch of hyperlipidemic hamsters." Atherosclerosis 91(1): 35-49.	Title
250	Krai, J., A. Beckenkamp, M. Gaelzer, A. Pohlmann, S. Guterres, E. Filippi-Chiela, C. Salbego, A. Buffon and R. Beck (2017). "Doxazosin nanoencapsulation improves its in vitro antiproliferative and anticlonogenic effects on breast cancer cells." Biomedicine & Pharmacotherapy 94: 10-20.	Abstract

Sr no.	Title	Reason
251	Krusell, L. R., C. K. Christensen and O. L. Pedersen (1992). "Alpha-adrenoceptor blockade in patients with mild to moderate hypertension: long-term renal effects of doxazosin." Journal of cardiovascular pharmacology 20(3): 440-444.	Abstract
252	Kubacka, M., M. Kotańska, M. Szafarz, K. Pociecha, A. M. Waszkielewicz, H. Marona, B. Filipek and S. Mogilski (2019). "Beneficial effects of non-quinazoline α1-adrenolytics on hypertension and altered metabolism in fructose-fed rats. A comparison with prazosin." Nutrition, Metabolism and Cardiovascular Diseases 29(7): 751-760.	Title
253	Kubacka, M., S. Mogilski, B. Filipek and H. Marona (2013). "The hypotensive activity and alpha1-adrenoceptor antagonistic properties of some aroxyalkyl derivatives of 2-methoxyphenylpiperazine." European Journal of Pharmacology 698(1): 335-344.	Title
254	Kumar, A., A. Siwach and P. Verma (2022). "An overview of the synthetic route to the marketed formulations of pyrimidine: a review." Mini Reviews in Medicinal Chemistry 22(6): 884-903.	Title
255	Kuo, G.-H., C. Prouty, W. V. Murray, V. Pulito, L. Jolliffe, P. Cheung, S. Varga, M. Evangelisto and C. Shaw (2000). "Design, synthesis and biological evaluation of pyridine-phenylpiperazines: A novel series of potent and selective α1a-adrenergic receptor antagonist." Bioorganic & Medicinal Chemistry 8(9): 2263-2275.	Title
256	Labiós, M., M. Martínez, F. Gabriel, V. Guiral, S. Ruiz-Aja and J. Aznar (2006). "Cytoplasmic free calcium mobilization in platelets, expression of P-selectin, phosphatidylserine, and microparticle formation, measured by whole blood flow cytometry, in hypertensive patients. Effect of doxazosin GITS." Thrombosis research 117(4): 403-409.	Title
257	Lämmerhofer, M. and W. Lindner (2000). Chapter 9 Recent developments in liquid chromatographic enantioseparation. Handbook of Analytical Separations. K. Valkó, Elsevier Science B.V. 1: 337-437.	Title
258	Lara-Ramirez, E. E., J. C. López-Cedillo, B. Nogueda-Torres, M. Kashif, C. Garcia-Perez, V. Bocanegra-Garcia, R. Agusti, M. L. Uhrig and G. Rivera (2017). "An in vitro and in vivo evaluation of new potential trans-sialidase inhibitors of Trypanosoma cruzi predicted by a computational drug repositioning method." European Journal of Medicinal Chemistry 132: 249-261.	Title
259	Lee, S. H., B. H. Chung, S. J. Kim, J. H. Kim, J. C. Kim and J. Y. Lee (2011). "Initial combined treatment with anticholinergics and alpha-blockers for men with lower urinary tract symptoms related to BPH and overactive bladder: a prospective, randomized, multi-center, double-blind, placebo-controlled study." Prostate cancer and prostatic diseases 14(4): 320-325.	Title

Sr no.	Title	Reason
260	Lee, S. H., C. Y. Oh, K. K. Park, M. S. Chung, S. J. Yoo and B. H. Chung (2011). "Comparison of the clinical efficacy of medical treatment of symptomatic benign prostatic hyperplasia between normal and obese patients." Asian Journal of Andrology 13(5): 728.	Title
261	Lee, S. Y. and M.-G. Kim (2016). "Effect of Modulated Electrohyperthermia on the Pharmacokinetics of Oral Transmucosal Fentanyl Citrate in Healthy Volunteers." Clinical Therapeutics 38(12): 2548-2554.	Title
262	Lehtonen, A., P. Himanen, M. Saraste, K. Niittymäki and J. Marniemi (1986). "Double-blind comparison of the effects of long-term treatment with doxazosin or atenolol on serum lipoproteins." Br J Clin Pharmacol 21 Suppl 1(Suppl 1): 77s-81s.	Full text
263	Lemmer, B. and G. Nold (2003). "Effect of doxazosin GITS on 24-hour blood pressure profile in patients with stage 1 to stage 2 primary hypertension." Blood Pressure Monitoring 8(3): 119-125.	Title
264	Leonova, M. V. e. (2009). "Klinicheskoe znachenie peroral'nykh lekarstvennykh form al'fag adrenoblokatorov modifitsirovannogo vysvobozhdeniya v lechenii patsientov s dobrokachestvennoy gipertrofiey predstatel'noy zhelezy." Pharmateca(20): 36-43.	Language
265	Li, Q., D. Kong, Q. Du, J. Zhao, Y. Zhen, T. Li and L. Ren (2015). "Enantioselective pharmacokinetics of doxazosin and pharmacokinetic interaction between the isomers in rats." Chirality 27(10): 738-744.	Animal based
266	Li, X. H., H. Sun, L. X. Zhou, J. J. Yang, H. T. Xie and Y. N. Zhao (2015). "Pharmacokinetics and bioequivalence of doxazosin mesylate extended-release tablets in healthy Chinese volunteers." Chinese journal of new drugs 24(2): 177-181.	No Access
267	Lin, H. L., S. Y. Lin, Y. K. Lin, H. O. Ho, Y. W. Lo and M. T. Sheu (2008). "Release characteristics and in vitro-in vivo correlation of pulsatile pattern for a pulsatile drug delivery system activated by membrane rupture via osmotic pressure and swelling." Eur J Pharm Biopharm 70(1): 289-301.	Title
268	Lin, S.-C., S.-C. Chueht, C.-J. Hsiao, T.-K. Li, T.-H. Chen, C.-H. Liao, P.-C. Lyu and J.-H. Guh (2007). "Prazosin Displays Anticancer Activity against Human Prostate Cancers: Targeting DNA, Cell Cycle." Neoplasia 9(10): 830-839.	Title
269	Lindblad, C. I., J. T. Hanlon, C. R. Gross, R. J. Sloane, C. F. Pieper, E. R. Hajjar, C. M. Ruby, K. E. Schmader and P. Multidisciplinary Consensus (2006). "Clinically important drug-disease interactions and their prevalence in older adults." Clinical Therapeutics 28(8): 1133-1143.	Title
270	Llorente, C., M. Ruiz, J. Rejas Gutiérrez, M. Esteban, F. Villasante and A. Hareendran (2006). "Linguistic Adaptation and Validation of the Spanish Version of the Benign	Title

Sr no.	Title	Reason
	Prostatic Hyperplasia-Patient Impact Measure (BPH-PIM®)." Clinical drug investigation 26: 103-112.	
271	Locatelli, I., V. Kmetec, A. Mrhar and I. Grabnar (2005). "Determination of warfarin enantiomers and hydroxylated metabolites in human blood plasma by liquid chromatography with achiral and chiral separation." Journal of Chromatography B 818(2): 191-198.	Title
272	Loh, L.-M. (1986). Single Dose and Chronic Dose Pharmacokinetics of Doxazosin in Mild to Moderate Hypertensive Patients, Victorian College of Pharmacy.	No Access
273	Longhurst, H. J., M. Gonçalo, K. Godse and L. F. Ensina (2021). "Managing Chronic Urticaria and Recurrent Angioedema Differently with Advancing Age." The Journal of Allergy and Clinical Immunology: In Practice 9(6): 2186-2194.	Title
274	Lorente Garín, J., D. Cañis Sánchez, O. Arango Toro, O. Bielsa Gali, R. Cortadellas Ángel and A. Gelabert Mas (2004). "Doxazosina en formulación de liberación retardada en la retención aguda de orina por hiperplasia benigna de próstata: Acción del incremento de dosis sobre el efecto rescate." Actas Urológicas Españolas 28(1): 32-37.	Language
275	Louis, W. J., J. J. McNeil, S. N. Anavekar, E. L. Conway, B. Workman, L. G. Howes, O. H. Drummer and B. Jarrott (1987). "Comparison of pharmacokinetics and pharmacodynamics of adrenoceptor agonists and antagonists as antihypertensive agents." J Cardiovasc Pharmacol 10 Suppl 12: S100-103.	Title
276	Lowe, F. C. (1997). "Coadministration of tamsulosin and three antihypertensive agents in patients with benign prostatic hyperplasia: pharmacodynamic effect." Clinical Therapeutics 19(4): 730-742.	Title
277	Lowe, F. C. (2004). "Role of the newer alpha, -adrenergic-receptor antagonistsin the treatment of benign prostatic hyperplasia-related lower urinary tract symptoms." Clinical Therapeutics 26(11): 1701-1713.	Title
278	Lowenthal, D., K. Matzek, T. MacGregor, R. Carlson, R. Bailey and E. Begg (1999). "ANTIHYPERTENSIVE DRUGS ADRENERGIC AND SEROTONINERGIC MODULATORS." Drug Prescribing in Renal Failure: Dosing Guidelines for Adults 49: 102.	Title
279	Luks, A. M. and E. R. Swenson (2008). "Medication and Dosage Considerations in the Prophylaxis and Treatment of High-Altitude Illness." Chest 133(3): 744-755.	Title
280	Lund-Johansen, P. (1975). "Hemodynamic changes at rest and during exercise in long-term prazosin therapy for essential hypertension." Postgraduate Medicine: 45-52.	Title
281	Lund-Johansen, P. and R. S. Kirby (2003). "Effect of doxazosin GITS on blood pressure in hypertensive and normotensive patients: a review of hypertension and BPH studies." Blood pressure 12(sup1): 5-13.	Review

Sr no.	Title	Reason
282	Maccagnano, C., A. Salonia, A. Briganti, P. Teillac, C. Schulman, F. Montorsi and P. Rigatti (2006). "A Critical Analysis of Permixon™ in the Treatment of Lower Urinary Tract Symptoms Due to Benign Prostatic Enlargement." European Urology Supplements 5(4): 430-440.	Title
283	Macphee, G. J., J. Curzio, E. Farish, J. L. Reid and H. L. Elliott (1992). "Placebo-controlled trial of doxazosin in management of patients with hypertension and hypercholesterolaemia." Journal of cardiovascular pharmacology 20(3): 429-433.	Abstract
284	Maheux, P., F. Facchini, J. Jeppesen, M. S. Greenfield, C. Clinkingbeard, Y. D. Chen and G. M. Reaven (1994). "Changes in glucose, insulin, lipid, lipoprotein, and apoprotein concentrations and insulin action in doxazosin-treated patients with hypertension. Comparison between nondiabetic individuals and patients with non-insulin-dependent diabetes mellitus." Am J Hypertens 7(5): 416-424.	Title
285	Malaterre, V., J. Ogorka, N. Loggia and R. Gurny (2009). "Oral osmotically driven systems: 30 years of development and clinical use." European Journal of Pharmaceutics and Biopharmaceutics 73(3): 311-323.	Title
286	Malki, M. A., A. Y. Dawed, C. Haywood, A. Doney and E. R. Pearson (2021). "Utilizing Large Electronic Medical Record Data Sets to Identify Novel Drug-Gene Interactions for Commonly Used Drugs." Clin Pharmacol Ther 110(3): 816-825.	Title
287	Man in't Veld, A. J. (1990). "How to select a drug for the long-term treatment of chronic heart failure." American Heart Journal 120(6, Part 2): 1572-1578.	Title
288	Mangray, M. and J. P. Vella (2011). "Hypertension After Kidney Transplant." American Journal of Kidney Diseases 57(2): 331-341.	Title
289	Mann, S. J. (2006). "Doxazosin gastrointestinal therapeutic system: A clinical perspective." The Journal of Clinical Hypertension 8(3): 207.	Abstract
290	Manoharan, A., A. E. Morrison and B. J. Lipworth (2016). "Effects of the inverse alpha-agonist doxazosin in allergic rhinitis." Clinical and experimental allergy 46(5): 696-704.	Title
291	Marin Iranzo, R., L. M. Ruilope, P. Aljama, P. Aranda and J. Diez Martinez (1995). "Effect of antihypertensive treatment on progression of renal insufficiency in non-diabetics patients. (ESPIRAL trial)." Nefrologia 15(5): 464-475.	Title
292	Martínez Castelao, A., M. Ibernón, X. Sarrias, V. Sanz, F. Moreso, I. Rama and J. M. Grinyó (2003). "Doxazosin GITS trough to peak ratio and 24-hour blood pressure monitoring in the management of hypertension in renal transplant patients." Transplant Proc 35(5): 1736-1738.	Abstract
293	Martinez Martin, F. (2010). "The effects of olmesartan/amlodipine on metabolic and inflammation mediators in hypertensive patients with metabolic syndrome are dose-dependent: the olas study." Journal of hypertension 28: e107-108.	Title

Sr no.	Title	Reason
294	Martínez-Castelao, A., M. Hueso, V. Sanz, J. Rejas, J. Sarrias, J. Alsina and J. M. Grinyó (2002). "Double-blind, crossover, comparative study of doxazosin and enalapril in the treatment of hypertension in renal transplant patients under cyclosporine immunosuppression." Transplantation proceedings 34(1): 403-406.	No Access
295	Martinez-Martin, F. J., H. Rodriguez-Rosas, I. Peiro-Martinez, P. Soriano-Perera, P. Pedrianes-Martin and C. Comi-Diaz (2011). "Olmesartan/amlodipine vs olmesartan/hydrochlorothiazide in hypertensive patients with metabolic syndrome: the OLAS study." Journal of human hypertension 25(6): 346-353.	Title
296	Marzabadi, M. R., H. Xingfang, D. Nagarathnam, M. ShouWu, G. Chiu, W. C. Wong, J. M. Wetzel, J. Fang, C. Forray, T. B. Chen, S. S. O'Malley, R. S. L. Chang and C. Gluchowski (1999). "Design and synthesis of novel dihydropyridine alpha-1A antagonists." Bioorganic & Medicinal Chemistry Letters 9(19): 2843-2848.	Title
297	Maxwell, A. P. (2002). "Novel erythropoiesis-stimulating protein in the management of the anemia of chronic renal failure." Kidney International 62(2): 720-729.	Title
298	Mazzola, C. and E. Guerrasio (1988). "Doxazosin versus atenolol: a randomized comparison of calculated coronary heart disease risk reduction." American Heart Journal 116(6): 1797-1801.	Title
299	McComb, M. N., J. Y. Chao and T. M. Ng (2016). "Direct vasodilators and sympatholytic agents." Journal of cardiovascular pharmacology and therapeutics 21(1): 3-19.	Title
300	McGrath, J. C., H. Lepor and M. G. Wyllie (1996). "Report of a unique meeting between the alpha-blocker subcommittee and the pharmaceutical industry." Urology 48(5): 665-667.	Title
301	McMurray, E. M., I. R. Wallace, C. Ennis, S. J. Hunter, A. B. Atkinson and P. M. Bell (2014). "Effect of eplerenone on insulin action in essential hypertension: a randomised, controlled, crossover study." Journal of human hypertension 28(10): 575-578.	Title
302	Meredith, P. (2000). "Pharmacokinetics of Doxazosin Gastrointestinal Therapeutic System (Dox Gits) Versus Doxazosin Standard: Potential Benefits of Dox Gits in the Treatment of Patients With Hypertension: P1. 57." Journal of Hypertension 18: S29.	No Access
303	Meredith, P. A. (2001). "Is postural hypotension a real problem with antihypertensive medication?" Cardiology 96(Suppl. 1): 19-24.	Title
304	Meredith, P. A., H. L. Elliott, R. Donnelly and J. L. Reid (1991). "Dose-response clarification in early drug development." J Hypertens Suppl 9(6): S356-357.	Title
305	Meredith, P. A., H. L. Elliott, A. W. Kelman and J. L. Reid (1985). "Application of pharmacokinetic-pharmacodynamic modelling for the comparison of quinazoline alpha-adrenoceptor agonists in normotensive volunteers." J Cardiovasc Pharmacol 7(3): 532-537.	Full text

Sr no.	Title	Reason
306	Meredith, P. A., H. L. Elliott, A. W. Kelman, J. Vincent and J. L. Reid (1988). "Pharmacokinetic and pharmacodynamic modelling of the alpha adrenoceptor antagonist doxazosin." Xenobiotica 18(1): 123-129.	Full text
307	Michel, M. C. (2002). "Potential Role of α1-Adrenoceptors in the Aetiology of LUTS." European Urology Supplements 1(9): 5-13.	Title
308	Michel, M. C. (2010). "The forefront for novel therapeutic agents based on the pathophysiology of lower urinary tract dysfunction: alpha-blockers in the treatment of male voiding dysfunction - how do they work and why do they differ in tolerability?" J Pharmacol Sci 112(2): 151-157.	Title
309	Michel, M. C. (2010). "The Forefront for Novel Therapeutic Agents Based on the Pathophysiology of Lower Urinary Tract Dysfunction: α-Blockers in the Treatment of Male Voiding Dysfunction — How Do They Work and Why Do They Differ in Tolerability?" Journal of Pharmacological Sciences 112(2): 151-157.	Title
310	Michel, M. C. (2010). "The Pharmacological Profile of the α1A-Adrenoceptor Antagonist Silodosin." European Urology Supplements 9(4): 486-490.	Title
311	Michel, M. C. and C. R. Chapple (2006). "Comparison of the Cardiovascular Effects of Tamsulosin Oral Controlled Absorption System (OCAS®) and Alfuzosin Prolonged Release (XL)." European Urology 49(3): 501-509.	Title
312	Michel, M. C., C. Korstanje and W. Krauwinkel (2005). "Cardiovascular Safety of Tamsulosin Modified Release in the Fasted and Fed State in Elderly Healthy Subjects." European Urology Supplements 4(2): 9-14.	Title
313	Michel, M. C., C. Korstanje, W. Krauwinkel and M. Kuipers (2005). "The pharmacokinetic profile of tamsulosin oral controlled absorption system (OCAS®)." European Urology Supplements 4(2): 15-24.	Title
314	Michel, M. C., C. Korstanje, W. Krauwinkel, M. Shear, J. Davies and A. Quartel (2005). "Cardiovascular Safety of the Oral Controlled Absorption System (OCAS) Formulation of Tamsulosin Compared to the Modified Release (MR) Formulation." European Urology Supplements 4(2): 53-60.	Title
315	Michel, M. C., C. Korstanje, W. Krauwinkel, M. Shear, J. Davies and A. Quartel (2005). "Comparison of Vascular α1-Adrenoceptor Antagonism of Tamsulosin in Oral Controlled Absorption System (OCAS) and Modified Release (MR) Formulations." European Urology Supplements 4(2): 45-52.	Title
316	Michel, M. C., K. Taguchi, R. S. Schäfers, T. J. Williams, D. E. Clarke and A. P. D. W. Ford (1997). α1-Adrenoceptor Subtypes in the Human Carondiovascular and Urogenital Systems. Advances in Pharmacology. D. S. Goldstein, G. Eisenhofer and R. McCarty, Academic Press. 42: 394-398.	Title

Sr no.	Title	Reason
317	Mills, C. E., V. Govoni, L. Faconti, M. L. Casagrande, S. V. Morant, H. Crickmore, F. Iqbal, P. Maskell, A. Masani, E. Nanino, A. J. Webb and J. K. Cruickshank (2020). "A randomised, factorial trial to reduce arterial stiffness independently of blood pressure: Proof of concept? The VaSera trial testing dietary nitrate and spironolactone." Br J Clin Pharmacol 86(5): 891-902.	Title
318	Miura, Y. and K. Yoshinaga (1988). "Doxazosin: a newly developed, selective alpha 1-inhibitor in the management of patients with pheochromocytoma." American heart journal 116(6 Pt 2): 1785-1789.	Abstract
319	Miyazawa, Y., R. A. Blum, J. J. Schentag, H. Kamimura, H. Matsushima, H. Swarz and Y. Ito (2001). "Pharmacokinetics and safety of tamsulosin in subjects with normal and impaired renal or hepatic function." Current Therapeutic Research 62(9): 603-621.	Title
320	Moda, T. L., C. A. Montanari and A. D. Andricopulo (2007). "Hologram QSAR model for the prediction of human oral bioavailability." Bioorganic & Medicinal Chemistry 15(24): 7738-7745.	Title
321	Mondaini, N., G. Giubilei, A. Ungar, P. Gontero, T. Cai, A. Gavazzi, R. Bartoletti, P. Geppetti and M. Carini (2006). "Alfuzosin (10mg) Does Not Affect Blood Pressure in Young Healthy Men." European Urology 50(6): 1292-1298.	Title
322	Montorsi, F., L. Kuritzky, R. Sadovsky, P. Fredlund and W. H. Cordell (2005). "Frequently asked questions about tadalafil for treating men with erectile dysfunction." The Journal of Men's Health & Gender 2(1): 141-157.	Title
323	Mora-Maciá, J., J. A. Jover and J. S. García (2003). "Tratamiento del paciente hipertenso con hiperplasia benigna de próstata." Hipertensión y Riesgo Vascular 20(9): 395-402.	Title
324	Mori, Y., H. Matsubara, A. Nose, Y. Shibasaki, H. Masaki, A. Kosaki, M. Okigaki, S. Fujiyama, Y. Tanaka-Uchiyama, T. Hasegawa, O. Iba, E. Tateishi, K. Amano and T. Iwasaka (2001). "Safety and availability of doxazosin in treating hypertensive patients with chronic renal failure." Hypertens Res 24(4): 359-363.	Abstract
325	Motomura, N., Y. Yamazaki, X. Gao, Y. Tezuka, K. Omata, Y. Ono, R. Morimoto, F. Satoh, Y. Nakamura, J. Shim, M. H. Choi, A. Ito and H. Sasano (2022). "Visualization of calcium channel blockers in human adrenal tissues and their possible effects on steroidogenesis in the patients with primary aldosteronism (PA)." The Journal of Steroid Biochemistry and Molecular Biology 218: 106062.	Title
326	Mu¨ller, M., R. J. Litz, M. Hu¨bler and D. M. Albrecht (2001). "Grand mal convulsion and plasma concentrations after intravascular injection of ropivacaine for axillary brachial plexus blockade." British Journal of Anaesthesia 87(5): 784-787.	Title

Sr no.	Title	Reason
327	Mukhtarov, S., A. Turdiev, A. Fozilov, D. Arustamov and B. Ayubov (2007). "Using doxazosin for distal ureteral stone clearance with or without shock wave lithotripsy." European urology supplements 6(2): 216.	Title
328	Musther, H., A. Olivares-Morales, O. J. D. Hatley, B. Liu and A. Rostami Hodjegan (2014). "Animal versus human oral drug bioavailability: Do they correlate?" European Journal of Pharmaceutical Sciences 57: 280-291.	Title
329	Naidu, N., M. R. M. Reddy and P. Suguna (2012). "Development and validation of the HPLC method for the analysis of Doxazosin in bulk drug and pharmaceutical dosage forms." International Journal of Pharmaceutical Sciences and Research 3(8): 2705.	Abstract
330	Nanjee, M. N. and N. E. Miller (1987). "Studies with doxazosin on the saturable binding of 125I-LDL by liver in normocholesterolemic mice." J Cardiovasc Pharmacol 10 Suppl 9: S35-41.	Title
331	Nazarudheen, S., S. Dey, K. Kandhwal, R. Arora, S. Reyar, A. H. Khuroo, T. Monif, S. Madan and V. Arora (2013). "Combining benefits of an adrenergic and a muscarinic blocker in a single formulation – A pharmacokinetic evaluation." Regulatory Toxicology and Pharmacology 67(2): 226-231.	Title
332	Nct (1999). "Antihypertensive and Lipid-Lowering Treatment to Prevent Heart Attack Trial (ALLHAT)." https://clinicaltrials.gov/show/NCT00000542.	Title
333	Nct (2005). "Atorvastatin and Endothelial Function in Type 2 Diabetes Mellitus (ATTEND-Study)." https://clinicaltrials.gov/show/NCT00124397.	Title
334	Nct (2006). "Pharmacosurveillance and Pharmacogenetics of First-line Diuretics in Hypertension: the StayOnDiur Study." https://clinicaltrials.gov/show/NCT00408512.	Title
335	Nct (2008). "A Study to Investigate the Effects on Blood Pressure and Pulse Rate of a Single 100mg Dose of Sildenafil in Patients With Benign Prostatic Hyperplasia Who Are Being Treated With Doxazosin." https://clinicaltrials.gov/show/NCT00645034.	Title
336	Nct (2010). "The Effects of Doxazosin on the Cardiovascular and Subjective Effects of Cocaine." https://clinicaltrials.gov/show/NCT01062945.	Title
337	Nct (2011). "A Pharmacodynamic Study of Dapoxetine Concomitantly Administered in Participants Taking Terazosin." https://clinicaltrials.gov/show/NCT01366664.	Title
338	Nct (2011). "Pharmacological Interaction Between Doxazosin and Methylenedioxymethamphetamine (MDMA)." https://clinicaltrials.gov/show/NCT01386177.	Title
339	Nct (2011). "Phenoxybenzamine Versus Doxazosin in PCC Patients." https://clinicaltrials.gov/show/NCT01379898.	Title
340	Nct (2015). "CAP: doxazosin in the Treatment of Co-Occurring PTSD and Alcohol Use Disorders." https://clinicaltrials.gov/show/NCT02500602.	Title

Sr no.	Title	Reason
341	Nct (2020). "Influence of the Autonomic Nervous System in Response to Exercise in Hypertensive Individuals." https://clinicaltrials.gov/show/NCT04371757.	Title
342	Nct (2022). "Composite Steep-pulseTreatment Device Used in Patients With Benign Prostatic Hyperplasia." https://clinicaltrials.gov/show/NCT05531344.	Title
343	Neaton, J. D., R. H. Grimm, R. J. Prineas, J. Stamler, G. A. Grandits, P. J. Elmer, J. A. Cutler, J. M. Flack, J. A. Schoenberger and R. McDonald (1993). "Treatment of Mild Hypertension Study. Final results. Treatment of Mild Hypertension Study Research Group." JAMA 270(6): 713-724.	Title
344	Ng, C.-F., A. Wong, C.-W. Cheng, E. S.-Y. Chan, H.-M. Wong and S.-M. Hou (2008). "Effect of vardenafil on blood pressure profile of patients with erectile dysfunction concomitantly treated with doxazosin gastrointestinal therapeutic system for benign prostatic hyperplasia." The Journal of urology 180(3): 1042-1046.	Title
345	Nguyen, L. H., E. Bruyn, R. Webster, A. Murphy, P. Perel and A. E. Schutte (2022). "Are We There Yet? Exploring the Use of Single-Pill Combination Therapy in the Management of Raised Blood Pressure in Australia." Heart, Lung and Circulation 31(7): 954-963.	Title
346	Ni, Y., Y. Zhou, M. Xu, X. He, H. Li, S. Haseeb, H. Chen and W. Li (2015). "Simultaneous determination of phentermine and topiramate in human plasma by liquid chromatography-tandem mass spectrometry with positive/negative ion-switching electrospray ionization and its application in pharmacokinetic study." J Pharm Biomed Anal 107: 444-449.	Title
347	Nomura, A., S. Kakinoki, M. Sakurai, T. Hirabayashi, M. Nakamura, K. Yoneya, K. Imamura and A. Kitabatake (1996). "Effects of doxazosin on orthostatic blood pressure in hypertensive patients with and without diabetes mellitus." Current Therapeutic Research 57(6): 411-417.	Title
348	Nowicki, M., T. Waluś and F. Kokot (2002). "Influence of the alpha-1-adrenergic receptor blocker doxazosin on exercise-induced hyperkalemia in hemodialysis patients." Kidney Blood Press Res 25(1): 55-60.	Abstract
349	Ocharán-Corcuera, J., I. Iribar, R. Saracho, I. Martínez and J. Montenegro (2002). Doxazosina, de liberación modificada, en pacientes de hemodiálisis. Anales de Medicina Interna, SciELO Espana.	Language
350	Oelke, M., K. Hofner, R. R. Berges and U. Jonas (2002). "Pharmacological treatment of the benign prostatic syndrome (symptomatic BPH) using alpha1-adrenoceptor antagonists. Basic principles and clinical results." Urologe - ausgabe a 41(5): 425-441.	Title
351	Oesterling, J. E. (1994). "Endocrine therapies for symptomatic benign prostatic hyperplasia." Urology 43: 7-16.	Title

Sr no.	Title	Reason
352	Oestreich, M. C., R. W. M. Vernooij, N. J. Sathianathen, E. C. Hwang, G. M. Kuntz, A. Koziarz, C. D. Scales and P. Dahm (2020). "Alpha-blockers after shock wave lithotripsy for renal or ureteral stones in adults." Cochrane Database of Systematic Reviews(11).	Title
353	Offerhaus, L. (1991). β-adrenoceptor antagonists and antianginal drugs. Side Effects of Drugs Annual. M. N. G. Dukes and J. K. Aronson, Elsevier. 15: 188-197.	Title
354	Oger, S., D. Behr-Roussel, D. Gorny, O. Lecoz, T. Lebret, Y. Denoux, A. Faix, A. Leriche, C. Wayman, L. Alexandre and et al. (2009). "Combination of doxazosin and sildenafil exerts an additive relaxing effect compared with each compound alone on human cavernosal and prostatic tissue." Journal of sexual medicine 6(3): 836-847.	Title
355	Olaku, O., B. A. Conley, S. P. Ivy, L. M. McShane, L. M. Staudt, S. M. King, M. Sansevere, B. Kim and J. D. White (2022). "Survey of Lifestyle, Past Medical History and Complementary and Alternative Medicine Use Among Adult Patients Participating in the National Cancer Institute's Exceptional Responders Initiative." Translational Oncology 25: 101484.	Title
356	Oliveros-Palacios, M. C., N. Godoy-Godoy and J. A. Colina-Chourio (1991). "Effects of doxazosin on blood pressure, renin-angiotensin-aldosterone and urinary kallikrein." American journal of cardiology 67(2): 157-161.	Title
357	Omar, M. A., M. A. Hammad and B. I. Salman (2018). "Micellar enhanced spectrofluorimetric approach for nanogram detection of certain α(1) -blocker drugs: Application in pharmaceutical preparations and human plasma." Luminescence 33(7): 1226-1234.	Title
358	Omar, M. A., M. A. Hammad, B. I. Salman and S. M. Derayea (2016). "Highly sensitive spectrofluorimetric method for determination of doxazosin through derivatization with fluorescamine; Application to content uniformity testing." Spectrochim Acta A Mol Biomol Spectrosc 157: 55-60.	Abstract
359	Omar, M. A., A. A. Mohamed, A.-M. I. Mohamed, S. M. Derayea and M. A. Hammad (2020). "Mending the fluorescence of two α-blockers through a semiquinoid formation: Hyphenated with experimental design optimization." Spectrochimica Acta Part A: Molecular and Biomolecular Spectroscopy 240: 118624.	Title
360	Omar, M. A., A. I. Mohamed, S. M. Derayea, M. A. Hammad and A. A. Mohamed (2018). "An efficient spectrofluorimetric method adopts doxazosin, terazosin and alfuzosin coupling with orthophthalaldehyde: Application in human plasma." Spectrochim Acta A Mol Biomol Spectrosc 195: 215-222.	Full text
361	O'Neil, M. L., L. E. Beckwith, C. L. Kincaid and D. D. Rasmussen (2013). "The α1-adrenergic receptor antagonist, doxazosin, reduces alcohol drinking in alcohol-preferring (P) Rats." Alcohol Clin Exp Res 37(2): 202-212.	Title

Sr no.	Title	Reason
362	Opie, L. H. (2000). "Adverse cardiovascular drug interactions." Current Problems in Cardiology 25(9): 621-676.	Title
363	Os, I. (2006). "Comparison of doxazosin GITS and standard doxazosin in the treatment of high blood pressure." International Journal of Clinical Pharmacology & Therapeutics 44(3).	Title
364	Os, I. and H. P. Stokke (1999). "Doxazosin GITS compared with doxazosin standard and placebo in patients with mild hypertension." Blood Press 8(3): 184-191.	Abstract
365	Ozkan, S. A. and B. Uslu (2016). "From mercury to nanosensors: Past, present and the future perspective of electrochemistry in pharmaceutical and biomedical analysis." Journal of Pharmaceutical and Biomedical Analysis 130: 126-140.	Title
366	Pallesen, S., H. S. Hamre, N. Lang and B. Bjorvatn (2020). "Doxazosin for the treatment of nightmare disorder: A diary-based case study." SAGE Open Medical Case Reports 8: 2050313X20936079.	Title
367	Pani, L. and G. Marchese (2009). "Expected clinical benefits of paliperidone extended-release formulation when compared with risperidone immediate-release." Expert Opinion on Drug Delivery 6(3): 319-331.	Title
368	Papadakis, J. A., E. S. Ganotakis, I. A. Jagroop, D. P. Mikhailidis and A. F. Winder (1999). "Effect of hypertension and its treatment on lipid, lipoprotein(a), fibrinogen, and bilirubin levels in patients referred for dyslipidemia." Am J Hypertens 12(7): 673-681.	Title
369	Papademetriou, V., L. B. Piller, C. E. Ford, D. Gordon, T. J. Hartney, T. S. Geraci, E. Reisin, B. M. Sumner, N. D. Wong, C. Nwachuku and et al. (2003). "Characteristics and lipid distribution of a large, high-risk, hypertensive population: the lipid-lowering component of the Antihypertensive and Lipid-Lowering Treatment to Prevent Heart Attack Trial (ALLHAT)." Journal of clinical hypertension (Greenwich, Conn.) 5(6): 377-384.	Title
370	Paśko, P., T. Rodacki, R. Domagała-Rodacka and D. Owczarek (2016). "Interactions between medications employed in treating benign prostatic hyperplasia and food - A short review." Biomed Pharmacother 83: 1141-1145.	Title
371	Paśko, P., T. Rodacki, R. Domagała-Rodacka and D. Owczarek (2016). "Interactions between medications employed in treating benign prostatic hyperplasia and food− A short review." Biomedicine & Pharmacotherapy 83: 1141-1145.	Title
372	Pasquali, S. K., S. P. Sanders and J. S. Li (2002). "Oral antihypertensive trial design and analysis under the pediatric exclusivity provision." American Heart Journal 144(4): 608-614.	Title

Sr no.	Title	Reason
373	Pérez, M. A. C., M. B. Sanz, L. R. Torres, R. G. Ávalos, M. P. González and H. G. Díaz (2004). "A topological sub-structural approach for predicting human intestinal absorption of drugs." European Journal of Medicinal Chemistry 39(11): 905-916.	Title
374	Pessina, A. C., L. Ciccariello, F. Perrone, V. Stoico, G. Gussoni, A. Scotti and M. Muggeo (2006). "Clinical efficacy and tolerability of alpha-blocker doxazosin as add-on therapy in patients with hypertension and impaired glucose metabolism." Nutr Metab Cardiovasc Dis 16(2): 137-147.	Abstract
375	Pickering, T. G., M. Levenstein and P. Walmsley (1994). "Nighttime dosing of doxazosin has peak effect on morning ambulatory blood pressure. Results of the HALT Study. Hypertension and Lipid Trial Study Group." Am J Hypertens 7(9 Pt 1): 844-847.	Title
376	Planche, T. and S. Krishna (2002). Antiprotozoal drugs. Side Effects of Drugs Annual. J. K. Aronson, Elsevier. 25: 343-352.	Title
377	Podrazik, P. M. and J. B. Schwartz (1999). "CARDIOVASCULAR PHARMACOLOGY OF AGING." Cardiology Clinics 17(1): 17-34.	Title
378	Pomara, G., G. Morelli, S. Pomara, S. Taddei, L. Ghiadoni, N. Dinelli, F. Travaglini, M. Dicuio, N. Mondaini, A. Salvetti and et al. (2004). "Cardiovascular parameter changes in patients with erectile dysfunction using pde-5 inhibitors: a study with sildenafil and vardenafil." Journal of andrology 25(4): 625-629.	Title
379	Pool, J. L. and R. S. Kirby (2001). "Clinical significance of alpha1-adrenoceptor selectivity in the management of benign prostatic hyperplasia." Int Urol Nephrol 33(3): 407-412.	Title
380	Porst, H., A. Burnett, G. Brock, H. Ghanem, F. Giuliano, S. Glina, W. Hellstrom, A. Martin-Morales, A. Salonia and I. Sharlip (2013). "SOP Conservative (Medical and Mechanical) Treatment of Erectile Dysfunction." The Journal of Sexual Medicine 10(1): 130-171.	Title
381	Postuła, M. (2023). "Doksazosyna w postaci doustnego systemu terapeutycznego u pacjenta z nadciśnieniem tętniczym." Medycyna Faktów 16(1 (58)): 107-110.	Language
382	Prichard, B. N. C., B. Tomlinson and J.-C. Renondin (1989). "Urapidil, a multiple-action alpha-blocking drug." The American Journal of Cardiology 64(7): D11-D15.	Title
383	Prisant, L. M. and W. J. Elliott (2003). "Drug delivery systems for treatment of systemic hypertension." Clinical pharmacokinetics 42: 931-940.	Title
384	Prys-Roberts, C. (1994). "Structure and function of adrenoceptors." Baillière's Clinical Anaesthesiology 8(1): 1-25.	Title
385	Punt, A., N. Stienstra, M. van Kleef, M. Lafeber, W. Spiering, P. Blankestijn, M. Bots and E. van Maarseveen (2019). "Screening of cardiovascular agents in plasma with LC-	Title

Sr no.	Title	Reason
	MS/MS: a valuable tool for objective drug adherence assessment." Journal of Chromatography B 1121: 103-110.	
386	Pupe, C. G., F. A. Do Carmo, V. P. De Sousa, M. Lopes, B. Abrahim-Vieira, A. J. Ribeiro, F. Veiga, C. R. Rodrigues, C. Padula, P. Santi and L. M. Cabral (2013). "Development of a doxazosin and finasteride transdermal system for combination therapy of benign prostatic hyperplasia." J Pharm Sci 102(11): 4057-4064.	Abstract
387	Puspita, D., B. Chandra and H. Rivai (2021). "Overview of Determination of Doxazosin Levels in Pharmaceutical Preparations and Biological Matrix."	Title
388	Quaresma, B., A. R. Pimenta, A. C. Santos da Silva, A. S. Pupo, L. A. S. Romeiro, C. L. M. Silva and F. Noël (2019). "Revisiting the Pharmacodynamic Uroselectivity of α (1)-Adrenergic Receptor Antagonists." J Pharmacol Exp Ther 371(1): 106-112.	Title
389	Rabelo, V. W.-H., D. d. J. Viegas, E. M. N. Tucci, N. C. Romeiro and P. A. Abreu (2019). "Virtual screening and drug repositioning as strategies for the discovery of new antifungal inhibitors of oxidosqualene cyclase." The Journal of Steroid Biochemistry and Molecular Biology 185: 189-199.	Title
390	Rabkin, S. W., M. W. Huff, C. Newman, D. Sim and S. G. Carruthers (1994). "Lipids and lipoproteins during antihypertensive drug therapy. Comparison of doxazosin and atenolol in a randomized, double-blind trial: the Alpha Beta Canada Study." Hypertension 24(2): 241-248.	Title
391	Rachima-Maoz, C., E. Peleg and T. Rosenthal (1998). "The effect of caffeine on ambulatory blood pressure in hypertensive patients." American Journal of Hypertension 11(12): 1426-1432.	Title
392	Rachmani, R., Z. Levi, I. Slavachevsky, E. Half-Onn and M. Ravid (1998). "Effect of an alpha-adrenergic blocker, and ACE inhibitor and hydrochlorothiazide on blood pressure and on renal function in type 2 diabetic patients with hypertension and albuminuria. A randomized cross-over study." Nephron 80(2): 175-182.	Title
393	Radchenko, G. D., Y. M. Sirenko, S. M. Kushnir, O. O. Torbas and A. S. Dobrokhod (2013). "Comparative effectiveness of a fixed-dose combination of losartan + HCTZ versus bisoprolol + HCTZ in patients with moderate-to-severe hypertension: results of the 6-month ELIZA trial." Vascular health and risk management 9: 535-549.	Title
394	Raggi, M. A., R. Mandrioli, G. Casamenti, V. Volterra and S. Pinzauti (2002). "Determination of reboxetine, a recent antidepressant drug, in human plasma by means of two high-performance liquid chromatography methods." Journal of Chromatography A 949(1): 23-33.	Title

Sr no.	Title	Reason
395	Raymond, J. L. and C. S. Smith (1997). "Trends in alpha-blocker treatment of patients with benign prostatic hyperplasia and hypertension: dosing regimens and cost comparisons." Clinical Therapeutics 19(4): 821-829.	Title
396	Reid, J. L. (1986). "Alpha-adrenergic receptors and blood pressure control." The American Journal of Cardiology 57(9): E6-E12.	Title
397	Reid, J. L., H. L. Elliott, J. Vincent and P. A. Meredith (1987). "Clinical pharmacology of selective alpha blockers: Hemodynamics and effects on lipid levels." The American Journal of Medicine 82(1, Supplement 1): 15-20.	Title
398	Reid, J. L., P. A. Meredith and H. L. Elliott (1983). "Pharmacokinetics and pharmacodynamics of trimazosin in man." American Heart Journal 106(5, Part 2): 1222-1228.	Title
399	Reid, J. L. and J. Vincent (1986). "Clinical pharmacology and therapeutic role of prazosin and related alpha-adrenoceptor antagonists." Cardiology 73(3): 164-174.	Title
400	Richelson, E. (1993). "Treatment of Acute Depression." Psychiatric Clinics of North America 16(3): 461-478.	Title
401	Richter, L. H. J., C. M. Jacobs, F. Mahfoud, I. Kindermann, M. Böhm and M. R. Meyer (2019). "Development and application of a LC-HRMS/MS method for analyzing antihypertensive drugs in oral fluid for monitoring drug adherence." Analytica Chimica Acta 1070: 69-79.	Title
402	Ring, C., L. K. Harrison, A. Winzer, D. Carroll, M. Drayson and M. Kendall (2000). "Secretory immunoglobulin A and cardiovascular reactions to mental arithmetic, cold pressor, and exercise: effects of alpha-adrenergic blockade." Psychophysiology 37(5): 634-643.	Title
403	Rodgman, C., C. D. Verrico, M. Holst, D. Thompson-Lake, C. N. Haile, R. De La Garza, 2nd, M. A. Raskind and T. F. Newton (2016). "Doxazosin XL reduces symptoms of posttraumatic stress disorder in veterans with PTSD: a pilot clinical trial." J Clin Psychiatry 77(5): e561-565.	Title
404	Roehrborn, C. G. (2001). "Alfuzosin: overview of pharmacokinetics, safety, and efficacy of a clinically uroselective α-blocker." Urology 58(6, Supplement 1): 55-63.	Title
405	Roehrborn, C. G. (2001). "Efficacy and safety of once-daily alfuzosin in the treatment of lower urinary tract symptoms and clinical benign prostatic hyperplasia: a randomized, placebo-controlled trial11The members of the ALFUS Study Group are given in the Appendix." Urology 58(6): 953-959.	Title
406	Roehrborn, C. G., A. Prajsner, R. Kirby, M. Andersen, S. Quinn and S. Mallen (2005). "A double-blind placebo-controlled study evaluating the onset of action of doxazosin	Title

Sr no.	Title	Reason
	gastrointestinal therapeutic system in the treatment of benign prostatic hyperplasia." European urology 48(3): 445-452.	
407	Roehrborn, C. G. and D. A. Schwinn (2004). "Alpha1-adrenergic receptors and their inhibitors in lower urinary tract symptoms and benign prostatic hyperplasia." J Urol 171(3): 1029-1035.	Review
408	Rosen, R. C., J. A. Catania, A. A. Ehrhardt, A. L. Burnett, T. F. Lue, K. McKenna, J. R. Heiman, S. Schwarcz, D. G. Ostrow, S. Hirshfield, D. W. Purcell, W. A. Fisher, R. Stall, P. N. Halkitis, D. M. Latini, J. Elford, E. O. Laumann, F. L. Sonenstein, D. J. Greenblatt, R. A. Kloner, J. Lee, D. Malebranche, E. Janssen, R. Diaz, J. D. Klausner, A. L. Caplan, G. Jackson, R. Shabsigh, J. H. Khalsa and D. M. Stoff (2006). "REPORTS: The Bolger Conference on PDE-5 Inhibition and HIV Risk: Implications for Health Policy and Prevention." The Journal of Sexual Medicine 3(6): 960-975.	Title
409	Rosendorff, C., D. T. Lackland, M. Allison, W. S. Aronow, H. R. Black, R. S. Blumenthal, C. P. Cannon, J. A. de Lemos, W. J. Elliott, L. Findeiss, B. J. Gersh, J. M. Gore, D. Levy, J. B. Long, C. M. O'Connor, P. T. O'Gara, O. Ogedegbe, S. Oparil and W. B. White (2015). "Treatment of hypertension in patients with coronary artery disease: A scientific statement from the American Heart Association, American College of Cardiology, and American Society of Hypertension." Journal of the American Society of Hypertension 9(6): 453-498.	Title
410	Saghir, S. A., R. A. Ansari and M. A. Dorato (2020). "Rethinking toxicity testing: Influence of aging on the outcome of long-term toxicity testing and possible remediation." Food and Chemical Toxicology 141: 111327.	Title
411	Saghir, S. A., M. J. Bartels, D. L. Rick, A. T. McCoy, R. J. Rasoulpour, R. G. Ellis-Hutchings, M. Sue Marty, C. Terry, J. P. Bailey, R. Billington and J. S. Bus (2012). "Assessment of diurnal systemic dose of agrochemicals in regulatory toxicity testing – An integrated approach without additional animal use." Regulatory Toxicology and Pharmacology 63(2): 321-332.	Title
412	Saghir, S. A., A. L. Mendrala, M. J. Bartels, S. J. Day, S. C. Hansen, J. M. Sushynski and J. S. Bus (2006). "Strategies to assess systemic exposure of chemicals in subchronic/chronic diet and drinking water studies." Toxicology and Applied Pharmacology 211(3): 245-260.	Title
413	Sakauchi, N., H. Furukawa, J. Shirai, A. Sato, H. Kuno, R. Saikawa and M. Yoshida (2017). "Identification of 3,4-dihydro-2H-thiochromene 1,1-dioxide derivatives with a phenoxyethylamine group as highly potent and selective α1D adrenoceptor antagonists." European Journal of Medicinal Chemistry 139: 114-127.	Title

Sr no.	Title	Reason
414	Salonen, M. A., J. H. Kanto and M. Maze (1992). "Clinical interactions with alpha-2-adrenergic agonists in anesthetic practice." Journal of Clinical Anesthesia 4(2): 164-172.	Title
415	Sankar, R. and S. K. Jain (2013). "Determination of Target In-Vitro Drug Release Profile for Extended Release Formulation of Acyclovir through Pharmacokinetic Simulations." Anti-Infective Agents 11(2): 204-211.	Title
416	Sarkar, M., A. Nath, A. Kumer, C. Mallik, F. Akter, M. Moniruzzaman and M. A. Ali (2021). "Synthesis, molecular docking screening, ADMET and dynamics studies of synthesized 4-(4-methoxyphenyl)-8-methyl-3,4,5,6,7,8-hexahydroquinazolin-2(1H)-one and quinazolinone derivatives." Journal of Molecular Structure 1244: 130953.	Title
417	Sathianathen, N. J., Y. Fan, S. L. Jarosek, N. L. Lawrentschuk and B. R. Konety (2018). "Finasteride does not prevent bladder cancer: a secondary analysis of the Medical Therapy for Prostatic Symptoms Study." Urologic oncology 36(7): 338.e313-338.e317.	Title
418	Savva, M. (2022). "The Presence of Phases and the Inability of the Classical Compartment Models to Provide Pharmacokinetic Parameters of Physiological Significance for Lipophilic Drugs." Journal of Biosciences and Medicines 10(4): 1-13.	Title
419	Schäfers, R. F., J. Nürnberger, B. Herrmann, R. R. Wenzel, T. Philipp and M. C. Michel (1999). "Adrenoceptors mediating the cardiovascular and metabolic effects of alpha-methylnoradrenaline in humans." Journal of pharmacology and experimental therapeutics 289(2): 918-925.	Title
420	Schäfers, R. F., U. Poller, K. Pönicke, M. Geissler, A. E. Daul, M. C. Michel and O. E. Brodde (1997). "Influence of adrenoceptor and muscarinic receptor blockade on the cardiovascular effects of exogenous noradrenaline and of endogenous noradrenaline released by infused tyramine." Naunyn-Schmiedeberg's archives of pharmacology 355(2): 239-249.	Title
421	Schilit, S. and K. E. Benzeroual (2009). "Silodosin: A selective α1A-adrenergic receptor antagonist for the treatment of benign prostatic hyperplasia." Clinical Therapeutics 31(11): 2489-2502.	Title
422	Schwinn, D. A. and N. A. Afshari (2006). "α1-Adrenergic Receptor Antagonists and the Iris: New Mechanistic Insights into Floppy Iris Syndrome." Survey of Ophthalmology 51(5): 501-512.	Title
423	Scott, P. J. W. (1990). Studies on the Effects of Ageing on the Physiology and Pharmacology of the Autonomic Nervous System, University of Glasgow (United Kingdom).	Title
424	Seedat, Y. K. and I. P. Naiker (1997). "A single-masked study comparing doxazosin and enalapril in patients with non-insulin-dependent diabetes mellitus and hypertension." Current therapeutic research - clinical and experimental 58(9): 633-652.	Title

Sr no.	Title	Reason
425	Seljeflot, I., H. Arnesen, P. Andersen, T. Aspelin and P. Kierulf (1999). "Effects of doxazosin and atenolol on circulating endothelin-1 and von Willebrand factor in hypertensive middle-aged men." Journal of cardiovascular pharmacology 34(4): 584-588.	Title
426	Sengel-Turk, C. T. and C. Hascicek (2017). "Design of lipid-polymer hybrid nanoparticles for therapy of BPH: Part I. Formulation optimization using a design of experiment approach." Journal of Drug Delivery Science and Technology 39: 16-27.	Title
427	Sequeira, R. P. (2000). Central nervous system stimulants and drugs that suppress appetite. Side Effects of Drugs Annual. J. K. Aronson, Elsevier. 23: 1-14.	Title
428	Sethi, R. and S. Vasudeva (2012). "Doxazosin for the treatment of nightmares: does it really work? A case report." The Primary Care Companion for CNS Disorders 14(5): 26269.	Title
429	SH, H. K. L. S. S. and W. W. Wi "The Pan Mersey Area Prescribing Committee does not recommend the prescribing of DOXAZOSIN modified release (M/R) tablets (All Brands). BLACK."	Title
430	Shahu, A., J. Herrin, S. S. Dhruva, N. R. Desai, H. M. Krumholz and E. S. Spatz (2017). "Association of socioeconomic context with blood pressure response and cardiovascular outcomes in allhat." Hypertension 70.	Title
431	Shepherd, J. E. (2002). "Therapeutic Options in Female Sexual Dysfunction." Journal of the American Pharmaceutical Association (1996) 42(3): 479-488.	Title
432	Shieh, S. M., W. H. Sheu, D. C. Shen, M. M. Fuh, Y. D. Chen and G. M. Reaven (1992). "Glucose, insulin, and lipid metabolism in doxazosin-treated patients with hypertension." Am J Hypertens 5(11): 827-831.	Title
433	Shionoiri, H., K. Ashino, K. Yamanaka, K. Shindo, S. Hiroto and T. Arita (1997). "Effect of doxazosin therapy on glucose tolerance and lipid metabolism in hypertensive patients with impaired glucose tolerance." Clinical therapeutics 19(3): 527-536.	Abstract
434	Shreeve, S. M. (1990). "Effect of detergent solubilization on the affinity of some quinazoline derivatives for the α1-adrenoceptor." European Journal of Pharmacology: Molecular Pharmacology 188(1): 71-80.	Title
435	Sica, D. A., B. Carter, W. Cushman and L. Hamm (2011). "Thiazide and loop diuretics." J Clin Hypertens (Greenwich) 13(9): 639-643.	Title
436	Siegemund, M. (1997). "Oral anti-hypertensive drugs and anaesthesia." Baillière's Clinical Anaesthesiology 11(4): 731-757.	Title
437	Silva, H., R. Fonseca and D. Marshall (1991). "Doxazosin in the treatment of essential hypertension in general medical practice in Latin America." American Heart Journal 121(1): 329-335.	Title

Sr no.	Title	Reason
438	Silva, R. O., A. S. de Oliveira, L. F. Nunes Lemes, L. de Camargo Nascente, P. Coelho do Nascimento Nogueira, E. R. Silveira, G. D. Brand, G. Vistoli, A. Cilia, E. Poggesi, M. Buccioni, G. Marucci, M. L. Bolognesi and L. A. S. Romeiro (2016). "Synthesis and structure–activity relationships of novel arylpiperazines as potent antagonists of α1-adrenoceptor." European Journal of Medicinal Chemistry 122: 601-610.	Title
439	Simões Paço, J. and B. Jorge Pereira (2016). "New Therapeutic Perspectives in Premature Ejaculation." Urology 88: 87-92.	Title
440	Sinaiko, A. R. and S. R. Daniels (2001). "The use of short-acting nifedipine in children with hypertension: Another example of the need for comprehensive drug testing in children." The Journal of Pediatrics 139(1): 7-9.	Title
441	Sivarajah, A., S. Collins, M. R. Sutton, N. Regan, H. West, M. Holbrook and N. Edmunds (2010). "Cardiovascular safety assessments in the conscious telemetered dog: Utilisation of super-intervals to enhance statistical power." Journal of Pharmacological and Toxicological Methods 62(1): 12-19.	Title
442	Smith, C. and M. M. Koola (2016). "Evidence for using doxazosin in the treatment of posttraumatic stress disorder." Psychiatric annals 46(9): 553-555.	Title
443	Smith, D. A., R. S. Obach, D. P. Williams and B. K. Park (2009). "Clearing the MIST (metabolites in safety testing) of time: The impact of duration of administration on drug metabolite toxicity." Chemico-Biological Interactions 179(1): 60-67.	Title
444	Speakman, M. (2007). "Impact of Tamsulosin OCAS on Energy of Patients with LUTS/BPH." european urology supplements 6(9): 594-599.	Title
445	Sripalakit, P., P. Nermhom and A. Saraphanchotiwitthaya (2005). "Improvement of doxazosin determination in human plasma using high-performance liquid chromatography with fluorescence detection." Journal of chromatographic science 43(2): 63-66.	Full text
446	Steers, W. D. and R. S. Kirby (2005). "Clinical ease of using doxazosin in BPH patients with and without hypertension." Prostate Cancer Prostatic Dis 8(2): 152-157.	Title
447	Steiner, J. A. (1991). Antihypertensive drugs. Side Effects of Drugs Annual. M. N. G. Dukes and J. K. Aronson, Elsevier. 15: 200-211.	Title
448	Stokes, G. S. (1988). "Age-related effects of antihypertensive therapy with alpha-blockers." J Cardiovasc Pharmacol 12 Suppl 8: S109-115.	Review
449	Stokes, G. S., H. J. Johnston, E. O. Okoro, J. Boutagy, J. C. Monaghan and J. F. Marwood (1994). "Comparative and combined efficacy of doxazosin and enalapril in hypertensive patients." Clin Exp Hypertens 16(6): 709-727.	Abstract

Sr no.	Title	Reason
450	Stokes, G. S., J. C. Monaghan, J. F. Marwood, E. O. Okoro and H. Johnston (1992). "Increase in serum total angiotensin-converting enzyme activity with enalapril therapy in humans: a controlled trial." Clin Exp Pharmacol Physiol 19(5): 297-299.	Title
451	Stowasser, M., A. H. Ahmed, E. Pimenta, P. J. Taylor and R. D. Gordon (2012). "Factors affecting the aldosterone/renin ratio." Horm Metab Res 44(3): 170-176.	Title
452	Streeter, A. J. and E. C. Faria (2017). "Analysis of the variability of the pharmacokinetics of multiple drugs in young adult and elderly subjects and its implications for acceptable daily exposures and cleaning validation limits." International Journal of Hygiene and Environmental Health 220(4): 659-672.	Title
453	Striffler, J. S., S. J. Bhathena, O. E. Michaelis, J. D. Campbell, C. T. Hansen, E. Scalbert, N. Thibault and M. T. Velasquez (1998). "Long-term effects of perindopril on metabolic parameters and the heart in the spontaneously hypertensive/NIH-corpulent rat with non—insulin-dependent diabetes mellitus and hypertension." Metabolism 47(10): 1199-1204.	Title
454	Strom, J. A., B. Zola, W. Frishman, A. Laddu, J. P. Wexler, K. Carlson and A. Jordan (1991). "Acute hemodynamic effects of terazosin in hypertensive and normotensive patients." American Heart Journal 122(3, Part 2): 892-900.	Title
455	Studer, J. A. and R. W. Piepho (1993). "Antihypertensive therapy in the geriatric patient: II. A review of the alpha1-adrenergic blocking agents." J Clin Pharmacol 33(1): 2-13.	Review
456	Stumpe, K. O., B. Hofling, R. Kolloch, R. M. Lederle, G. Wambach, H. Zschiedrich and M. Warmbold (1995). "Selective α1-receptor blockade by doxazosin. Antihypertensive and metabolic effects in comparison to doxazosin." Munchener medizinische Wochenschrift (1950) 137(41): 650-655.	Title
457	Sun, J. A., D. Z. Kong, Y. Q. Zhen, Q. Li, W. Zhang, J. H. Zhang, Z. W. Yin and L. M. Ren (2013). "Stereoselective binding of doxazosin enantiomers to plasma proteins from rats, dogs and humans in vitro." Acta Pharmacol Sin 34(12): 1568-1574.	Full text
458	Suzuki, H., Y. Kanno, S. Sugahara, H. Okada and H. Nakamoto (2004). "Effects of an angiotensin II receptor blocker, valsartan, on residual renal function in patients on CAPD." American journal of kidney diseases 43(6): 1056-1064.	Title
459	Swindella, A. C., M. N. Krupp, T. M. Twomey, J. A. Reynolds and C. O. Chichester (1993). "Effects of doxazosin on atherosclerosis in cholesterol-fed rabbits." Atherosclerosis 99(2): 195-206.	Title
460	Tada, H. (2005). "Effect of candesartan cilexetil and imidapril hydrochloride on the urinary albumin and fibronectin levels of hypertensive type 2 diabetes patients with early nephropathy." Journal of the japan diabetes society 48(7): 481-486.	Title

Sr no.	Title	Reason
461	Takara, K., T. Sakaeda, M. Kakumoto, Y. Tanigawara, H. Kobayashi, K. Okumura, N. Ohnishi and T. Yokoyama (2009). "Effects of alpha-adrenoceptor antagonist doxazosin on MDR1-mediated multidrug resistance and transcellular transport." Oncol Res 17(11-12): 527-533.	Title
462	Takata, Y., T. Yoshizumi, Y. Ito, Y. Hirota and M. Fujishima (1994). "Doxazosin withdrawal and ambulatory blood pressure monitoring in normotensive subjects." Angiology 45(1): 17-23.	Title
463	Takata, Y., T. Yoshizumi, Y. Ito, Y. Hirota and M. Fujishima (1995). "Effect of administration and withdrawal of doxazosin on ambulatory blood pressure in patients with essential hypertension." Angiology 46(1): 11-18.	Title
464	Talseth, T., L. Westlie and L. N. Daae (1990). "Long-term effects of doxazosin and atenolol on serum lipids and blood pressure in hypertensive smokers." Journal of hypertension. Supplement 8(5): S47-51.	Title
465	Tamasawa, N., J. Matsui, Y. Ogawa, T. Gotoh, T. Hinata, H. Murakami, G. J. Zhi and T. Suda (2000). "Effect of doxazosin on the size of LDL particle in the type 2 diabetic patients with hypertension." Journal of diabetes and its complications 14(3): 135-139.	Title
466	Tanna, S., D. Bernieh and G. Lawson (2015). "LC-HRMS analysis of dried blood spot samples for assessing adherence to cardiovascular medications."	Title
467	Tarhan, F., O. Çelik, Ç. Tosun, G. Faydacı and B. Eryıldırım (2014). "Comparison of the efficacy of isosorbide mononitrate and doxazosin in the treatment of lower urinary tract symptoms and benign prostatic hyperplasia: a randomized clinical trial." Urologia internationalis 93(1): 17-21.	Title
468	Taylor, S. H. (1988). "Pharmacotherapeutic stature of doxazosin and its role in coronary risk reduction." American Heart Journal 116(6): 1735-1747.	Title
469	Taylor, S. H. (1989). "Clinical pharmacotherapeutics of doxazosin." The American Journal of Medicine 87(2): S2-S11.	Full text
470	Tctr (2019). "The combining studies of 3-sections for bioequivalence studies of randomized, open-label, two-way crossover design with two-period, two-treatment and two-sequence of Doxazosin Mesylate Modified Release Tablet 4.85 mg eq. to Doxazosin 4 mg relative to Originator Doxazosin XL Tablet (4 mg) in healthy Thai volunteers after single dose under fasting condition (Section-A), single dose under fed condition (Section-B) and multiple dose under fasting condition (Section-C)." https://trialsearch.who.int/Trial2.aspx?TrialID=TCTR20190215003.	Title
471	Te, A. E. (2002). "A modern rationale for the use of phenoxybenzamine in urinary tract disorders and other conditions." Clinical Therapeutics 24(6): 851-861.	Title

Sr no.	Title	Reason
472	Thomas, D., R. Bloehs, R. Koschny, E. Ficker, J. Sykora, J. Kiehn, K. Schlömer, J. Gierten, S. Kathöfer, E. Zitron, E. P. Scholz, C. Kiesecker, H. A. Katus and C. A. Karle (2008). "Doxazosin induces apoptosis of cells expressing hERG K+ channels." Eur J Pharmacol 579(1-3): 98-103.	Title
473	Thorstensen, C. W., P. E. Clasen, S. Rognstad, R. Haldsrud, S. Føreid, T. Helstrøm, O. U. Bergland, L. V. Halvorsen, A. Aune, E. Olsen, K. M. Brobak, A. Høieggen, I. Gustavsen, A. C. K. Larstorp, C. L. Søraas and M. S. Opdal (2022). "Development of UHPLC-MS/MS methods to quantify 25 antihypertensive drugs in serum in a cohort of patients treated for hypertension." J Pharm Biomed Anal 219: 114908.	Full text
474	Torvik, D. and H. P. Madsbu (1987). "An open one-year comparison of doxazosin and prazosin for mild to moderate essential hypertension." Am J Cardiol 59(14): 68g-72g.	Title
475	Trost, B. N., P. Weidmann, W. Riesen, J. Claessens, Y. Streulens and F. Nelemans (1987). "Comparative effects of doxazosin and hydrochlorothiazide on serum lipids and blood pressure in essential hypertension." The American Journal of Cardiology 59(14): G99-G104.	Title
476	Tubaro, A. and C. De Nunzio (2004). "Comparison of Peripherally Acting Substance for Treatment of Detrusor Overactivity: What is New; What is in the Pipeline?" EAU Update Series 2(4): 161-169.	Title
477	Tylicki, L., M. Renke, P. Rutkowski, W. Larczyński, E. Aleksandrowicz, W. Lysiak-Szydlowska and B. Rutkowski (2008). "Dual blockade of the renin-angiotensin-aldosterone system with high-dose angiotensin-converting enzyme inhibitor for nephroprotection: an open, controlled, randomized study." Scandinavian journal of urology and nephrology 42(4): 381-388.	Title
478	Uchida, S., H. Watanabe, S. Nishio, H. Hashimoto, K. Yamazaki, H. Hayashi and K. Ohashi (2003). "Altered pharmacokinetics and excessive hypotensive effect of candesartan in a patient with the CYP2C91/3 genotype." Clin Pharmacol Ther 74(5): 505-508.	Title
479	Ulahannan, T. J., F. Karpe, S. M. Humphreys, D. R. Matthews and K. N. Frayn (2002). "Effects of acute administration of doxazosin on fasting and postprandial haemodynamics and lipid metabolism in healthy subjects." Horm Metab Res 34(9): 499-503.	Abstract
480	Uzunlulu, M., A. Oguz and E. Yorulmaz (2006). "The effect of carvedilol on metabolic parameters in patients with metabolic syndrome." International heart journal 47(3): 421-430.	Title

Sr no.	Title	Reason
481	Valko, K., E. Chiarparin, S. Nunhuck and D. Montanari (2012). "In Vitro Measurement of Drug Efficiency Index to Aid Early Lead Optimization." Journal of Pharmaceutical Sciences 101(11): 4155-4169.	Title
482	van Brummelen, P., K. Jie, P. Vermey, P. B. Timmermans and P. A. van Zwieten (1985). "Vascular alpha-adrenoceptors in man: interactions with adrenaline and noradrenaline." Clinical science (London, England : 1979) 68 Suppl 10: 151s-153s.	Title
483	van Kerrebroeck, P., A. Jardin, P. van Cangh and K. U. Laval (2002). "Long-Term Safety and Efficacy of a Once-Daily Formulation of Alfuzosin 10 mg in Patients with Symptomatic Benign Prostatic Hyperplasia: Open-Label Extension Study." European Urology 41(1): 54-61.	Title
484	van Zwieten, P. A. (1992). "[Pharmacologic profile of urapidil. Consequences for use as an antihypertensive drug]." Fortschr Med 110(17): 330-332.	Title
485	Van Zwieten, P. A. and B. N. C. Prichard (1989). "Urapidil : an antihypertensive drug with multifactorial action." Annales Françaises d'Anesthésie et de Réanimation 8(5): 568-571.	Title
486	Van Zwieten, P. A., P. B. M. W. M. Timmermans and P. Van Brummelen (1984). "Role of Alpha Adrenoceptors in Hypertension and in Anti hypertensive Drug Treatment." The American Journal of Medicine 77(4, Part 1): 17-25.	Title
487	Varani, K., R. Manfredini, V. Iannotta, C. Pancaldi, E. Cattabriga, C. Uluoglu, P. A. Borea and F. Portaluppi (2002). "Effects of doxazosin and propranolol on A2A adenosine receptors in essential hypertension." Hypertension 40(6): 909-913.	Title
488	Vashi, V., M. Chung, J. Hilbert, V. Lawrence and K. Phillips (1998). "Pharmacokinetic interaction between finasteride and terazosin, but not finasteride and doxazosin." The Journal of Clinical Pharmacology 38(11): 1072-1076.	Full text
489	Veglio, F., D. Schiavone, A. Rossi and L. Chiandussi (1994). "Acute effects of alpha-1 adrenoceptor antagonist, doxazosin on circulating vasoactive hormones." Riv Eur Sci Med Farmacol 16(3-4): 85-90.	Title
490	Veldhuizen, G. P., R. M. Alnazer, P. W. de Leeuw and A. A. Kroon (2023). "The Effects of Verapamil, Hydralazine, and Doxazosin on Renin, Aldosterone, and the Ratio Thereof." Cardiovasc Drugs Ther 37(2): 283-289.	Title
491	Velussi, M., A. M. Cernigoi, L. Viezzoli and C. Caffau (1993). "Treatment of arterial hypertension in non-insulin-dependent diabetic patients: comparison of doxazosin and enalapril." Current therapeutic research - clinical and experimental 53(3): 316-328.	Title
492	Verplaetse, T. L., A. H. Weinberger, L. M. Oberleitner, K. M. Smith, B. P. Pittman, J. M. Shi, J. M. Tetrault, M. E. Lavery, M. R. Picciotto and S. A. McKee (2017). "Effect	Title

Sr no.	Title	Reason
	of doxazosin on stress reactivity and the ability to resist smoking." Journal of psychopharmacology (Oxford, England) 31(7): 830-840.	
493	Versmissen, J., B. C. Koch, D. W. Roofthooft, W. Ten Bosch-Dijksman, A. H. van den Meiracker, L. M. Hanff and W. Visser (2016). "Doxazosin treatment of phaeochromocytoma during pregnancy: placental transfer and disposition in breast milk." Br J Clin Pharmacol 82(2): 568-569.	Full text
494	Vet—QC01CA14, A. (2005). "1274 Cardiovascular Drugs." Intern Med 142: 510-524.	Title
495	Vet—QC02CA04, A. (1987). "Doxazosin Mesilate (BANM, rINNM)." Cardiol 59: 78G-81G.	Title
496	Vincent, J. (1985). Clinical and Experimental Studies With alpha1 Adrenoceptor Antagonists, University of Glasgow (United Kingdom).	Title
497	Vincent, J., H. L. Elliott, P. A. Meredith and J. L. Reid (1983). "Clinical pharmacological studies with doxazosin: pharmacokinetics and concentration-effect relationships." British journal of clinical pharmacology 15(1): 136P.	Full text
498	Vincent, J., H. L. Elliott, P. A. Meredith and J. L. Reid (1985). "The effect of age on the pharmacokinetics and pharmacodynamics of doxazosin." British journal of clinical pharmacology 20: 251P-252P.	No Access
499	Vincent, J., P. Meredith, H. Elliott and J. Reid (1986). "The pharmacokinetics of doxazosin in elderly normotensives." British journal of clinical pharmacology 21(5): 521-524.	Full text
500	Viswanathan, H., M. Bharmal and J. Thomas (2005). "Prevalence and correlates of potentially inappropriate prescribing among ambulatory older patients in the year 2001: Comparison of three explicit criteria." Clinical Therapeutics 27(1): 88-99.	Title
501	Wadworth, A. N., D. Murdoch and R. N. Brogden (1991). "Atenolol. A reappraisal of its pharmacological properties and therapeutic use in cardiovascular disorders." Drugs 42(3): 468-510.	Title
502	Wajima, T., K. Fukumura, Y. Yano and T. Oguma (2003). "Prediction of Human Pharmacokinetics from Animal Data and Molecular Structural Parameters using Multivariate Regression Analysis: Oral Clearance." Journal of Pharmaceutical Sciences 92(12): 2427-2440.	Title
503	Waller, D. and R. Oliver (1990). "Doxazosin in renal impairment." Journal of Human Hypertension 4: 47-49.	No Access
504	Wang, C. C., L. Sombra and L. Fernández (2012). "Automated flow system for sildenafil enrichment using surfactant coated solid-phase with fluorescence detection." Talanta 98: 247-252.	Title

Sr no.	Title	Reason
505	Warrington, F. S. S. S. H. and W. Lancashire "DOXAZOSIN modified release (M/R) tablets (All Brands)."	Title
506	Watanabe, H., M. Kakihana, S. Ohtsuka and Y. Sugishita (1998). "Randomized, double-blind, placebo-controlled study of carvedilol on the prevention of nitrate tolerance in patients with chronic heart failure." Journal of the American College of Cardiology 32(5): 1194-1200.	Title
507	Watson, W. A., T. L. Litovitz, G. C. Rodgers, W. Klein-schwartz, J. Youniss, S. R. Rose, D. Borys and M. E. May (2003). "2002 annual report of the American association of poison control centers toxic exposure surveillance system 1 1US poison centers make possible the compilation and reporting of this comprehensive description of human exposures to potentially toxic substances through their meticulous documentation of each case using standardized definitions and compatible computer systems. Centers participating in this report include Regional Poison Control Center, Birmingham, AL Alabama Poison Center, Tuscaloosa, AL; Arizona Poison and Drug Information Center, Tucson, AZ; Banner Poison Control Center, Phoenix, AZ; Arkansas Poison and Drug Information Center, Little Rock, AR; California Poison Control System—Fresno/Madera Division, CA; California Poison Control System—Sacramento Division, CA; California Poison Control System—San Diego Division, CA; California Poison Control System—San Francisco Division, CA; Rocky Mountain Poison and Drug Center, Denver, CO; Connecticut Poison Control Center, Farmington, CT; National Capital Poison Center, Washington, DC; Florida Poison Information Center, Tampa, FL; Florida Poison Information Center, Jacksonville, FL; Florida Poison Information Center, Miami, FL; Georgia Poison Center, Atlanta, GA; Illinois Poison Center, Chicago, IL; Indiana Poison Center, Indianapolis, IN; Iowa Statewide Poison Control Center, Sioux City, IA; Mid-America Poison Control Center, Kansas City, KS; Kentucky Regional Poison Center, Louisville, KY; Louisiana Drug and Poison Information Center, Monroe, LA; Northern New England Poison Center, Portland, ME; Maryland Poison Center, Baltimore, MD; Regional Center for Poison Control and Prevention Serving Massachusetts and Rhode Island, Boston, MA; Children’s Hospital of Michigan Regional Poison Control Center, Detroit, MI; DeVos Children’s Hospital Regional Poison Center, Grand Rapids, MI; Hennepin Regional Poison Center, Minneapolis, MN; Mississippi Regional Poison Control Center, Jackson, MS; Missouri Regional Poison Center, St. Louis, MO; The Poison Center, Omaha, NE; New Hampshire Poison Information Center, Lebanon, NH; New Jersey Poison Information and Education System, Newark, NJ; New Mexico Poison and Drug Information Center, Albuquerque, NM; New York City Poison Control Center, New York, NY; Long Island Regional	Title

Sr no.	Title	Reason
	Poison and Drug Information Center, Mineola, NY; Finger Lakes Regional Poison and Drug Information Center, Rochester, NY; Central New York Poison Center, Syracuse, NY; Western New York Poison Center, Buffalo, NY; Carolinas Poison Center, Charlotte, NC; Cincinnati Drug and Poison Information Center, Cincinnati, OH; Central Ohio Poison Center, Columbus, OH; Greater Cleveland Poison Control Center, Cleveland, OH; Oklahoma Poison Control Center, Oklahoma City, OK; Oregon Poison Center, Portland, OR; Pittsburgh Poison Center, Pittsburgh, PA; The Poison Control Center, Philadelphia, PA; Penn State Poison Center, Hershey, PA; San Jorge Children's Hospital Poison Center, Santurce, PR; Palmetto Poison Center, Columbia, SC; Middle Tennessee Poison Center, Nashville, TN; Southern Poison Center, Memphis, TN; Central Texas Poison Center, Temple, TX; North Texas Poison Center, Dallas, TX; Southeast Texas Poison Center, Galveston, TX; Texas Panhandle Poison Center, Amarillo, TX; West Texas Regional Poison Center, El Paso, TX; South Texas Poison Center, San Antonio, TX; Utah Poison Control Center, Salt Lake City, UT; Virginia Poison Center, Richmond, VA; Blue Ridge Poison Center, Charlottesville, VA; Washington Poison Center, Seattle, WA; West Virginia Poison Center, Charleston, WV; and Children's Hospital of Wisconsin Poison Center, Milwaukee, WI.© 1985–2003 by the American Association of Poison Control Centers. Published by permission. All rights reserved.Reprints are available at a cost of $10 each. Address requests to AAPCC, 3201 New Mexico Ave, Suite 330, Washington, DC 20016." The American Journal of Emergency Medicine 21(5): 353-421.	
508	Waybill, M. M. and P. N. Waybill (2003). "A Practical Approach to Hypertension in the 21st Century." Journal of Vascular and Interventional Radiology 14(8): 961-975.	Title
509	Weber, M. A., W. F. Graettinger and J. I. M. Drayer (1987). "The Adrenergic Inhibitors." Medical Clinics of North America 71(5): 959-977.	Title
510	Wehmeyer, A., R. Coetzee, N. Hoffman, Y. Johnson and R. Kloppers (2020). "Medicine-use evaluation of doxazosin prescribing to inform formulary recommendations." South African Medical Journal 110(1): 16-20.	Title
511	Wei, X., J. Yin, G. Yang, C. He and Y. Chen (2007). "On-line solid-phase extraction with a monolithic weak cation-exchange column and simultaneous screening of alpha1-adrenergic receptor antagonists in human plasma." J Sep Sci 30(17): 2851-2857.	Title
512	Weinberger, M. and A. Fawzy (2000). "Doxazosin in elderly patients with hypertension." International journal of clinical practice 54(3): 181-189.	No Access
513	Weir, M. R., J. M. Flack and W. B. Applegate (1996). "Tolerability, safety, and quality of life and hypertensive therapy: The case for low-dose diuretics." The American Journal of Medicine 101(3, Supplement 1): 83S-92S.	Title

Sr no.	Title	Reason
514	Welk, B. and J. A. McClure (2023). "THE REDUCTION OF MALE URINARY SYMPTOMS IS ASSOCIATED WITH DECREASED MORTALITY." Neurourology and urodynamics 42: S277.	Title
515	Wells, T. and C. Stowe (2001). "An approach to the use of antihypertensive drugs in children and adolescents." Current Therapeutic Research 62(4): 329-350.	Title
516	Whalley, D. G. and M. J. Berrigan (2000). "ANESTHESIA FOR RADICAL PROSTATECTOMY, CYSTECTOMY, NEPHRECTOMY, PHEOCHROMOCYTOMA, AND LAPAROSCOPIC PROCEDURES." Anesthesiology Clinics of North America 18(4): 899-917.	Title
517	Whitworth, J. A., J. Butty and D. Gordon (1987). "Acute haemodynamic and hormonal effects of oral doxazosin in normal subjects." Clin Exp Pharmacol Physiol 14(2): 133-135.	Title
518	Williams, B., T. M. MacDonald, S. Morant, D. J. Webb, P. Sever, G. McInnes, I. Ford, J. K. Cruickshank, M. J. Caulfield, J. Salsbury and et al. (2015). "Spironolactone versus placebo, bisoprolol, and doxazosin to determine the optimal treatment for drug-resistant hypertension (PATHWAY-2): a randomised, double-blind, crossover trial." Lancet (london, england) 386(10008): 2059-2068.	Title
519	Williams, B., T. M. MacDonald, S. V. Morant, D. J. Webb, P. Sever, G. T. McInnes, I. Ford, J. K. Cruickshank, M. J. Caulfield, S. Padmanabhan, I. S. Mackenzie, J. Salsbury and M. J. Brown (2018). "Endocrine and haemodynamic changes in resistant hypertension, and blood pressure responses to spironolactone or amiloride: the PATHWAY-2 mechanisms substudies." Lancet Diabetes Endocrinol 6(6): 464-475.	Title
520	Wilner, K. D. and M. G. Ziegler (1987). "Effects of alphasub 1 inhibition on renal blood flow and sympathetic nervous activity in systemic hypertension." American journal of cardiology 59(14): 82G-86G.	Title
521	Wilson, I. D. (2004). Chapter 20 Drugs. Journal of Chromatography Library. E. Heftmann, Elsevier. 69: 945-985.	Title
522	Witte, D. G., M. E. Brune, S. P. Katwala, I. Milicic, D. Stolarik, Y. H. Hui, K. C. Marsh, J. F. Kerwin, Jr., M. D. Meyer and A. A. Hancock (2002). "Modeling of relationships between pharmacokinetics and blockade of agonist-induced elevation of intraurethral pressure and mean arterial pressure in conscious dogs treated with alpha(1)-adrenoceptor antagonists." J Pharmacol Exp Ther 300(2): 495-504.	Title
523	Witte, K. K., S. D. Thackray, N. P. Nikitin, J. G. Cleland and A. L. Clark (2003). "The effects of alpha and beta blockade on ventilatory responses to exercise in chronic heart failure." Heart (British Cardiac Society) 89(10): 1169-1173.	Title

Sr no.	Title	Reason
524	Wong, C., D. H. Muguiro, S. Lavergne, E. Behling-Kelly and R. Goggs (2018). "Pharmacokinetics of human recombinant C1-esterase inhibitor and development of anti-drug antibodies in healthy dogs." Veterinary Immunology and Immunopathology 203: 66-72.	Title
525	Wong, J., R. A. Patel and P. R. Kowey (2004). "The clinical use of angiotensin-converting enzyme inhibitors." Progress in Cardiovascular Diseases 47(2): 116-130.	Title
526	Wonnemann, M., B. Schug, K. Schmücker, E. Brendel, P. Van Zwieten and H. Biume (2006). "Significant food interactions observed with a nifedipine modified-release formulation marketed in the European Union." International Journal of Clinical Pharmacology & Therapeutics 44(1).	Title
527	Wright, J. T., S. Harris-Haywood, S. Pressel, J. Barzilay, C. Baimbridge, C. J. Bareis, J. N. Basile, H. R. Black, R. Dart, A. K. Gupta and et al. (2008). "Clinical outcomes by race in hypertensive patients with and without the metabolic syndrome: antihypertensive and Lipid-Lowering Treatment to Prevent Heart Attack Trial (ALLHAT)." Archives of internal medicine 168(2): 207-217.	Title
528	Wright, J. T., J. L. Probstfield, W. C. Cushman, S. L. Pressel, J. A. Cutler, B. R. Davis, P. T. Einhorn, M. Rahman, P. K. Whelton, C. E. Ford and et al. (2009). "ALLHAT findings revisited in the context of subsequent analyses, other trials, and meta-analyses." Archives of internal medicine 169(9): 832-842.	Title
529	Wu, C.-P., S.-H. Hsiao and Y.-S. Wu (2023). "Perspectives on drug repurposing to overcome cancer multidrug resistance mediated by ABCB1 and ABCG2." Drug Resistance Updates 71: 101011.	Title
530	Wu, J.-T. and D. A.Wells (2003). Chapter 14 On-line sample preparation: High throughput techniques and strategies for method development. Progress in Pharmaceutical and Biomedical Analysis. D. A. Wells, Elsevier. 5: 505-573.	Title
531	Wykretowicz, A., P. Guzik and H. Wysocki (2008). "Doxazosin in the current treatment of hypertension." Expert opinion on pharmacotherapy 9(4): 625-633.	Review
532	Wyllie, M. G. (1999). "alpha1-adrenoceptor selectivity: the North American experience." Eur Urol 36 Suppl 1: 59-63; discussion 65.	Title
533	Yamasaki, Y., Y. Shiba, M. Sekiya, T. Tsujino, N. Hakui, R. Kawamori and T. Kamada (1994). "Selective alpha 1-adrenergic inhibition improves decrease glucose disposal in patients with essential hypertension." J Hum Hypertens 8(8): 555-558.	Title
534	Yamoah, G. E. and O. L'E (1986). "Typeset by Oxprint Ltd, Oxford Printed in Great Britain." British Journal of Clinical Pharmacology 21(6).	Title
535	Yang, G., H. Liu, Y. Zhang, S. Wang, J. Yin, B. Yin and Y. Chen (2006). "On-line simultaneous removal of human serum albumin and enrichment of doxazosin using a	Title

Sr no.	Title	Reason
	weak cation-exchange monolithic column." Journal of Chromatography A 1129(2): 231-235.	
536	Yang, S. C., W. I. Tsai, C. S. Liau and T. D. Wang (2013). "Effect of 8-week combination therapy with an extended-release α1-blocker (bunazosin or doxazosin) in inadequate responders to an angiotensin ii antagonist (valsartan) in patients with stage 1 or 2 essential hypertension." Acta Cardiologica Sinica 29(1): 11-18.	Title
537	Yasin, Ö., Y. KANAL and Ç. YAYLA (2022). Güncel Kardiyak İlaçlar, Akademisyen Kitabevi.	Language
538	Yasunari, K., T. Matsui, K. Maeda, M. Nakamura, T. Watanabe and N. Kiriike (2006). "Anxiety-induced plasma norepinephrine augmentation increases reactive oxygen species formation by monocytes in essential hypertension." American journal of hypertension 19(6): 573-578.	Title
539	Yildiz, A., M. Hursit, A. V. Celik, S. M. Kayacan, H. Yazici, V. Akkaya, A. O. Gürol and K. Karsidag (2002). "Doxazosin, but not amlodipine decreases insulin resistance in patients with chronic renal failure: a prospective, randomized-controlled study." Clinical nephrology 58(6): 405-410.	Title
540	Yilmaz, M. I., A. Sonmez, K. Caglar, T. Celik, M. Yenicesu, T. Eyileten, C. Acikel, Y. Oguz, I. Yavuz and A. Vural (2007). "Effect of antihypertensive agents on plasma adiponectin levels in hypertensive patients with metabolic syndrome." Nephrology (Carlton) 12(2): 147-153.	Title
541	Yono, M., H. E. Foster, D. Shin, W. Takahashi, M. Pouresmail and J. Latifpour (2004). "Doxazosin treatment causes differential alterations of α1-adrenoceptor subtypes in the rat kidney, heart and aorta." Life Sciences 75(21): 2605-2614.	Title
542	Yono, M., J. Foster, Harris E, D. Shin, W. Takahashi, M. Pouresmail and J. Latifpour (2004). "Doxazosin-induced up-regulation of α1A-adrenoceptor mRNA in the rat lower urinary tract." Canadian journal of physiology and pharmacology 82(10): 872-878.	Title
543	Young, R. A. and R. N. Brogden (1988). "Doxazosin: a review of its pharmacodynamic and pharmacokinetic properties, and therapeutic efficacy in mild or moderate hypertension." Drugs 35: 525-541.	Review
544	Zannad, F. (1999). Antihypertensive drugs. Side Effects of Drugs Annual. J. K. Aronson and J. Elis, Elsevier. 22: 224-232.	Title
545	Zehetgruber, M., G. Christ, H. Gabriel, G. Mundigler, R. Beckmann, B. R. Binder and K. Huber (1998). "Effect of antihypertensive treatment with doxazosin on insulin sensitivity and fibrinolytic parameters." Thromb Haemost 79(2): 378-382.	Title

Sr no.	Title	Reason
546	Zehri, A. A., M. H. Ather, F. Abbas and S. R. Biyabani (2010). "Preliminary study of efficacy of doxazosin as a medical expulsive therapy of distal ureteric stones in a randomized clinical trial." Urology 75(6): 1285-1288.	Title
547	Zhang, Y., Y. Wang, P. Zhang, X. D. Zhang and Y. Yang (2009). "Extended-Release Doxazosin for Treatment of Renal Transplant Recipients With Benign Prostatic Hyperplasia." Transplantation Proceedings 41(9): 3747-3751.	Abstract
548	Zhao, Y., X.-B. Cao and L.-M. Ren (2014). "Doxazosin selectively potentiates contraction to serotonin via 5-HT2A receptors in longitudinal muscle strips of the rabbit gastric body." Canadian Journal of Physiology and Pharmacology 92(3): 197-204.	Title
549	Zhao, Y. H., M. H. Abraham, A. Hersey and C. N. Luscombe (2003). "Quantitative relationship between rat intestinal absorption and Abraham descriptors." European Journal of Medicinal Chemistry 38(11): 939-947.	Title
550	Zhao, Y. H., M. H. Abraham, J. Le, A. Hersey, C. N. Luscombe, G. Beck, B. Sherborne and I. Cooper (2003). "Evaluation of rat intestinal absorption data and correlation with human intestinal absorption." European Journal of Medicinal Chemistry 38(3): 233-243.	Title
551	Zhen, Y.-Q., D.-Z. Kong, Q. Li, J. Zhao and L.-M. Ren (2013). "Determination of doxazosin enantiomers in rat plasma and investigation of their chiral inversion." Yao xue xue bao= Acta Pharmaceutica Sinica 48(6): 901-905.	Animal based
552	Леонова, М. (2009). "Пероральные лекарственные формы с модифицированным высвобождением кардиоваскулярных препаратов разных классов (тиазидов, триметазидина, β-блокаторов, гиполипидемических препаратов)." Сердце: журнал для практикующих врачей 8(4): 209-213.	Language

Supplementary Table S2. Quality Assessment of Included Articles based on JADAD Scoring

Sr No.	Was the study described as randomized	Was the method used to generate the sequence of randomization described and appropriate	Was the study described as double blind	Was the method of double blinding described and appropriate	Was there a description of withdrawals and dropouts	JADAD score	References
1	0	0	0	0	0	0	[42]
2	0	0	0	0	1	1	[40]
3	0	0	0	0	0	0	[46]
4	0	0	1	1	1	3	[41]
5	1	1	0	0	1	3	[44]
6	0	0	0	0	0	0	[33]
7	0	0	0	0	0	0	[38]
8	1	1	0	0	0	2	[30]
9	0	0	0	0	0	0	[29]
10	0	0	0	0	0	0	[36]
11	1	1	1	0	0	3	[27]
12	1	1	1	1	1	5	[34]
13	1	1	0	0	0	2	[63]
14	1	1	0	0	1	3	[39]
15	0	0	0	0	0	0	[43]
16	1	1	0	0	0	2	[31]
17	1	1	0	0	1	3	[35]
18	1	1	0	0	0	2	[13]
19	0	0	0	0	0	0	[45]
20	1	1	0	0	1	3	[28]
21	0	0	0	0	1	1	[47]
22	0	0	0	0	0	0	[37]
23	1	1	0	0	0	2	[51]
24	1	1	1	1	1	5	[49]
25	1	0	0	0	0	1	[50]

0= No, **1**= Yes.

Supplementary Table S3. Quality Assessment of Included Articles based on Critical Appraisal Skills Program (CASP) Scoring

**Sr no.**	**Q1**	**Q2**	**Q3**	**Q4**	**Q5**	**Q6**	**Q7**	**Q8**	**Q9**	**Q10**	**Score out of 10**	**References**
1	Y	Y	Y	Y	Y	Y	CT	CT	Y	Y	8	[42]
2	Y	Y	Y	Y	Y	Y	N	Y	Y	Y	9	[40]
3	Y	Y	Y	Y	Y	Y	CT	Y	Y	Y	9	[46]
4	Y	Y	Y	Y	Y	Y	CT	Y	Y	Y	9	[41]
5	Y	Y	Y	Y	Y	Y	CT	Y	Y	Y	9	[44]
6	Y	Y	Y	Y	Y	Y	CT	Y	Y	Y	9	[33]
7	Y	Y	Y	Y	CT	Y	N	N	Y	Y	7	[38]
8	Y	Y	Y	Y	CT	Y	N	Y	Y	CT	7	[30]
9	Y	Y	Y	Y	CT	Y	CT	Y	Y	Y	8	[29]
10	Y	Y	CT	Y	CT	Y	CT	Y	Y	Y	7	[36]
11	Y	Y	Y	Y	Y	Y	CT	Y	Y	Y	9	[27]
12	Y	Y	Y	Y	Y	Y	CT	Y	Y	Y	9	[43]
13	Y	Y	Y	Y	Y	Y	N	Y	Y	Y	9	[31]
14	Y	Y	Y	Y	Y	Y	N	Y	Y	Y	9	[34]
15	Y	Y	Y	Y	Y	Y	N	Y	Y	Y	9	[39]
16	Y	Y	Y	Y	Y	Y	N	CT	Y	CT	7	[63]
17	Y	Y	Y	Y	Y	Y	N	Y	Y	Y	9	[35]
18	Y	Y	Y	Y	Y	Y	N	CT	Y	Y	8	[13]
19	Y	Y	Y	Y	Y	Y	N	Y	Y	Y	9	[45]
20	Y	Y	Y	Y	Y	Y	N	CT	Y	Y	8	[28]
21	Y	Y	Y	Y	Y	Y	N	Y	Y	Y	9	[47]
22	Y	Y	Y	Y	Y	Y	N	CT	Y	Y	8	[37]
23	Y	Y	Y	Y	Y	Y	N	Y	Y	Y	9	[50]
24	Y	Y	Y	Y	Y	Y	N	Y	Y	Y	9	[51]
25	Y	Y	Y	Y	Y	Y	N	Y	Y	Y	9	[49]

Y= YES, N= NO, CT= CAN'T TELL

Questions:

Q 1: Was there a clear statement of the aims of the research?

Q 2: Is a qualitative methodology appropriate?

Q 3: Was the research design appropriate to address the aims of the research?

Q 4: Are the study's theoretical underpinnings clear, consistent, and conceptually coherent?

Q 5: Was the recruitment strategy appropriate to the aims of the search?

Q 6: Was the data collected in a way that addressed the research issue?

Q 7: Has the relationship between researchers and participants been adequately considered?

Q 8: Have ethical issues been taken into consideration?

Q 9: Was the data analysis sufficiently rigorous?

Q 10: Is there a clear statement of findings?

Supplementary Table S4. Quality Assessment of Included Articles based on Critical Appraisal Clinical Pharmacokinetics Tool (CACPK) Scoring

Sr no.	**Q1**	**Q2**	**Q3**	**Q4**	**Q5**	**Q6**	**Q7**	**Q8**	**Q9**	**Q10**	**Q11**	**Q12**	**Q13**	**Q14**	**Q15**	**Q16**	**Q17**	**Q18**	**Q19**	**Q20**	**Q21**	**Total Score**	**References**
1	Y	Y	Y	Y	Y	N	Y	Y	Y	Y	Y	IDK	N	Y	Y	Y	N	Y	Y	IDK	Y	16	[42]
2	Y	Y	Y	Y	Y	Y	IDK	Y	Y	Y	Y	Y	Y	N	Y	Y	Y	Y	N	Y	Y	18	[40]
3	Y	Y	Y	Y	Y	Y	IDK	Y	Y	Y	Y	IDK	Y	IDK	Y	Y	N	Y	Y	IDK	Y	16	[46]
4	Y	Y	Y	Y	Y	Y	Y	IDK	Y	Y	Y	IDK	Y	N	IDK	Y	N	Y	N	Y	Y	15	[41]
5	Y	Y	Y	Y	Y	Y	Y	Y	Y	Y	Y	Y	Y	N	Y	Y	N	Y	N	Y	Y	18	[44]

Sr no.	**Q1**	**Q2**	**Q3**	**Q4**	**Q5**	**Q6**	**Q7**	**Q8**	**Q9**	**Q10**	**Q11**	**Q12**	**Q13**	**Q14**	**Q15**	**Q16**	**Q17**	**Q18**	**Q19**	**Q20**	**Q21**	**Total Score**	**References**
6	Y	Y	Y	Y	Y	N	N	Y	Y	Y	Y	IDK	Y	N	IDK	Y	Y	Y	Y	Y	Y	16	[33]
7	Y	Y	Y	Y	Y	N	N	Y	Y	Y	Y	IDK	Y	N	Y	Y	N	IDK	Y	IDK	Y	14	[38]
8	Y	Y	Y	Y	Y	N	N	IDK	Y	Y	Y	Y	N	N	Y	Y	N	Y	Y	IDK	Y	14	[30]
9	Y	Y	Y	Y	Y	N	N	Y	Y	Y	Y	Y	Y	IDK	Y	Y	N	Y	Y	Y	Y	17	[29]
10	Y	Y	Y	Y	Y	N	N	IDK	Y	Y	Y	IDK	N	N	IDK	IDK	Y	N	Y	IDK	IDK	10	[36]
11	Y	Y	Y	Y	Y	N	IDK	Y	Y	Y	Y	IDK	Y	IDK	Y	Y	Y	Y	Y	Y	Y	17	[27]
12	Y	Y	Y	Y	Y	Y	Y	Y	Y	Y	Y	Y	Y	Y	Y	Y	N	Y	N	Y	Y	19	[43]
13	Y	Y	Y	Y	Y	N	Y	Y	Y	Y	Y	IDK	Y	Y	Y	Y	N	Y	Y	IDK	Y	17	[31]
14	Y	Y	Y	Y	Y	Y	Y	Y	Y	Y	Y	IDK	Y	IDK	Y	Y	N	Y	N	Y	Y	17	[34]

Sr no.	**Q1**	**Q2**	**Q3**	**Q4**	**Q5**	**Q6**	**Q7**	**Q8**	**Q9**	**Q10**	**Q11**	**Q12**	**Q13**	**Q14**	**Q15**	**Q16**	**Q17**	**Q18**	**Q19**	**Q20**	**Q21**	**Total Score**	**References**
15	Y	Y	Y	Y	Y	Y	Y	Y	Y	Y	Y	IDK	Y	IDK	Y	Y	N	Y	Y	IDK	Y	17	[39]
16	Y	Y	Y	Y	Y	N	Y	IDK	Y	Y	Y	Y	Y	N	Y	Y	N	IDK	Y	IDK	Y	15	[63]
17	Y	Y	Y	Y	Y	Y	Y	Y	Y	Y	Y	N	Y	Y	Y	Y	Y	Y	Y	Y	Y	20	[35]
18	Y	Y	Y	Y	Y	N	IDK	N	Y	Y	Y	IDK	Y	N	IDK	Y	Y	Y	N	Y	Y	14	[13]
19	Y	Y	Y	Y	Y	N	Y	IDK	Y	Y	Y	N	N	Y	Y	Y	N	Y	Y	IDK	Y	15	[45]
20	Y	Y	Y	Y	Y	Y	Y	Y	Y	Y	Y	N	Y	N	Y	Y	N	Y	Y	IDK	Y	17	[28]
21	Y	Y	Y	Y	Y	Y	Y	IDK	Y	Y	Y	IDK	Y	N	Y	Y	N	Y	Y	IDK	Y	16	[47]
22	Y	Y	Y	Y	Y	N	N	N	Y	Y	Y	IDK	Y	Y	Y	Y	Y	N	Y	IDK	N	14	[37]
23	Y	Y	Y	Y	Y	N	Y	N	Y	N	N	N	N	N	IDK	Y	N	Y	Y	IDK	Y	11	[50]

Sr no.	Q1	Q2	Q3	Q4	Q5	Q6	Q7	Q8	Q9	Q10	Q11	Q12	Q13	Q14	Q15	Q16	Q17	Q18	Q19	Q20	Q21	Total Score	References
24	Y	Y	Y	Y	Y	N	N	N	Y	Y	Y	IDK	N	N	Y	Y	N	Y	Y	IDK	Y	13	[51]
25	Y	Y	Y	Y	Y	Y	IDK	N	Y	Y	Y	IDK	N	N	Y	Y	N	Y	Y	IDK	Y	14	[49]

Y= YES, N= NO, IDK= I DON'T KNOW

Questions:

Q 1: Was a clear description of the objectives of the study provided?

Q 2: Was a clear and comprehensive rationale provided to support the purpose of the study?

Q 3: Was the chosen study design appropriately selected and justified?

Q 4: Was the dosing (i.e. dose, route of administration, and dosing interval) of the drug in the study justified for the intended study?

Q 5: Were the outcome measures endpoints of the study appropriate to address the objectives of the study?

Q 6: Were the exclusion criteria of participants included AND appropriate for the intended outcomes of the study?

Q 7: Where applicable, were the relevant baseline characteristics of the participants adequately described?

Q 8: Were plausible interacting covariates described *a priori* or in post hoc evaluation?

Q 9: Was the description of the used biological sample analytical methods sample analysis methods or citations of prior validation studies provided in the publication or affiliated appendix?

Q 10: Was the method of data sampling of analytics appropriate for the study?

Q 11: Was a clear description of the sampling site provided and justified?

Q 12: Was the number of half-lives elapsed within the sampling period appropriate for the analyzed drug?

Q 13: Were sample storage conditions appropriate and described in a manner that could be accurately replicated?

Q 14: If applicable, was there a clear description of the pharmacokinetic model, its development, validation and justification for use?

Q 15: Was the described population pharmacokinetic approach validation method appropriate for the analysis?

Q 16: Were the essential pharmacokinetic parameters required to make the results applicable in clinical settings included?

Q 17: Were the pharmacokinetic equations used to calculate the patient's pharmacokinetic parameters presented or cited within the article?

Q 18: Were the chosen statistical tests and software to perform the statistical analysis appropriate to achieve the study objectives?

Q 19: Were all patients enrolled in the study accounted for?

Q 20: In the event of missing data or outliers, was the process for analysis justified and appropriate?

Q 21: Were appropriate summary statistics to describe centrality and variance used to present the pharmacokinetic results?

Supplementary Table S5. Assessment of risk of bias based on Cochrane Collaboration Tool (CCT)

Sr no.	**Random sequence Generation (selection bias)**	**Allocaton concealment (selection bias)**	**Blinding of participant and researchers (performed bias)**	**Blinding of outcome assessment (detection bias)**	**Incomplete outcome data (attrition bias)**	**Selective reporting (reporting bias)**	**Other bias**	**Total score**	**References**
1	UR	LR	UR	UR	LR	LR	LR	4	[42]
2	HR	HR	HR	HR	LR	LR	LR	3	[40]
3	HR	HR	HR	HR	LR	LR	LR	3	[46]
4	HR	LR	LR	LR	UR	LR	LR	5	[41]
5	LR	UR	UR	UR	LR	LR	LR	4	[44]
6	UR	HR	UR	UR	UR	LR	LR	2	[33]
7	UR	HR	HR	HR	UR	LR	LR	2	[38]
8	LR	UR	HR	HR	LR	LR	LR	4	[30]
9	UR	UR	HR	HR	LR	LR	LR	3	[29]
10	HR	HR	HR	HR	UR	LR	LR	2	[36]
11	LR	LR	LR	LR	UR	LR	LR	6	[27]
12	LR	LR	LR	LR	LR	LR	LR	6	[43]
13	LR	LR	UR	UR	LR	LR	UR	4	[31]
14	LR	LR	UR	UR	LR	LR	LR	5	[34]
15	HR	HR	UR	UR	LR	LR	UR	2	[39]
16	LR	LR	HR	HR	LR	LR	LR	5	[63]
17	LR	LR	HR	HR	LR	LR	LR	5	[35]

Sr no.	Random sequence Generation (selection bias)	Allocaton concealment (selection bias)	Blinding of participant and researchers (performed bias)	Blinding of outcome assessment (detection bias)	Incomplete outcome data (attrition bias)	Selective reporting (reporting bias)	Other bias	Total score	References
18	LR	LR	HR	HR	LR	LR	LR	5	[13]
19	HR	HR	UR	UR	LR	LR	UR	2	[45]
20	LR	LR	HR	HR	LR	LR	LR	5	[28]
21	HR	HR	HR	HR	LR	LR	UR	2	[47]
22	HR	HR	HR	HR	LR	LR	UR	2	[37]
23	LR	LR	UR	HR	LR	LR	UR	4	[51]
24	LR	LR	LR	LR	LR	LR	LR	7	[49]
25	LR	LR	UR	HR	LR	LR	HR	4	[50]

HR= HIGH RISK, LR= LOW RISK, UR= UNKNOWN RISK

Supplementary Table S6. Prisma Checklist 2020.

Section and Topic	Item #	Checklist item	Location where item is reported
**TITLE**			
Title	1	Identify the report as a systematic review.	1
**ABSTRACT**			
Abstract	2	See the PRISMA 2020 for Abstracts checklist.	2
**INTRODUCTION**			
Rationale	3	Describe the rationale for the review in the context of existing knowledge.	4
Objectives	4	Provide an explicit statement of the objective(s) or question(s) the review addresses.	4
**METHODS**			
Eligibility	5	Specify the inclusion and exclusion criteria for the review and how studies	5

Section and Topic	Item #	Checklist item	Location where item is reported
criteria		were grouped for the syntheses.	
Information sources	6	Specify all databases, registers, websites, organisations, reference lists and other sources searched or consulted to identify studies. Specify the date when each source was last searched or consulted.	4
Search strategy	7	Present the full search strategies for all databases, registers and websites, including any filters and limits used.	5
Selection process	8	Specify the methods used to decide whether a study met the inclusion criteria of the review, including how many reviewers screened each record and each report retrieved, whether they worked independently, and if applicable, details of automation tools used in the process.	5
Data collection process	9	Specify the methods used to collect data from reports, including how many reviewers collected data from each report, whether they worked independently, any processes for obtaining or confirming data from study investigators, and if applicable, details of automation tools used in the process.	5
Data items	10a	List and define all outcomes for which data were sought. Specify whether all results that were compatible with each outcome domain in each study were sought (e.g. for all measures, time points, analyses), and if not, the methods used to decide which results to collect.	5
	10b	List and define all other variables for which data were sought (e.g. participant and intervention characteristics, funding sources). Describe any assumptions made about any missing or unclear information.	5
Study risk of bias assessment	11	Specify the methods used to assess risk of bias in the included studies, including details of the tool(s) used, how many reviewers assessed each study and whether they worked independently, and if applicable, details of automation tools used in the process.	6
Effect measures	12	Specify for each outcome the effect measure(s) (e.g. risk ratio, mean difference) used in the synthesis or presentation of results.	5
Synthesis	13a	Describe the processes used to decide which studies were eligible for each synthesis (e.g. tabulating the study intervention characteristics and	5

Section and Topic	Item #	Checklist item	Location where item is reported
methods		comparing against the planned groups for each synthesis (item #5)).	
	13b	Describe any methods required to prepare the data for presentation or synthesis, such as handling of missing summary statistics, or data conversions.	5
	13c	Describe any methods used to tabulate or visually display results of individual studies and syntheses.	5
	13d	Describe any methods used to synthesize results and provide a rationale for the choice(s). If meta-analysis was performed, describe the model(s), method(s) to identify the presence and extent of statistical heterogeneity, and software package(s) used.	6
	13e	Describe any methods used to explore possible causes of heterogeneity among study results (e.g. subgroup analysis, meta-regression).	6
	13f	Describe any sensitivity analyses conducted to assess robustness of the synthesized results.	
Reporting bias assessment	14	Describe any methods used to assess risk of bias due to missing results in a synthesis (arising from reporting biases).	6
Certainty assessment	15	Describe any methods used to assess certainty (or confidence) in the body of evidence for an outcome.	6
**RESULTS**			
Study selection	16a	Describe the results of the search and selection process, from the number of records identified in the search to the number of studies included in the review, ideally using a flow diagram.	7
	16b	Cite studies that might appear to meet the inclusion criteria, but which were excluded, and explain why they were excluded.	7
Study characteristics	17	Cite each included study and present its characteristics.	8
Risk of bias in studies	18	Present assessments of risk of bias for each included study.	12

Section and Topic	Item #	Checklist item	Location where item is reported
Results of individual studies	19	For all outcomes, present, for each study: (a) summary statistics for each group (where appropriate) and (b) an effect estimate and it's precision (e.g. confidence/credible interval), ideally using structured tables or plots.	14,20,24
Results of syntheses	20a	For each synthesis, briefly summarise the characteristics and risk of bias among contributing studies.	8,12
	20b	Present results of all statistical syntheses conducted. If meta-analysis was done, present for each the summary estimate and its precision (e.g. confidence/credible interval) and measures of statistical heterogeneity. If comparing groups, describe the direction of the effect.	27,28,29
	20c	Present results of all investigations of possible causes of heterogeneity among study results.	26
	20d	Present results of all sensitivity analyses conducted to assess the robustness of the synthesized results.	N/A
Reporting biases	21	Present assessments of risk of bias due to missing results (arising from reporting biases) for each synthesis assessed.	12
Certainty of evidence	22	Present assessments of certainty (or confidence) in the body of evidence for each outcome assessed.	N/A
**DISCUSSION**			
Discussion	23a	Provide a general interpretation of the results in the context of other evidence.	30
	23b	Discuss any limitations of the evidence included in the review.	31
	23c	Discuss any limitations of the review processes used.	31
	23d	Discuss implications of the results for practice, policy, and future research.	31
**OTHER INFORMATION**			
Registration and protocol	24a	Provide registration information for the review, including register name and registration number, or state that the review was not registered.	N/A
	24b	Indicate where the review protocol can be accessed, or state that a protocol was not prepared.	N/A
	24c	Describe and explain any amendments to information provided at	N/A

Section and Topic	Item #	Checklist item	Location where item is reported
		registration or in the protocol.	
Support	25	Describe sources of financial or non-financial support for the review, and the role of the funders or sponsors in the review.	31
Competing interests	26	Declare any competing interests of review authors.	31
Availability of data, code and other materials	27	Report which of the following are publicly available and where they can be found: template data collection forms; data extracted from included studies; data used for all analyses; analytic code; any other materials used in the review.	31

N/A: Not applicable

Supplementary Table S7. Demographic characteristics of Doxazosin studies

Sr No	Population (Ethnicity)	N	Age (Years)	Drug	Dose (mg)	Dosage Form	Frequency	Method of Analysis	References
**Healthy Population**									
1	Healthy	6	NR	Dox	1	Tab	SD	HPLC	[37]
2	Healthy with Nocturia	24	44–70	Dox	1, 2	Tab	1mg OD for 10 days followed by 2 mg OD for 5 days	HPLC with FD	[35]
3	Healthy	S1 : 24	18–40	Dox	S1 : CR = 8, SIR = 2	S1: CR Tab, SIR Tab	SD	HPLC with FD	[13]

		S2 : 35	18–60		S2 : CR = 4,8, SIR= 4,8	S2: CR Tab, SIR Tab	OD		
		S3 : 24	18–40		S3 : CR = 4,8	S3: CR Tab	SD		
		S4 : 41	M: Y=28 ± 5* E=71 ± 6*, F: Y= 28 ± 6*, E= 68 ± 3*		S4 : CR = 4	S4: CR Tab	OD		
4	Healthy (Thai)	24	18–23	Dox	2	Tab	SD	HPLC with FD	[31]
5	Healthy	26	19–27	Dox	8	Tab CR	SD	HPLC with FD	[63]
6	Healthy	24	NR	Dox	2	Tab	SD	HPLC	[29]
7	Healthy (Chinese)	12	21–25	Dox	4	Tab	SD	LC-MS	[28]
8	Healthy (Korean)	16	M= 22.6± 0.9*, F= 21.4 ± 1*	Dox	4	Tab CR	OD for eight consecutive days	HPLC with FD	[33]
9	Healthy (Thai)	12	21–25	Dox	4	Tab	SD	HPLC with FD	[27]

10	Healthy	8	NR	Dox	4	Tab	SD	HILIC-MS/MS	[38]
11	Healthy	2	NR	Dox	4	Racemic CR Tab	SD	Enantioselective LC-MS/MS	[36]
12	Healthy	24	18–30	Dox	8	Tab	SD	HPLC with FD	[30]
13	Healthy	30	18–50	Fin/Dox	Dox =2, FDC = Dox2mg/Fin 5mg	Tab	SD	HPLC with LC-MS/MS	[34]
**Diseased Population**									
14	Healthy Diseased [a]	20	>20	Dox	1	Cap	SD	HPLC with FD	[40]
15	Mild to Moderate Hypertensive	20	33–66	Dox	1, 4, 8, 16	Tab	OD	HPLC with FD	[41]
16	Mild to Moderate Hypertensive	7	27–57	Dox	2	Tab	SD, OD	HPLC	[42]
17	Elderly Hypertensive (Caucasian)	18	65–82	Dox	1, 2, 4, 8, 16	Tab	OD	HPLC with FD	[43]
18	Hypertensive	25	23–59	Dox	8	Tab	OD	HPLC with FD	[44]
19	Mild to Moderate Hypertensive	10	47–70	Dox	2	Tab	SD, OD	HPLC with FD	[45]
20	Hypertensive	10	39–74	Dox+ CTZ	Dox=1,2,4,8 CTZ =500	Tab	SD, OD	LC with FD	[46]

21	Hypertensive with renal impairment	18	32–66	Dox	1mg titrated to 16mg	Tab	SD, OD	HPLC	[47]
22	Liver Cirrhosis	12	Healthy = 51 ± 10*, Diseased = 50 ± 10*	Dox	2	Tab	SD	HPLC with FD	[39]
**Drug-Drug Interaction**									
23	Healthy Normotensive	12	21–34	Dox + ENL	Dox=1, ENL=10	Tab	OD	HPLC	[50]
24	Healthy Normotensive	12	20–40	Dox + Nif	Dox=2, Nif=20	Tab, Cap	Dox=OD, Nif=BD	HPLC	[51]
25	Healthy	16	25.8 ± 3.3*	Dox + MDMA	Dox=8, MDMA =125	Tab	OD	HPLC	[49]

BD: Twice daily; CR: Controlled-release tablet; Cap: Capsule; CTZ: Chlorothiazide; Dox: Doxazosin; E: Elderly; ENL: Enalapril; FDC: Fixed-dose combination; F: Female; FD: Fluorescence detection; Fin: Finasteride; HPLC: High- Performance Liquid Chromatography; LC: Liquid chromatography; LC-MS/MS: Liquid chromatography with tandem mass spectrometry; M: Male; MDMA: 3,4-methylenedioxymethamphetamine; N: Study size; NR: Not reported; Nif: Nifedipine; OD: Once daily; S: Study; SIR: Standard immediate-release tablet; SD: Single dose; Tab: Tablet; Y: Young

* Mean age ± standard deviation

[a] Diseased population in the mentioned study refers to renal impairment, and End- stage renal failure

Table S8: Comparison of Pharmacokinetic parameters of Doxazosin with other alpha blockers

Parameters	Bioavailability (%)	Protein binding (%)	T_{max} (h)	t½ (h)	Metabolizing Enzymes	Food effect	Major elimination	Dosing frequency
**Doxazosin [26]**	~65	~98	2–3	16–22	CYP3A4, CYP2D6/CYP2C9	Minimal	Hepatic metabolism	Once daily
**Terazosin [27]**	~90	90–94	1–2	12	Minimal CYP	Minimal	Biliary & renal	Once daily
**Prazosin[28]**	~60	97	1–3	2–3	CYP3A4, CYP2D6	Moderate	Hepatic	2–3 times daily
**Alfuzosin [29]**	~64	82–90	8	5	CYP3A4	↑Absorption with food	Hepatic	Once daily
**Tamsulosin [30]**	~100	94–99	4–5	9–15	CYP3A4, CYP2D6	↑AUC with food	Hepatic	Once daily

Table S9: Clinical Dosing and Monitoring Recommendation of Doxazosin

Population	Initial dose	Titration	Monitoring Parameters	References
Healthy (IR formulation)	1 mg qd (Preferred in hypertension)	Double every 2 weeks up to 16 mg/day	BP at supine and standing positions.	[60]
Healthy (CR formulation)	4 mg qd (Preferred in BPH)	Increase to 8 mg after 3–4 weeks	urinary symptoms, BP	[59,60]
Hepatic Impairment (Child-Pugh A)	IR tablet 1 mg qd	Increase dose after 3–4 weeks<br>Maintenance dose <40% than healthy population	orthostatic vitals, LFTs	[61]
Elderly (Healthy)	No regular dose adjustment is required. Closely monitor for the first dose phenomenon.			[64]
Elderly (Frail)	0.5-1 mg once daily (IR formulation is preferred)	Increase dose every 2-4 weeks	Orthostatic vitals	[60,64]
Asian Population	No regular dose adjustment is required. Closely monitor for side effects.			[63]

IR: immediate release, CR: controlled release, qd: every day, BP: blood pressure, LFT: liver function test

Table S10: Drug-drug interaction of doxazosin with CYP3A4 inhibitor and inducer drugs

Drug / Class	Mechanism of Interaction	Predicted Effect on Doxazosin Exposure	Clinical Significance	Recommended Clinical Action
**azole antifungals [36]**	Potent CYP3A4 inhibition	↑ Plasma concentration	Major – increased risk of hypotension, dizziness	Monitor blood pressure closely; consider lower dose or alternative
**macrolide antibiotics [37]**	Moderate CYP3A4 inhibition	↑ Plasma concentration	Moderate – potential enhanced pharmacodynamic effect	Caution in elderly; monitor for postural hypotension
**calcium channel blockers [38]**	CYP3A4 inhibition and competitive metabolism	↑ Plasma concentration	Moderate – potential additive hypotensive effects	Avoid abrupt dose changes; monitor blood pressure and heart rate
Rifampicin, Carbamazepine [39]	CYP3A4 induction	↓ Plasma concentration	Moderate – possible reduction in therapeutic effect	Monitor clinical response; may require dose adjustment

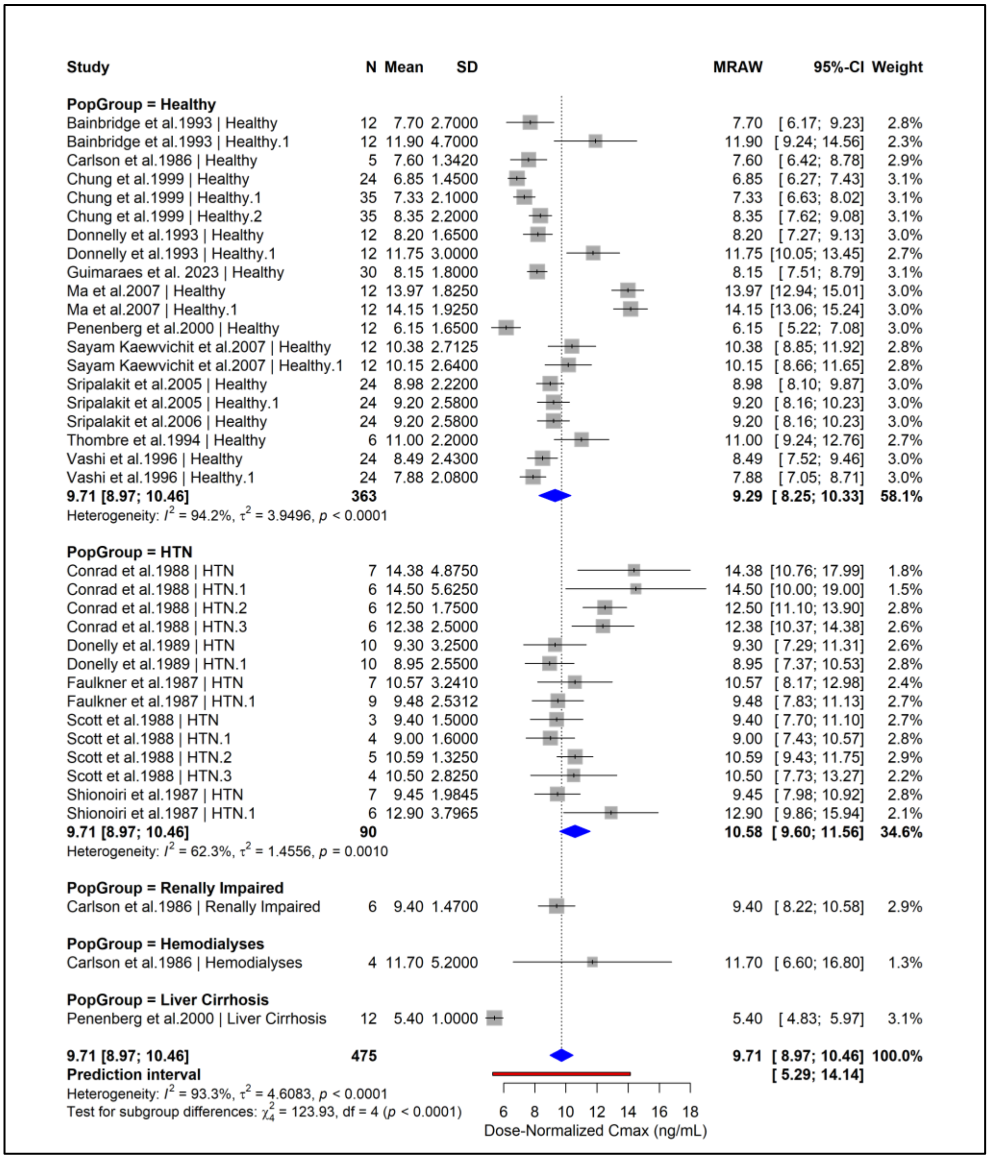

Figure S1. Forest plot of dose-normalized Cmax stratified by population. The figure presents pooled mean Cmax/dose estimates across studies within each population subgroup. Squares represent study-level effects, with their size proportional to study weight. Diamonds indicate subgroup pooled estimates, and the overall diamond shows the total pooled effect. Heterogeneity statistics (τ^2 and I^2) and a test for subgroup differences are also reported. HTN: hypertension [13,27–29,31,34,35,37,40–45,50,51].

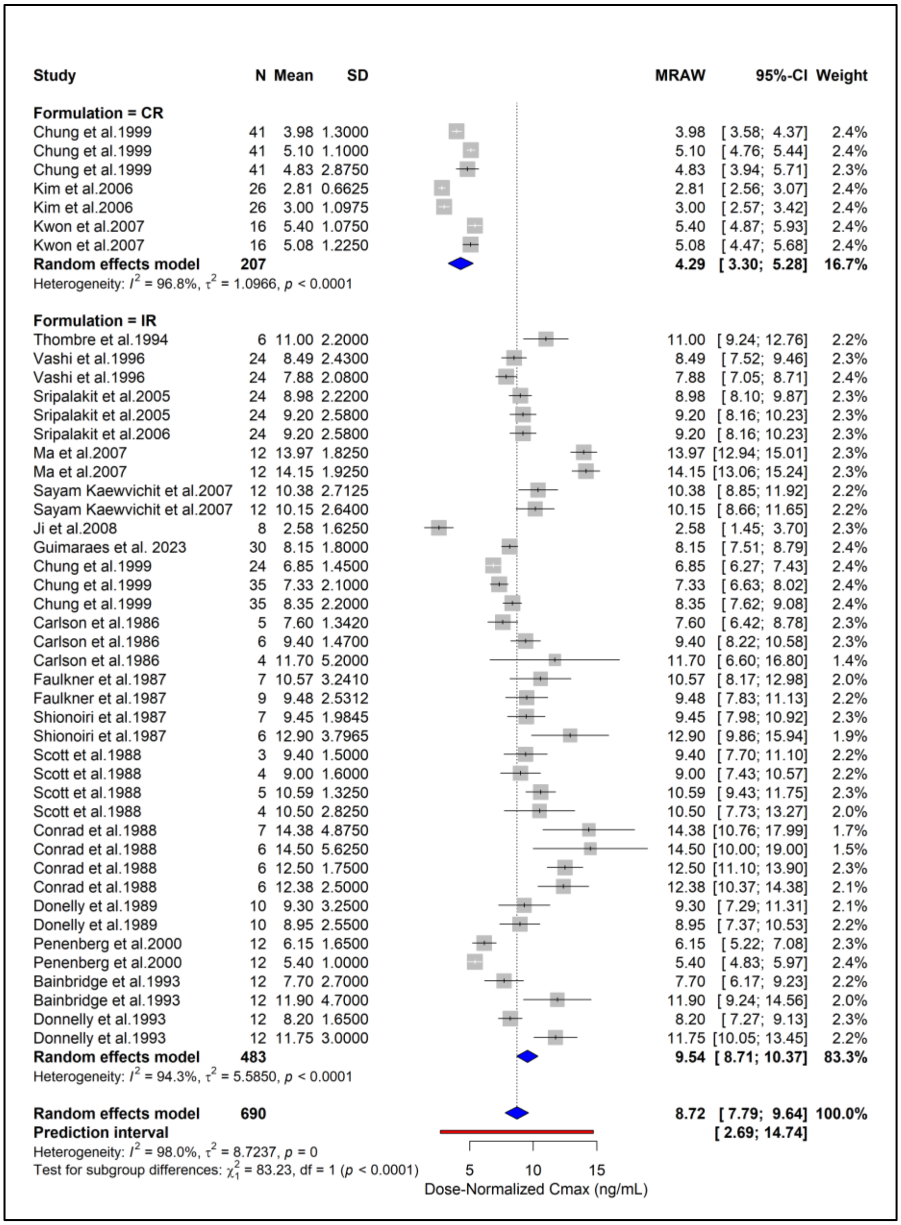

Figure S2. Forest plot of dose-normalized Cmax stratified by formulation type (CR: controlled release; IR: immediate release) [13,27–29,31,34,35,37,38,40–45,50,51].

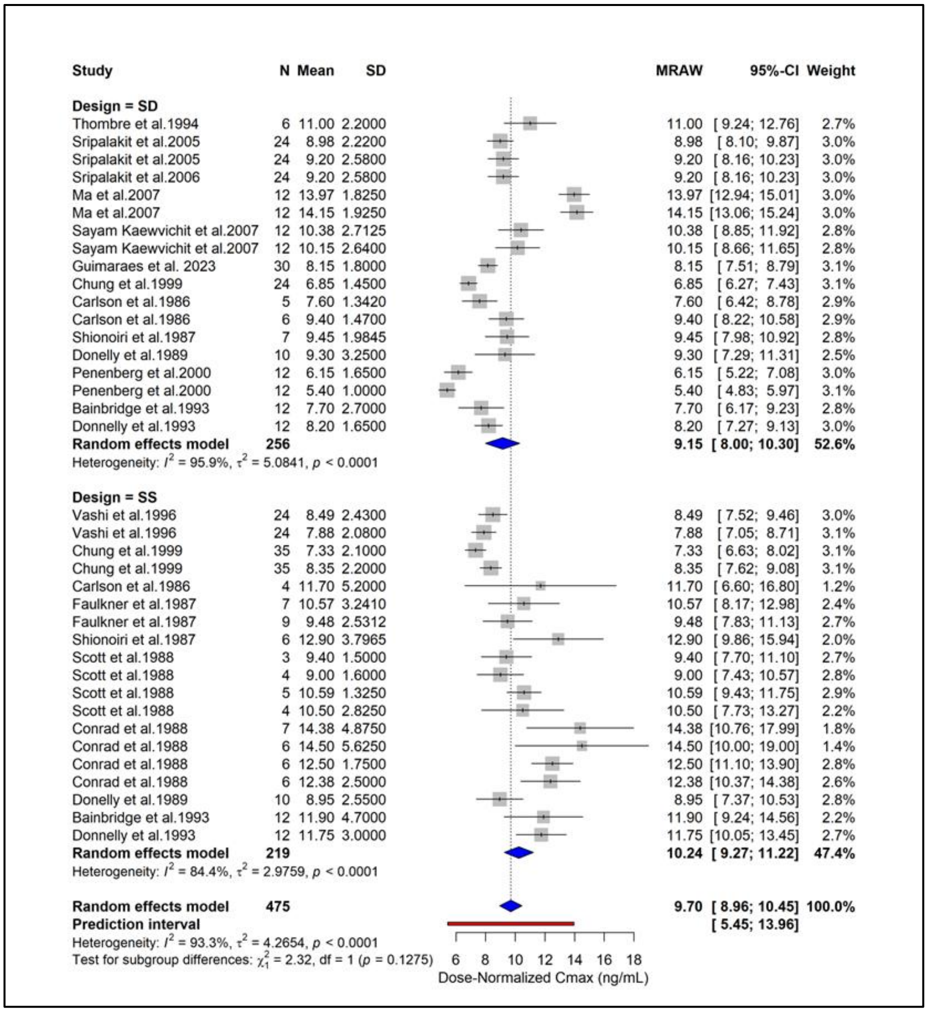

Figure S3. Forest plot of dose-normalized Cmax stratified by study design (SD: single dose; SS: steady state) [13,27–29,31,34,35,37,40–45,50,51].

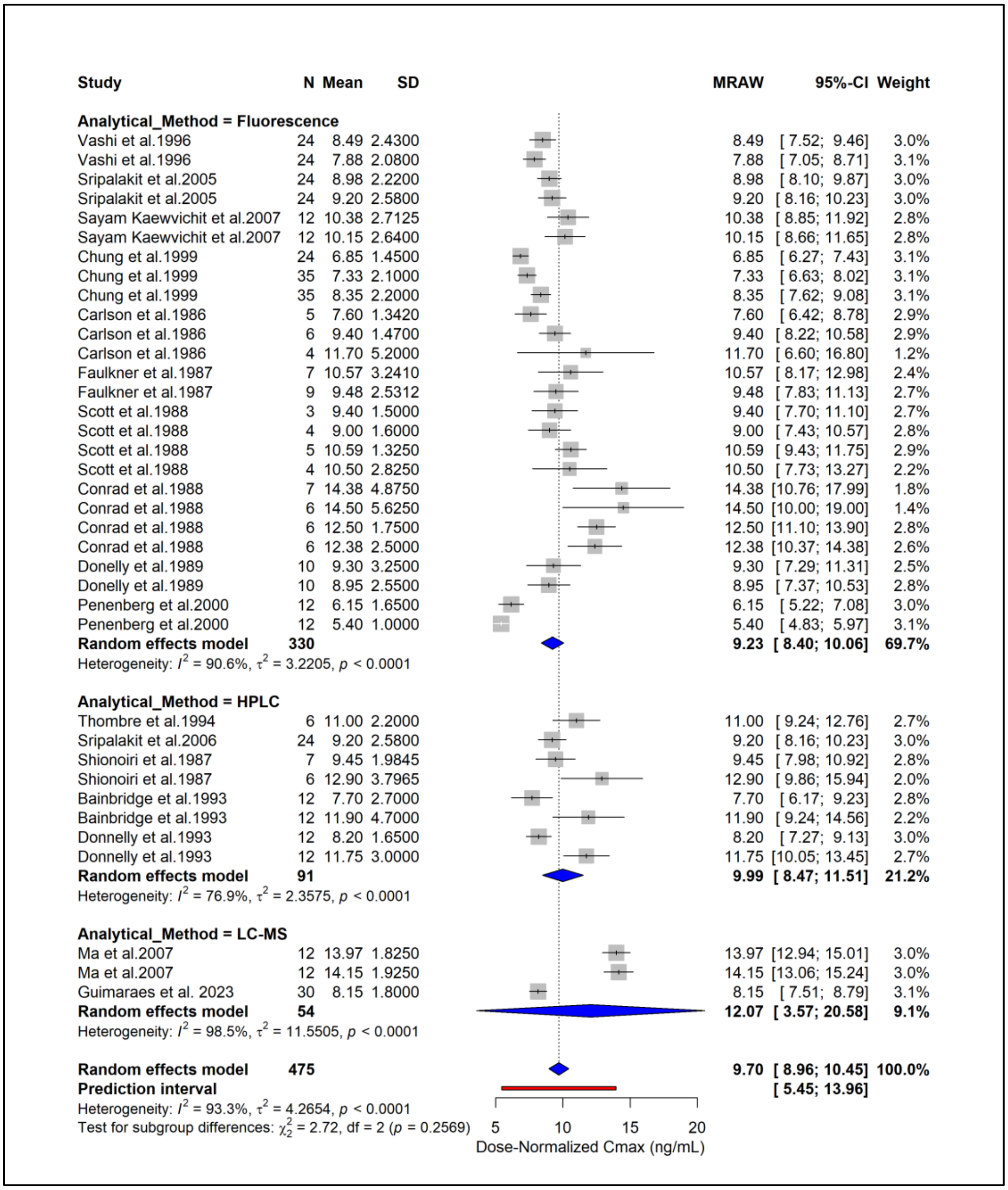

Figure S4. Forest plot of dose-normalized Cmax stratified by analytical method [13,27–29,31,34,35,37,40–45,50,51].

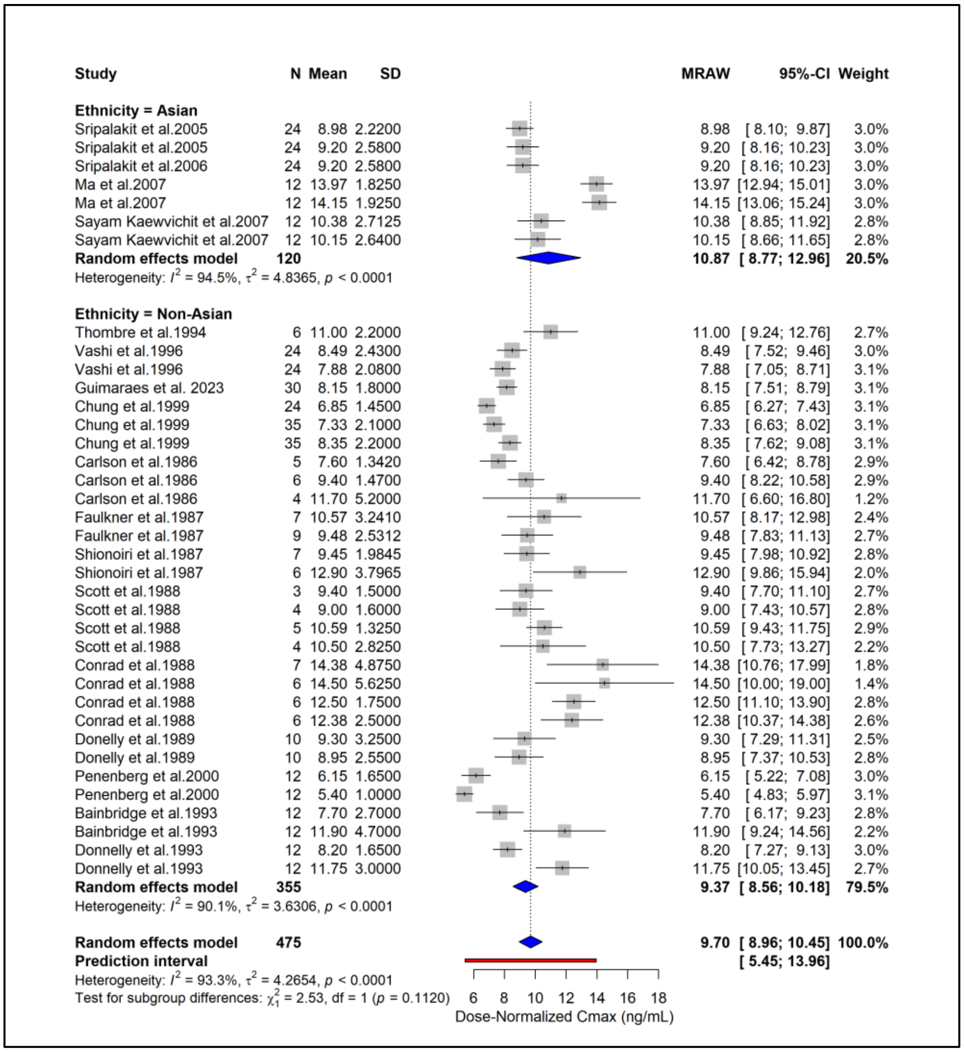

Figure S5. Forest plot of dose-normalized Cmax stratified by ethnicity [13,27–29,31,34,35,37,40–45,50,51].

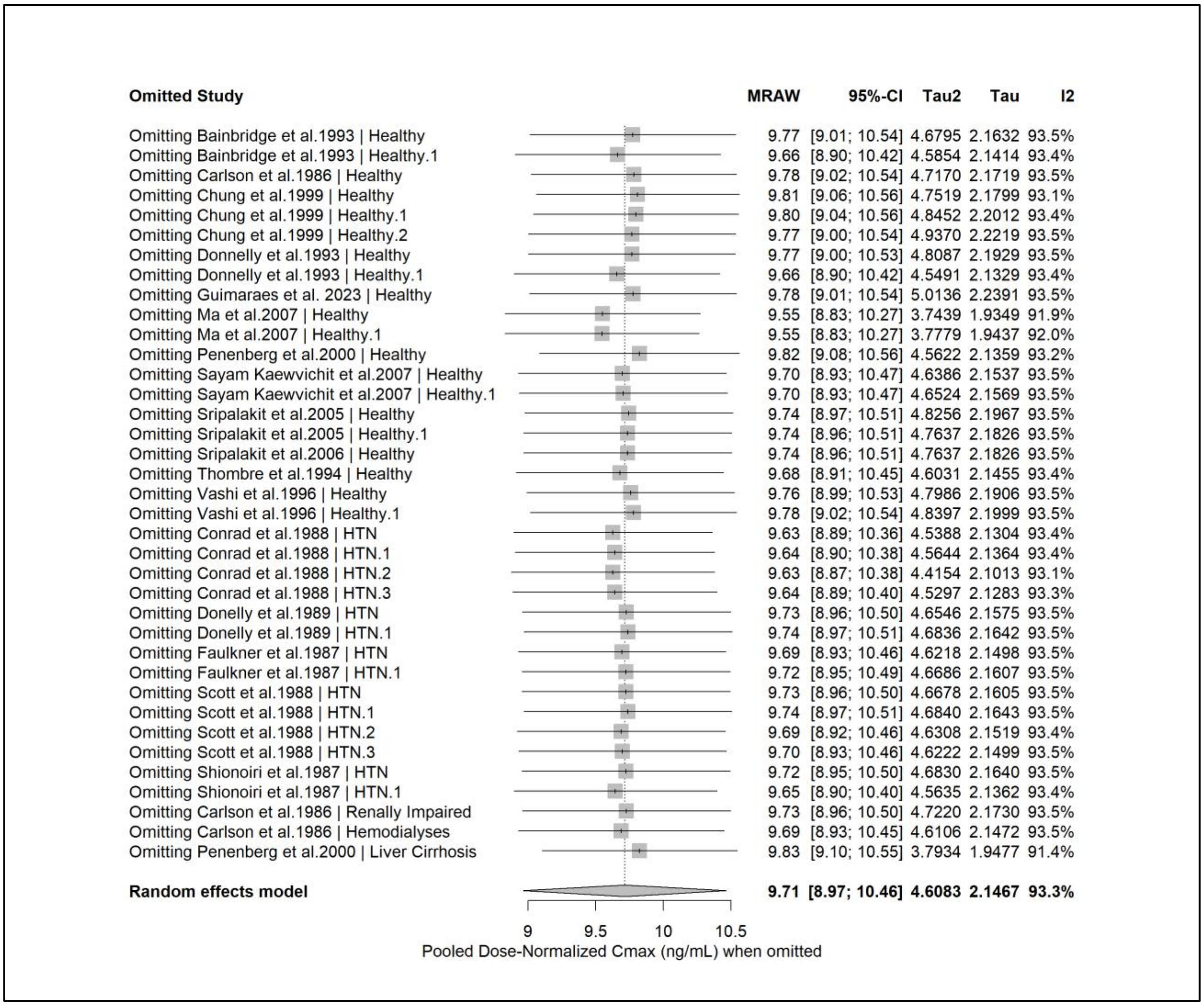

Figure 6. Leave-one-out sensitivity analysis for Cmax. Excluding each study individually did not result in a reduction of I², which consistently remained above 90%. The pooled mean Cmax and its confidence interval remained stable throughout, indicating that no single study disproportionately influenced the overall results [13,27–29,31,34,35,37,40–45,50,51].

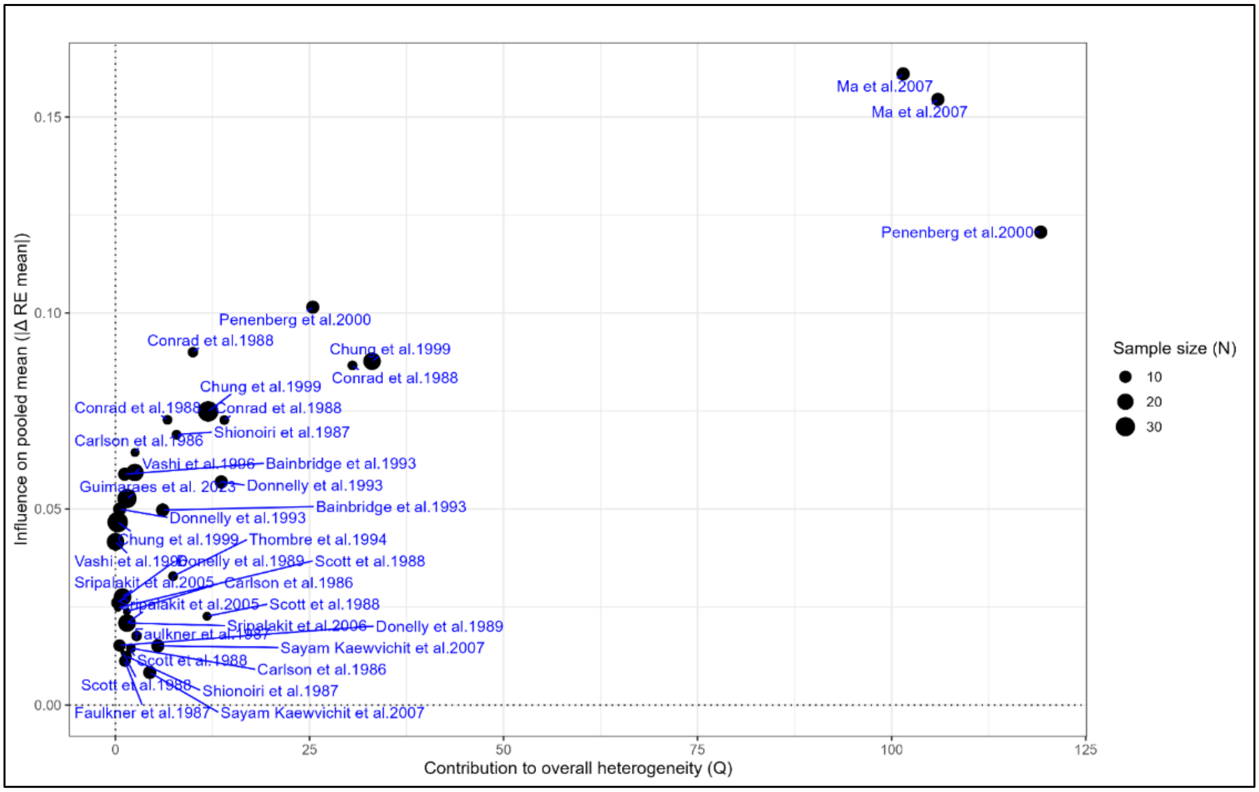

Figure 7. Baujat plot for Cmax illustrating each study's contribution to heterogeneity and influence on the pooled mean. The x-axis represents the contribution to overall heterogeneity (Q), while the y-axis shows the influence on the pooled mean AUC. Point size reflects study sample size. Ma et al. (2007), Peneberg et al. (2000), Conard et al. (1988), and Chung et al. (1999) showed the greatest influence, whereas the remaining studies contributed minimally, supporting the robustness of the AUC findings [13,27–29,31,34,35,37,40–45,50,51].
